# For socially engaged science: The dynamics of knowledge production in the Fiocruz graduate program in the framework of the "Brazil Without Extreme Poverty Plan"

**DOI:** 10.1371/journal.pone.0204232

**Published:** 2018-10-19

**Authors:** Rebeca Buzzo Feltrin, Maria Cristina Rodrigues Guilam, Manoel Barral-Netto, Nísia Trindade Lima, Milton Ozório Moraes

**Affiliations:** 1 General Graduation Office (CGEd) – Vice-Presidency of Education, Information and Communication, FIOCRUZ, Rio de Janeiro, Brazil; 2 Vice-Presidency of Education, Information and Communication, FIOCRUZ, Rio de Janeiro, Brazil; 3 Presidency, FIOCRUZ, Rio de Janeiro, Brazil; 4 Leprosy Laboratory Oswaldo Cruz Institute, FIOCRUZ, Rio de Janeiro, Brazil; University of Westminster, UNITED KINGDOM

## Abstract

Public policy planning associated with the management of the Science, Technology, and Innovation is decisive to improve public health. It is important to develop novel strategies to plan, supervise, manage, use and evaluate research using indicators that extrapolates metrics in current use. In 2011, the Brazilian government introduced the Brazil Without Extreme Poverty plan (BWEP) that aimed to integrate several conditional cash transfer programs (CCT). The original that aimed to integrate of the CCTs were expanded in order to integrate social justice and dignity that induced several actions towards the promotion of social development of the beneficiaries. An induced action involved a partnership between BWEP (From the Ministry of Social Development), CAPES (Brazilian Higher Education Agency) and The Oswaldo Cruz Foundation (FIOCRUZ, a Public Health Institution), that dedicated scholarships for PhD and postdoc students committed to the BWEP to promote health research in its multiple approaches and the vulnerable associated population. Using the Social Studies of Science and Technology (SSST) framework, this paper analyzes the dynamics of knowledge production in the context of program implementation. Herein, we report on the follow-up activities performed in BWEP Health Action, directing research projects to align with the goals of the program, evaluating the progress of these research, and defining strategies for improved their management. We analyze the advances and difficulties encountered in the implementation, monitoring and evaluation of this innovative program in the academic training level, and we emphasize the critical need to expand and improve similar initiatives aimed at guiding the scientific and technological production in health to meet the social demands.

## Introduction

For the past 50 years, and under different aspects, biological and social contingencies—such as death, disease, old age and misery—have been subject to attention and care by societies in different ways. [1]. Since the twentieth century, social protection systems were created in order to face the deep crisis of disintegration of solidarity that is found in modern societies [2].

In Brazil, the goal of eradicating poverty gained strength after the macroeconomic stabilization achieved in 1994 [3]. Ten years after this stabilization, the Bolsa Família Program (from Portuguese, Family Allowance Program, BFP)–a conditional cash transfer program—was created aiming to unify all social programs that had been established in the country since the 1990s. Thus, programs such as Bolsa Escola (created to improve school attendance), or the National food access program, or the cooking gas assistance (for energy availability) and the Single Registration to provide a unified social security identification [3] were combined under the BFP.

Later, BFP was integrated into the Brazil Without Extreme Poverty Plan (BWEP)–from Portuguese, Plano Brasil Sem Miséria—which was considered a milestone in the reorganization process of social programs in Brazil, since it proposed going beyond the mechanisms of cash transfer used by the BFP[3; 2]. BWEP was created encompassing 120 public actions and targeting 16 million people in a situation of extreme vulnerability in 2011 [4]. Poverty can be seen as a complex and multifactorial phenomenon, and BWEP showed innovation by integrating the cash transfer with other emergency and structuring policies, being incremental to previous policies created during the government of President Lula [4]. Thus, the program expands the immediate relief of poverty, and the conditions encourage the use of existing health and education services, improving school attendance and health indicators that were an important strategy to avoid exclusive welfare practices, seeking to move towards the emancipation of families from extreme poverty [5]. National epidemiological analysis has shown the impact of these strategies on the decrease of child mortality in high- when compared to low- and middle-coverage municipalities [6]. The higher coverage BPF in 5-year old child mortality was reduced in poverty-related conditions such as diarrhea or malnutrition, but the benefit of CCT was also observed in chronic infectious diseases such as tuberculosis or leprosy [7].

In this regard, FIOCRUZ (Oswaldo Cruz Foundation), a Brazilian public health institution, was historically devoted to infectious diseases research, control and elimination. In fact, in the early XX century, a combination of innovative laboratorial and public health knowledge contributed to the successful control of Aedes Aegypti-transmitted diseases and effective population mass smallpox vaccination that lead to the eradication of both the mosquito and the endemic circulation of the virus [8].

The need to promote socially relevant research has become a concern in many parts of the world, especially in recent years. Some initiatives and methodologies have been developed to produce knowledge aligned with social needs, such as the Translational Research, Responsible Research and Innovation or the Action Research. However, such ideas were already at the heart of Oswaldo Cruz’s actions and founded the institutional mission of Fiocruz.

At the moment, Fiocruz is participating as a BWEP partner supporting the development of research projects that contribute to overcome problems for the BWEP targeted population [9]. The agreement sought to articulate training, research and technological development that started with an agreement with CAPES, which is the higher education training agency of the Ministry of Education in Brazil. Fiocruz offered 125 doctoral and postdoctoral scholarships to be used for the projects aligned within the themes of the BWEP between 2011 and 2015 [9]. With such a significant number of scholarships offered, the agreement has considerably expanded its funding capacity for graduate research.

This exploratory paper analyzes the dynamics of S&T knowledge production in the Fiocruz academic program in the framework of the BWEP Plan at two levels: first, at the level of the implementation process of the program itself and, second, at the project level, which includes the preliminary proposals of the results for products related to the agreement. Thus, we describe and analyze the implementation of strategies for the continuous organization of the BWEP-FIOCRUZ program, especially regarding to the creation of products / processes that could contribute to the improvement of life conditions of the BWEP target population.

The systematic follow-up of the BWEP researches took place at the time of its development, which proved to be a laborious task, but it substantially contributed to the alignment of the effective management practices used. The project management methodology itself was built in an interactive process of co-construction among the students and managers involved in the development of the partnership.

During implementing this innovative program, some central debates about the models of S & T production and evaluation have emerged, such as: a) What can be considered good science? B) Is it possible to make science and technology socially engaged? C) What role does science and scientists play in current societies? D) How S&T knowledge could approach the real demands of society? E) Can the traditional/quantitative S&T evaluation model assess this socially engage knowledge? F) What are the social impacts of S&T products? We believe that the BWEP agreement experience can contribute to reflect about different S&T production and evaluation ways—specially, in a socially engaged way.

## Methods

This study was developed from the postdoctoral research of the first author, also funded by the BWEP agreement. The researcher was selected by the managers of the institution to provide assistance in orientation and monitoring of studies within the program, and to propose new strategies aiming to reach the objectives agreed upon. The propositional character of the study was based on a fundamental requirement to be reached by the researches funded by the program. The other authors acted as managers of BWEP agreement at different levels in the institution and also contributed as informants in the study.

In this sense, many experiences reported in the paper emerge from the involvement of the authors in the context of the implementing of the BWEP program in the institution simultaneously as analysts and participants. Thus, our place of discussion lies in the context of policy as analysts of the program, as well as in the context of politics [10], from our active participation in the environment of elaboration of strategies for implementing the program. For some researchers of Mertonian tradition [11], this privileged position of speech could be considered problematic, since while it allows a broad (almost unrestricted) access to the analyzed environment, it could bias (or “contaminate”) the results of the research. However, we are aligned with the social constructivist vision of science and technology, which exempts us from the pretension of achieving (in)possible neutrality or impartiality in our analysis.

Since the beginning of the research, the first author (also responsible for data collection) formally presented herself during the events promoted by the program to the research participants. She informed them of their research objectives, their connection to the program and the data types to be collected / analyzed.

The analysis was developed at two levels: a) the broader level of the implementation process of the program itself, especially regarding BWEP products and b) the level of projects, which includes the analysis of the projects developed by the students during the BWEP course. Approaches aligned to the field of Social Studies of Science and Technology (SSST) supported the analysis developed at both levels.

The analysis of the implementation process of BWEP at Fiocruz was based on the observation of two events that brought together the main actors of the program in Fiocruz: postdoctoral and PhD students of BWEP, supervisors/projects, lectures, as well as other researchers and public policy managers—both inside and outside the institution. During the events (both recorded) notes were taken about/regarding the speeches given by the actors.

We assumed that the BWEP-FIOCRUZ program was an organizational technology (or process innovation), and we used the theoretical-methodological framework of social constructivism of technology (SCOT)for the first level of the analysis [12]. In this approach, technology is seen as a result of social interaction in a variety of social circumstances. On the other hand, technology can also influence or condition society actions. Moreover, the technology chosen / established by socially relevant groups is not the “best” one, but one that has managed to reach consensus among these actors. In this context, a meeting between actors could be seen as negotiation space that allowed co-construction of this organizational technology. Jasanoff [13] analyzed a co-construction of S&T under a sociological perspective- strongly associated with the idea of political democracy—and not only under the organizational view. This author suggests that, in order to be inclusive and effectively enable this co-construction, all stakeholders (including civil society members) should discuss their goals and contribute to an elaboration of the final product (which rarely occurs in the traditional mode of scientific production). The author makes us reflect that science works within previously established paradigms, in which we are led to the elaboration of normal / expected research questions based on available methods. Usually, questions that guide research projects are not elaborated in association with the target / studied population, so what are considered social demands are, actually, issues perceived to be relevant by a group of scientists or politicians and aligned with a scientific paradigm [13].

Although the FIOCRUZ-BWEP program has a clear bias that can be considered inclusive, representatives of the studied populations have not taken part in the meetings: the interface with this people was made by the researchers during fieldwork.

In the level of the projects analysis, we use three approaches to manage and follow-up the research of the students directing them towards BWEP aims: 1) the evaluation of the subject of the projects presented in the Abstract book of the BWEP Seminar(S1 File), 2) a survey applied to the PhD and postdoc participants of the BWEP (involving 110 respondents), 3) the discussion and orientation of the final work (products of research) presented in the course offered to BWEP students (involving 50 PhD/postdoc attendees between November / 2015 to December / 2015).

Therefore, the Actor-Network Theory (ANT) provided the necessary support for the intended analysis. According to Latour [14; 15], for a knowledge to be effective translated it needs the support of a sociotechnical network that involves human and nonhuman actors. In this sense, the agreement provided an important part of the material base for the progress of research (through scholarships), as well as inducing the formation of networks of technical cooperation and, especially, policy to enable such studies. Thus, the political articulation was facilitated in several ways, creating conditions favorable to the progress of the research, such facilitating contact between researchers and public agencies of the municipal, state or federal administration. The opening of a channel of direct communication between the various actors involved in the institution and the coordination of the program can be highlighted as one of these improvements.

The negotiations during this phase allowed researchers and decision-makers to be brought together, creating forms of interaction between groups and promoting a more collaborative, reflexive and democratic work.

## Results and discussion

In this section, the main results about the management/follow-up of the projects and the analysis of the implementation process (macro level) will be presented.

From the exploratory analysis gathered in the Abstract book of the Seminar “Graduate program in Fiocruz and the Brazil Without Extreme Poverty Plan” of 2013 –it was possible to identify the focus of the BWEP projects. The mapping of ongoing research (fellows and non-fellows) allowed us to identify six main thematic axes: 1 –Control, monitoring and treatment of diseases related to poverty; 2 –Epidemiological aspects and strategies for diagnosis of diseases; 3 –Populations at risk; 4 –Educational actions in health; 5 –Evaluation of public policies and 6 –Relations between Science, Technology and Society. It is important to emphasize that many papers could be framed in two or more thematic axes simultaneously, due to their interdisciplinary nature. However, to facilitate the mapping of the themes, we tried to categorize each work according to its main thematic axis.

Overall, the covered topics fulfilled the objective recommended in the Technical Cooperation Agreement to generate knowledge aimed at solving problems related to extreme poverty. However, the majority of the research focused on “diseases of poverty” (49%)–divided into the thematic areas “Control, monitoring and treatment of poverty-related diseases” and “Epidemiological Aspects and Strategies for Disease Diagnosis”. Other topics considered strategic were under-represented, for example, the themes related to “mental health and poverty”; “Maternal and child care” or “support from the homeless people” had little or no projects until then, demonstrating the need to stimulate research that developed such themes.

In addition, there was a repetition of themes within each thematic axis—in the “Evaluation of Public Policies” axis, for example, the work focused on evaluating programs such as “Bolsa Família” and Health in School, while other programs were not even mentioned among the surveys, such as the “Mulheres Mil” (program for economic inclusion of women). Such distortions were a direct result of the decentralization of the management and monitoring of this CAPES-BWEP program carried out in each Institute. In the first mapping it was possible to identify the distribution of the BWEP researchers throughout the different Fiocruz research institutes. There was a significant concentration of participants (55%) related to the biomedical research institute called Oswaldo Cruz Institute (Portuguese acronym, IOC), while other institutes did not have participants (scholarship holders or not) linked to BWEP.

The results of the research mapping were presented at the meetings between the actors involved in the project with the purpose of discussing the next strategies. The meetings became an important channel for managers to identify the needs, doubts and questions of the various relevant groups involved in the agreement, making it possible to validate, refute or restructure their action strategies.

In the field of SSST, so-called laboratory studies allow us to ethnographically monitor the dynamics of knowledge production in the main context in which it is constructed, so that we can grasp the complexities that permeate this production. Thus, meetings with relevant groups (promoted events and course meetings) have become a privileged locus of analysis of an important part of the knowledge production dynamics.

The debate among the actors was stimulated to discuss strategies to integrate, strengthen and expand the research and actions developed through the BWEP-FIOCRUZ program. Two strategies were especially discussed during the meetings: 1- the need to guide the research of the agreement to the development of products /processes/methodologies with direct social application to the beneficiaries of the BWEP, and 2 –the BWEP product definition itself, issue that had not been explained until then by several institutes of the Fiocruz.

The debate over what should be a “product” that is beyond academic thresholds, heavily admitted as scientific papers, generated discomfort among the BWEP participants. Although everyone understood and supported the need to apply the knowledge for the benefit of the BWEP target population, the need to create an “extra” product was traditionally expected by graduate programs, or the research evaluation framework. The very definition of “product” for CAPES—FIOCRUZ agreement was considered problematic. Many researchers were unable to identify what could be a product of their research, in addition to publications.

Such resistance by the researchers that the evaluation of their production is not seen in a positive way by their peers: what is considered “good science” is usually related to formal publications, regardless of the quality of the research or the designing of solutions / products of social relevance. Moreover, non-traditional products of science are often intangible, but not less important. The traditional S&T evaluation model does not give space for the emergence of such products, making them invisible.

In fact, this model of S&T evaluation encourages the scientist to work increasingly isolated and disconnected from the real needs of the society around him. In addition, it neglects important themes for social development, turns relevant forms of knowledge invisible to their populations/contexts, and enhances knowledge/products that often satisfy only particular interests. Usually, the choice of research subjects is aligned to a global agenda, and issues of extreme relevance to the local context can be neglected. In the studied case, the research themes (related to the eradication of poverty) tend to occupy a marginal position in the scientific field (or may be neglected), not receiving the same recognition themes considered “noble” (those that also affect developed countries).

It is important to emphasize that the products of BWEP did not necessarily focus on innovation (in the traditional sense), but on the application and utility of scientific and technological knowledge for solving social problems, which in a way can also be understood as innovative health policies and applications. The definition of the BWEP products was not satisfactory and many researchers went on to exemplify cases of possible products within their own research. In fact, the deeper reflection on the social role of S&T is not part of traditional scientific education itself, directing the perspective of the future researcher to respond to the productivity rewards system, which assumes, on its premises, that quality research, sooner or later, will bring social benefit [16].

Although scientists continue to emphasize that they produce science for the benefit of society, an argument that is used especially when applying for public funding, the fact is that in practice little has been done [17]. Publications play a fundamental role in the production of science, but they must be seen as stages of research and not as their end. The aim of science applied in health should be vaccine production or the organization of health programs and should not end in the publication of a paper [18].

This first effort of analysis, discussed with BWEP participants, allowed managers to know the specificities of the research, making it possible to propose measures to stimulate new research that would fulfill the gaps identified together with the other groups. Thus, a permanent, active and collaborative evaluation among the different actors involved was encouraged. The negotiating space favored by the meeting provided an intense exchange of information between the actors involved on the direction of the BWEP program. The groups were able to expose their expectations, difficulties or identify limits and the negotiations were stimulated until approaching a "consensus". Pinch and Bijker [12] show that the different groups involved in the debate may present different expectations about the same technology (in this case, the program itself), giving it completely different meanings, the so-called interpretive flexibility. The divergence of opinions generates conflicts of interests between the groups and ends up being decided by a cycle of negotiation and renegotiation, until it comes to a process of stabilization and closure of the controversies (consensus).

During the first BWEP meeting (September/2014), all the controversies surrounding the discussions about the need for products linked to BWEP research may ultimately lead to a stabilization process, albeit preliminary. In this case, the stabilization referred to the acceptance of the need of the product as fundamental for the research linked to the BWEP, sealing the commitment between managers and researchers of the program to comply with the established goals. It is worth mentioning that, since the managers involved in program monitoring did not participate in the formulation phase of the actions agreed, there was a limited possibility of intervention in the objectives of the actions during the implementation phase: it was restricted only to negotiation the way in which previously agreed upon goals would be met. However, this fact did not become a definite barrier to the progress of the agreement. Although the researchers involved challenged the obligations of the program, they attempted to adapt the objectives set and could participate in the (re)definition of the concept of the product itself in the BWEP context. At that time, there was no clarity on the part of the program’s formulators about what could be considered a "non-traditional" S&T health product or even what was expected as a result in the program, and then this gap provided a space for negotiation, where the meaning of the "product" can be refined and constructed together with all the actors involved. Another example of such a negotiation or co-construction of concepts around the product may be evidenced, for example, by the proposition that each thesis / research is not required to generate a related product but that a "larger" product could be created as a result of two or more BWEP researches.

### Strengthening the engagement to BWEP outcomes

During the follow-up and management of the projects, the need to elaborate a form directed to all the FIOCRUZ-BWEP participants was observed, so that they had the opportunity to express their opinion about the development of projects and to indicate the thematic axis that most resembled their research (self-declaration). The application of the form gave voice to the researchers involved (and capture the "interpretive flexibility" of this group), contributing to a more realistic and collaborative evaluation of the results of the agreement, and validation of the strategies proposed by the program coordination in the institution.

The follow up form of the agreement was applied to researchers participating in the BWEP between December 2014 and February 2015 and received response from 110 out of the 114 participants that were active in that period (105 fellows and 5 non-fellows). The form was forwarded through the institutional email and used the *Googleform* online tool.

The form covered 14 open and closed questions that included the framework of the research in the defined thematic axes, current and potential collaborators, doubts / criticisms and suggestions, beneficiary populations, products / processes derived from the research, as well the student identification, and the research description. The main questions of the form were structured to meet the needs identified by the program managers. However, several themes / subtopics emerged from the speeches of the actors during the meetings. Other analyzed themes were pointed out by the researchers themselves in the form, opening the possibility of new debates.

Among the difficulties encountered by some researchers, the indication of the products / processes derived from the research and the process of organization of the institutional technical notes were quite mentioned, and were subsequently included as priorities in the proposal of new strategies.

The linkage of the product to BWEP was reopened, again being identified as a problem by the participants of the agreement. Several fellows complained that they did not know clearly what was expected as a research product other than their scientific publications or theses. Even with the reported difficulty, the majority of the researchers indicated in the form more than one product / process derived from their research, and only 17 indicated only one product.

The most frequent combination of products was "institutional technical note" and "informative / communicative materials", totaling 16. Curiously, some participants provided strictly academic nature as products such as scientific papers (n = 9). Other options indicated by the researchers included: Health worker training workshops, production of process indicators, evaluation matrix of health promotion programs and development of handouts.

Technical notes on public policies have a fundamental role in subsidizing public health, "translating" the academic knowledge that was generated into practical recommendations for policy makers. Likewise, informative and communicative materials seek to approach local communities, "translating" the academic knowledge produced into a more accessible language to approach these populations. Like the other products mentioned, it can be observed that the demand to link “socially applied products" to “academic products" have helped to stimulate the transposition of boundaries between Science and Society in general.

### New negotiations between actors and refinement of strategies

The results of the form, the new integration strategies and the reorientation of the research were presented during the Second BWEP Meeting in May / 2015. Due to the resistance and difficulties pointed out in relation to products that were not considered "traditionally academic" by the coordinators and postdoctoral students during the first meeting and expressed in the form by the other BWEP Fellows, 4 researches that already presented results / advanced products and well delineated (within different thematic axes) and that were well aligned with the objectives of BWEP were selected to aid in the debate and also to stimulate the other students who had not yet been able to develop their products. The selected researches (listed below) were presented to colleagues during the second meeting:

1. Creation of Youtube channel with contents destined to the health area for training of health professionals and dissemination to the general population.
2. Social factories as an inclusive methodology for coping with intestinal parasitosis in a hyperendemic area of precarious housing in the District of Murinim, Benevides, Pará.
3. Rapid and low-cost diagnostic test of *Trypanosoma cruzi* infection in human population in endemic areas. Obtaining a new *chimera protein* to use in a rapid serological diagnostic test of *Trypanosoma cruzi* infection in human population in endemic areas.
4. Observatory of the Technical and Technological Training Policy of the Federal Institutes to create a diagnosis of the insertion and productive inclusion of the skilled workforce through regular and technical secondary courses offered by the FIs in the areas of BWEP.

The presentations were very well received by the students, since they served to give materiality (in the sense of illustrating) which would be considered a product that was well aligned with objectives of BWEP.

As discussed earlier, it is unusual for young scientists to be instructed to produce relevant results for society. The authority and legitimacy attributed to S & T in our society should lead research to assume its responsibility for social transformation in an equally expressive way. However, the S & T production model based on the view of a neutral and autonomous science often serves to reinforce social inequalities rather than to combat them. In view of these issues, program management had to face the challenge of sensitizing BWEP scholars about the role of science in society, on the objectives of BWEP and on the institutional mission itself developed since Oswaldo Cruz. To meet this demand, a course was proposed to BWEP researchers assigned to the different programs, which was offered between November and December 2015, entitled "Science, Technology and Society".

### BWEP course and its products

The aim of the BWEP course was to discuss the relations between science, technology and society, trying to stimulate the students bound to the agreement to think about their contributions to society through their academic research. The course was organized through lectures given by experts on topics related to the thematic axes of BWEP.

The experience of the course was extremely innovative in the institution, considering that it gathered students from different areas, and Institutes of Fiocruz that were subjected to a common environment. Due to its innovative character, this interdisciplinary and inter-unit course required some adaptations by the institution. For example, the course offered web transmission so that students from other Fiocruz Institutes (outside of Rio de Janeiro) could follow and validate their credits in the course.

Parallel to the offer of the course, a virtual community was structured in the Moodle Platform, in which the students had access to the documents / analysis reports of the program (S2 and S3 Files), to the results of the online form, and where they could access the summaries of the researches of other colleagues of the program. In addition, students were able to view presentations of the classes, to send their works through the system, to have a calendar of events of the program, and to obtain a channel of direct access to the management of the agreement in Fiocruz. This tool was used to stimulate the approximation, cooperation and integration of the researchers of the program, favoring the communication with the coordination of the program in Fiocruz and stimulating the students to stay in contact even outside the classes.

Initially, the first class was scheduled to be a workshop on the preparation and submission of an Institutional Technical Note, following a request from the students themselves using the applied form. However, the guest suggested to broaden the debate and the presentation of the theme "Integrating Basic Research, Translational Medicine and Public Health", which would also cover the discussion of other potential health research products, as well as the technical note. In fact, extra-scientific factors, as Latour points out [14; 15], are an important part of the construction of scientific knowledge and deserve great attention on the part of the researchers. The students bonded and got involved in the debates presented by the lectures. Many of them gave a positive feedback to the coordination of the program, thanking the experience of the course. Some students even pointed out that they had no other similar opportunity to reflect on the role of S & T in their courses of origin and that such initiatives should be offered more often.

The organization of the course allowed the cooperation and integration of the BWEP researches in an interdisciplinarity way, encouraged by the approach of the students during the meeting of the classes that, although they were in distinct units, areas or spaces, possessed Research aimed at similar themes and / or aimed at the same target audience. In addition, the organization of the final work of the discipline was aimed at mapping the actions and products of the BWEP in development or already consolidated, enabling practices with positive results to be replicated in the future. The final work could be presented individually or in group following a pre-defined script, stimulating, once again, the cooperation between the researchers.

After the course, students were more open to incorporate the idea of creating "non-traditional" products as the results of their scientific research. By analyzing this process, we observed a refinement in the definitions of the BWEP product concept. In this context, as the students attempted to articulate and define what they hoped to be a product of BWEP, the process itself triggered a deep reflection between them, altering their way of thinking about the social contribution of science.

Because the courses had characteristics that raised students’ awareness such as transversal disciplines, the evaluation of the paper encompassing distinct fields of knowledge and, consequently, products so different from each other was a great challenge. Thus, there was a need to shift attention to the products themselves and to analyze how the discussions of the course was incorporated by the students in the description of their products and in the strategies to put them into practice.

The way the researchers described and justified their products with a social bias was particularly interesting, since some students (especially from the biological and medical areas) started to incorporate a more sociological framework, aligned with the discussions promoted in the course. The strategies adopted to create and disseminate their products were also analyzed—the articulation with other actors to have certain accesses, the creation of their own networks of cooperation inside and outside Fiocruz to assist in the research and diffusion of generated knowledge, the involvement of the beneficiary population of BWEP in the construction of knowledge and the attempt to predict a positive social impact are just some of the contributions that could be identified as a direct result of the stimulus and debate promoted by the discipline.

If the proposal of the product was made in a group, there should be coherence between the research involved in the creation of the collaborative product—be it with the same target audience, or within the same theme, or any other link that could be justified. Cooperation between students was facilitated, especially between different areas of knowledge and units of Fiocruz, but the final choice was left to the discretion of the students. Although the creation of a product could initially look simpler, it depended on the availability and articulation between the students to find common products.

In total, 43 students were enrolled as regular students in the course, out of which 37 were individuals and 6 were working in groups (five groups working in pairs and one in trio). Group product proposals could also offer some advantages to researchers. As discussed earlier, a product to be constructed and applied for its intended purposes depends on a series of "extra-scientific" factors, which involves articulation with a complex network of actors. To "enlist" different actors to follow up on their theories or products, the researcher must use the "translation" of his/her research objectives in a comprehensible language for the adhesion of other groups. In this sense, the product can be understood as a first step towards this knowledge translation. When a research product ends up generating an institutional technical note, a recommendation to decision-makers or even information material for the community, for example, it is nothing more than a translation of the scientific research aimed at "enlisting" other actors [14; 15].

Under the agreement, the necessary support was provided for establishing the networks themselves by the researchers and part of the material conditions to elaborate the product, however, such benefits or facilitators did not guarantee the "success" of the product, nor the expected social impact. After all, products, technologies, or "scientific facts" that are considered "winners" are not those that are considered the "best", "most applicable" or "most innovative", but those who have enlisted more actors to fight for their vision. In this sense, the management of the program focuses on supporting these other activities that are crucial to the development of products, since it is guided by a vision of science that recognizes such "extra-scientific" processes as inseparable from the conception of science itself.

Moreover, the character of BWEPs product reinforces a position that what is considered “scientifically better" is not always the best for socially excluded groups. In such ways, the BWEP product attempts to bridge the gap between science, technology and society by narrowing the links between these spheres traditionally seen as separate.

As a result of the final work of the discipline it was possible to notice three distinct categories of products, considering their heterogeneity, distinct areas and disciplinary fields:

1. *Products directly linked to the results of the research(es)*, *according to selected sample*:
  - Develop a diagnostic kit for flow cytometry with molecularly defined antigens to contribute to the diagnosis of American cutaneous leishmaniasis
  - To qualify programs for the care of the pregnancy, delivery and postpartum of indigenous women and children, particularly Mbyá-Guarani, in order to give effect to the right of these peoples to have a differentiated attention to their health. Development of didactic-pedagogical and intercultural products for differentiated education in health, for the promotion of well-living and for the dissemination of elaborated indigenous cultures.
2. *Products directly linked to the results of the research(es) and with estimated social impact*, *according to selected sample*:
  - Application and evaluation of the impact of educational actions on the indicators of schistosomiasis infection in school children from a municipality with a high percentage of poverty located in the endemic area of Minas Gerais.
  - Expected impact: A Special report will be sent to the MoH recommending the use of these actions as measures of prevention and control of schistosomiasis. Another product to be made available will be a script (printed version and online version) with all the stages of development of the educational process with the materials developed so that they can be reproduced and / or adapted by professionals of other endemic areas of schistosomiasis. The strategy developed in the research can be effectively translated into national policies, incorporated into the Health in School Program (PSE—Decree 6,286 of 12/5/2007). Technical Note properly approved and endorsed by the direction of the Oswaldo Cruz Institute and the presidency of Fiocruz will be elaborated with recommendations on public policies for the surveillance, prevention and control of schistosomiasis.
  - To evaluate the performance of the Reverse Transcription (RT-qPCR) technique using the Mycobacterium tuberculosis 85B gene in the detection and response to specific treatment of pulmonary tuberculosis.
  - Expected Impact: Rapid test in which the result can be released within 48 hours, such as RT-qPCR, reduce the time from 60 to 32 days by disregarding the sensitivity test, which usually takes around 30 days. This test may greatly aid in the monitoring of treatment, being especially important in the prevention of the transmission of resistant Tuberculosis.
3. *Other products with link to the results of the research(es)*, *as shown below*:
  - Development of a road map to promote the divulgation of the Macular Fever in virtual environments: web pages or mobile applications
  - Writing a biography of the life of extremely poor obese women to understand the social scenario related to being the holder of the Bolsa Família Program—their eating and healh experiences, and survival strategies adopted throughout their lives.

Category #3 products were generally linked to the production of informative and communicative materials. Not all the products that produced informative / communicative materials were in this category, but it was possible to identify that when the researchers could not clearly see the products of their research, they suggested the creation of these materials.

Another explanation for this may be again associated with the question that the widespread publication of results is at the heart of the traditional scientific mode of production and evaluation, making the researchers prioritize a "publicizing" of their results. So, when faced with the mission of creating a more direct application product that would "translate" the knowledge produced in their research, publishing for the lay public may have looked like the easiest solution. Thus, between BWEP products and traditional S&T products, there would be only a shift from the target group (from scientists to society in general or to managers).

Although they have fallen short of expectations, at least there has been an attempt to dialogue with audiences other than the academic. Scientists are habituated to produce knowledge with no dialogue between the rest of society, as if science did not need to or should not "contaminate" itself with external issues to it to preserve its pretended autonomy. This thought is linked to the view that the solutions for social problems will come exclusively from science and will be applied in society without the need for any dialogue or justification (from top to bottom), since science would have the capacity and legitimacy to judge what would be better for the population. Even so, the emphasis on informative / communicative materials as a BWEP product shows us that there is still a long way to go.

Other informative / communicative materials included in the students’ work were related to the sensitization of the target populations of BWEP in the following themes: Chagas’ disease, Macular fever, vector control, Toxoplasmosis, Intestinal parasites, Aedes aegypti, Schistosomiasis and Leishmaniasis. Another fact to be punctuated was the creation of several similar materials—several informative materials about the same disease / theme, with the same objective, and that only altered the target audience or the region to which the material was destined. An example of this was related to the informative / communicative materials on Leishmaniasis, with 4works in total, where 3 were focused on general information on the disease and the fourth on the co-infection of Leishmania and HIV.

On the other hand, the researches whose products were more aligned with social demands, especially those that had already been put into practice and / or achieved some social impact, were not exclusively based on scientific rigor, but also on the ability to articulate with other actors and more extra-scientific factors. Involvement with the target audience in the construction of products also seemed to be decisive for the "success" of the product and possible future social impact.

We believe that the experience of the BWEP agreement contributes to a reflection on alternative forms of S&T production also considering that the parameter for an evaluation of knowledge should be equally sensitive to the specificities. In this sense, some relevant practices experienced during the implementation process and / or considered desirable can be highlighted: 1- focus on solving problems connected to the local context, and not following only an agenda of global research, 2- establish bridges between STS, especially, seeking to subsidize a creation of more inclusive public policies (social engagement), 3- Consider that the evaluation of S&T should be sensitive to products other than traditional ones, such as reports, technical notes, local solutions / actions / practices, etc.; 4—Question the science production in a supposed rigid division between basic and applied science; 5-Stimulate S&T production aiming towards the local demands in the training level (graduate); 6- Target efforts and resources for further follow-up of the research (long term impact of research).

Given the numerous reflections that emerged from the implementation of the program, BWEP’s experience has been quite rich in questioning (and challenging) the very model of science and society we want. The program is innovative in several aspects and it is hoped that initiatives like this can become increasingly present in the spaces of production of scientific and technological knowledge, especially in developing countries. The main managing points are presented in Table 1.

**Table 1:** Strategic actions that were developed to supervise and manage BWEP program in FIOCRUZ.

ACTIONS	GOALS	INDICATORS
**Evaluation of the subject of the projects presented in the book of abstracts (BWEP Seminar)**	Map the projects (fellows and non-fellows) to identify the main thematic axes and possible thematic gaps	110 projects were identified in six main thematic axes: 1—Control, monitoring and treatment of diseases related to poverty; 2—Epidemiological aspects and strategies for diagnosis of diseases; 3—Populations at risk; 4—Educational actions in health; 5—Evaluation of public policies and 6—Relations between Science, Technology and Society.
**Organization of two meetings (September/2014 and May/2015) between the main actors involved in the BWEP**	Discuss the strategies of implementation and the orientation of BWEP actions. Identify the needs, doubts and questions of the various relevant groups involved in the agreement, making it possible to validate, refute or restructure their action strategies. Two main strategies were discussed during the meetings: 1) the need to guide the research of the agreement to the development of products and 2) definition of BWEP products.	The definition of BWEP products and the organization of new strategies to guide BWEP researches were co-constructed with the relevant groups during the meetings.
**Elaboration and application of the agreement’s follow-up form directed to all the CAPES-FIOCRUZ BWEP participants**	Identify the general situation of the researches in development, including: the framework of the research in the defined thematic axes, current and potential collaborators, doubts / criticisms and suggestions, beneficiary populations, products / processes derived from the research.	Survey applied to the participants of the BWEP (involving 110 respondents) between December 2014 and February 2015.
**Creation of a virtual community in the Moodle Platform**	Stimulate the cooperation between the BWEP researchers	50 BWEP researchers were enrolled in the Moodle community—from October / 2015
**Organization of BWEP discipline “Science, Technology and Society”**.	Stimulate the students bound to the agreement to think about their contributions to society through their academic research.	Inter-unit course offered between November / 2015 to December / 2015 with 9 classes (guest lecturers) to explore subjects of interest to BWEP—50 students was enrolled, 43 as regular students.
**Organization of the final work of the BWEP discipline—description of the intended product and its possible social impacts**	Map the actions and products of the BWEP in development or already consolidated, enabling practices with positive results could be replicated in the future.	Products of research– 37 individual works and 6 group works (5 in pairs and 1 in 3 students) were presented.

## Final considerations

The paper followed the dynamics of knowledge production within the context of Fiocruz’s agreement with the BWEP. The agreement expected as a result of the researches the generation of products of direct social application, which are not contemplated by the traditional system of production and evaluation of S&T. However, these products were not usually prioritized by the researchers, turning the fulfillment of this demand into a great challenge. There was a need to sensitize the actors involved in the institutional mission of Fiocruz and also to reflect on the role of S&T in our society, deconstructing a naturalized vision of science dissociated from the social.

To overcome the challenges posed by the BWEP, Fiocruz systematically followed its doctoral and postdoctoral research aligned with the program. The strategies focused on a greater integration of BWEP research, stimulation of the development of less explored topics, improvement of the monitoring and management tools of the Plan at Fiocruz, and stimulation of BWEP research to formulate solutions (products / processes / proposals) are needed. Finally, it began to offer support and guidance to PhD students and postdoctoral students in the development of research, guided by a critical discussion about the traditional model of scientific knowledge production.

An evaluation is never neutral, but it is anchored in a well-defined theoretical framework and considers its starting point and the goal to be achieved. The field of the Social Studies of Science and Technology supported the analysis developed, as well as subsidized the strategies created by the institution to integrate research and fulfill the objectives of the agreement. Thus, the discussions involving the relationships between science, technology and society were decisive in guiding the proposed actions/strategies, and they became incisive during the program alignment meetings and also during the discipline offered to BWEP students.

In this sense, the results of the experience of the Fiocruz-CAPES BWEP program, even if initial, lead us to think of alternatives to produce a science committed to social issues (not purely economic), interdisciplinary, that works together with society since the identification of their real demands, that offers sensitive responses to the local context and specificities of the population. These are real challenges facing the scientific community and should be prioritized and included in research agendas. It is hoped that this debate may inspire the creation of new socially engaged training programs and research that are sensitive to local contexts and that expand beyond the walls of knowledge production institutions.

Moreover, we believe that the study can contribute to the theoretical debate on the limits of the quantitative evaluation model, pointing out some possibilities of overcoming it. On the other hand, we do not believe that the alternative is to create another standard methodology/model to be followed universally, in any context or societies, and by different areas / disciplines. Simply because there is no single model of knowledge production, or even that S & T knowledge itself is not universal, but contingent, localized.

## Supporting information

S1 File Abstract book—BWEP Seminar. Abstract book of the Seminar "Graduate program in Fiocruz and the Brazil without Extreme Poverty Plan" of 2013 (Portuguese).
(PDF)

S2 File Report I. Reference document for the First BWEP meeting (Portuguese).
(PDF)

S3 File Report II. Analysis of the survey applied to post-doctoral students and the Brazil without Extreme Poverty Plan (Portuguese).
(PDF)
