# Supplementary material for: For socially engaged science: The dynamics of knowledge production in the Fiocruz graduate program in the framework of the "Brazil Without Extreme Poverty Plan"
Source: PLoS One. 2018 Oct 19;13(10):e0204232. doi: 10.1371/journal.pone.0204232 (PMC6195260; doi:10.1371/journal.pone.0204232)
Supplement: S1 File — Abstract book of the Seminar "Graduate program in Fiocruz and the Brazil without Extreme Poverty Plan" of 2013 (Portuguese). (PDF) [file pone.0204232.s001.pdf]

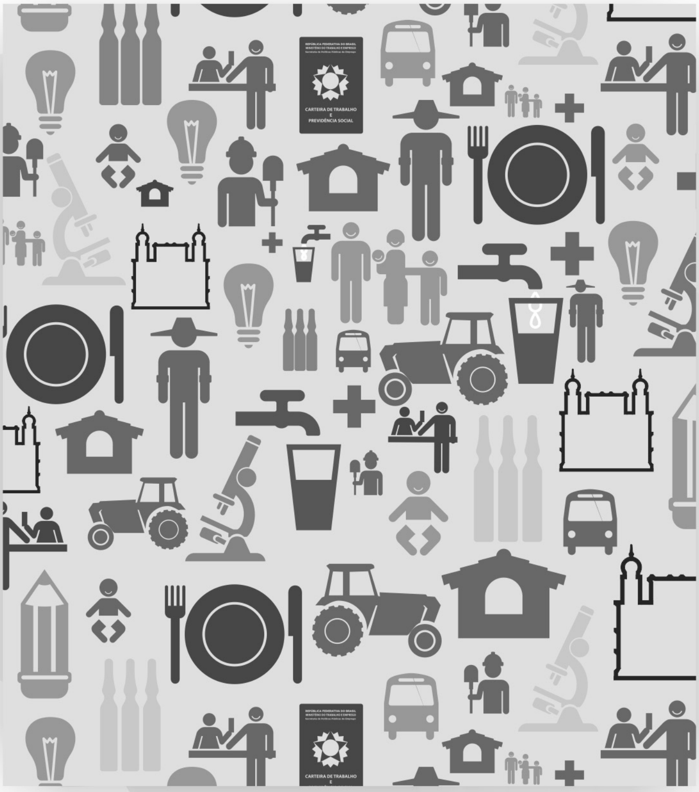

SEMINÁRIO

A Pós Graduação na Fiocruz e o Plano Brasil Sem Miséria

Caderno de Resumos

O Plano Brasil Sem Miséria (BSM), programa do Governo Federal, tem como objetivo elevar a renda e as condições de bem-estar da população extremamente pobre do país. O censo do IBGE de 2010 revela que 16,2 milhões de brasileiros ainda vivem com renda mensal abaixo de R$ 70,00. Através de busca ativa, o plano pretende identificar tais famílias e incluí-las em programas sociais para reduzir sua situação de vulnerabilidade.

Por entender que as populações em situação de pobreza extrema tem necessidades diferenciadas, o BSM tem ações nacionais e regionais, organizadas em três eixos: inclusão produtiva, garantia de renda, acesso a serviços.

Em Acordo de Cooperação Técnica firmado com o Ministério do Desenvolvimento Social e Combate à Fome, a Fiocruz compromete-se a desenvolver pesquisas voltadas para a geração de conhecimentos sobre problemas relacionados à extrema pobreza e articular tais conhecimentos com propostas de aplicação de tecnologias biomédicas, sociais e educacionais.

No âmbito da pós graduação, através de convênio com a CAPES/MEC, a Fiocruz recebeu 100 bolsas especiais de doutorado e 25 de pós-doutorado que vem sendo alocadas a projetos ligados às temáticas do Plano Brasil Sem Miséria, desde 2011. Até o momento, estão em desenvolvimento 50 teses de doutorado e 10 pesquisas de pós doutorado, em diversos programas de pós graduação e unidades da instituição.

As teses e pesquisas serão apresentadas no seminário **“A Pós Graduação na Fiocruz e o Plano Brasil Sem Miséria”** e estão disponíveis nesta publicação.

Paulo Gadelha
Presidente

Nisia Trindade Lima
Vice Presidente de Ensino, Informação e Comunicação

Fundação Oswaldo Cruz – Fiocruz

Presidente
Paulo Gadelha

Vice Presidente de Pesquisa e Laboratórios de Referência
Rodrigo Stabeli

Vice Presidente de Gestão e Desenvolvimento Institucional
Pedro Ribeiro Barbosa

Vice Presidente de Ensino, Informação e Comunicação
Nisia Trindade Lima

Vice Presidente de Ambiente, Atenção e Promoção da Saúde
Valcler Rangel Fernandes

Vice Presidente de Produção e Inovação em Saúde
Jorge Bermudez

Comissão organizadora

Coordenação Geral de Pós-graduação
Cristina Guilam

Coordenação Geral de Pós-graduação
Milton Moraes

Vice-Presidência de Ambiente, Atenção e Promoção da Saúde
Anna Cláudia Romano Pontes

Pós-doutoranda do Brasil Sem Miséria
Nadia Bomfim do Nascimento

Seminário
“A Pós Graduação na Fiocruz e o Plano Brasil Sem Miséria”

A Fundação Oswaldo Cruz, em conformidade com sua missão institucional e tradição de buscar aproximar ciência de qualidade à inovação e à promoção da saúde e da equidade social compromete-se com os desafios do Plano Brasil Sem Miséria (BSM), através de ações coordenadas pela Vice Presidência de Ensino, Informação e Pesquisa (VPEIC) e Vice Presidência de Ambiente, Atenção e Promoção da Saúde (VPAAPS).

No âmbito do ensino de Pós Graduação stricto sensu, a Fiocruz e a CAPES firmaram acordo de cooperação técnica e acadêmica, através do qual a agência de fomento destinou 100 bolsas de doutorado e 25 de pós-doutorado a alunos e pesquisadores dos programas da Fiocruz, cujas pesquisas estejam relacionadas aos eixos definidos pelo plano BSM. A implantação das bolsas vem ocorrendo a partir do segundo semestre de 2012, atendendo a critérios definidos pelo Comitê Gestor do BSM na Fiocruz e pelos programas de pós- graduação.

Portanto, neste evento temos o objetivo de promover o acompanhamento e integração dos projetos de doutorado e pós-doutorado, vinculados ao plano que serão expostos no formato de pôster ou comunicação oral.

Comissão Científica

Comissão Científica

Andrea Rodrigues Ávila
Instituto Carlos Chagas

Annibal Coelho de Amorim
Vice-Presidência de Ambiente, Atenção e Promoção da Saúde

Constancia Flavia Junqueira Ayres Lopes
Centro de Pesquisas Aggeu Magalhães

Cristina Guilam
Coordenação Geral de Pós-graduação

Helene Barbosa
Instituto Oswaldo Cruz

Joselia Oliveira Araújo Firmo
Centro de Pesquisas René Rachou

Marli Lima
Instituto Oswaldo Cruz

Milton Moraes
Coordenação Geral de Pós-graduação

Patricia Sampaio Tavares Veras
Centro de Pesquisas Gonçalo Muniz

Rosana Magalhães
Escola Nacional de Saúde Pública Sérgio Arouca

Tereza Maciel Lyra
Centro de Pesquisas Aggeu Magalhães

Virginia Torres Schall
Centro de Pesquisas Rene Rachou

Local do evento: Auditório do Museu da Vida
Data: 27 de agosto de 2013

Programação

8h às 9:00h – Café da manhã e Painel de Posters

9:00 h às 9:30 h – Mesa de Abertura
Paulo Gadelha- Presidente da Fiocruz;
Tereza Campello – Ministra do Desenvolvimento Social e
Jorge Guimarães – Presidente da Capes

9:30 às 11:30 h – Mesa redonda:
Política Social, Saúde e Cidadania: Impactos do Bolsa Família
Maurício Barreto – pesquisador do ISC/UFBA
Walquiria Leão – professora da Unicamp
Jeni Vaitsman – professora da ENSP/Fiocruz
Debatedor: Antonio Claret (SEASDH-Rio de Janeiro)

11:30h às 13h – Mesa 1
Moderador: Tania de Araújo Jorge (IOC);
Debatedor: Tereza Maciel (CPqAM/FIOCRUZ)
Mauricio Lisboa Nobre
Renata Pires Pesce
Taís Ferreira Gomes
Erick Araujo de Assumpção

13 às 15h – Almoço e Painel de Posters

15h às 16:30h – Mesa 2
Moderador: Rosana Magalhães
Debatedor: Virginia Schall
Sheila Soares de Assis
Daniele Blanco Cavalcanti
Fabio de Oliveira Fonseca
Flávia Stella Rego Furtado Cutrim
Marcus Vinicius Campos Matraca

17h – Encerramento

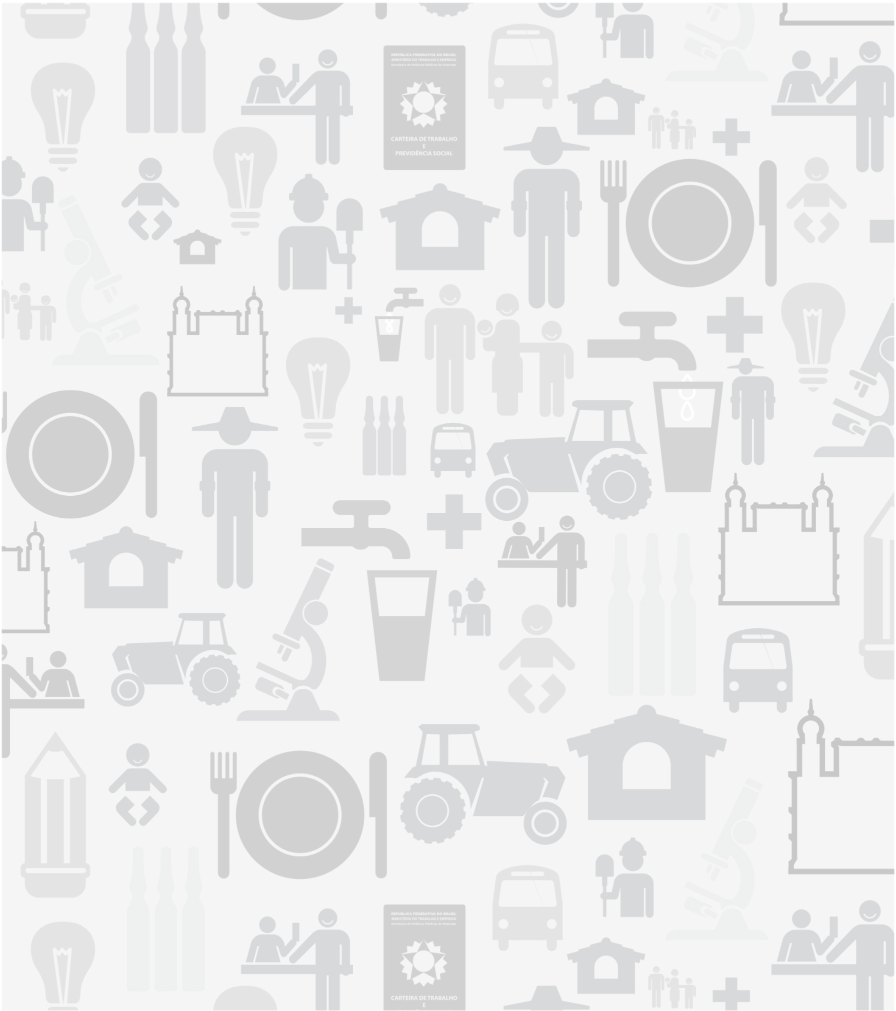

RESUMOS

Participante: **Alice Ricardo-Silva**

Título: **1. Ecologia de triatomíneos e ações de educação visando a prevenção da doença de Chagas na Amazônia**

Autores: Alice Ricardo-Silva, Catarina Lopes, Teresa Gonçalves

Filiação: Laboratório de Transmissores de Leishmanioses, IOC/ FIOCRUZ – RJ;

alice@ioc.fiocruz.br

Palavras-chave: *Triatoma maculata*, *Panstrongylus geniculatus*

Introdução: Dentre as doenças reconhecidas no Plano Brasil Sem Miséria como doenças negligenciadas está a doença de Chagas, afetando de 15 a 16 milhões de pessoas na América Latina. A Amazônia antes considerada indene está relacionada com uma condição emergente da doença. A proposta deste projeto é estudar no Estado de Roraima, no transecto noroeste/sul, o quadro ecológico das diferentes espécies que circulam na região e a investigação da domiciliação incipiente de algumas espécies vetoras como *Triatoma maculata* e *Panstrongylus geniculatus.* **Objetivos:** Identificar as populações de triatomíneos no Estado de Roraima, os ecótopos e possíveis hospedeiros envolvidos no ciclo de transmissão do parasito, verificar a sazonalidade desses vetores e eventuais processos de domiciliação. Buscando a integração de pesquisa e educação, serão ministradas capacitações para agentes de saúde, e professores das redes municipais e estaduais de ensino, permitindo que os esforços voltados para as medidas de vigilância e prevenção possam se concretizar de forma integral e contínua. Também será ministrado o "Curso de capacitação dos microscopistas de malária e dos laboratoristas da rede pública da detecção do *Trypanosoma cruzi*", que visa a detecção do parasito *T. cruzi* durante testes para o diagnóstico da malária, favorecendo o diagnóstico precoce da doença de Chagas.

Auxílio: CAPES/BSM, IOC

Participante: **Alexandre Pessoa Dias**

Título: **2. Tecnologias sociais em saneamento e saúde para o enfrentamento da transmissão de doenças de veiculação hídrica nos municipios de Madalena, Ceará e Rio Bonito do Iguaçu, Paraná**

Alexandre Pessoa Dias[1], Grácia Maria de Miranda Gondim[2], Antonio Henrique Almeida de Moraes Neto[3]

Filiação: [1]Laboratório de Educação Profissional em Vigilância em Saúde - LAVSA, Escola Politécnica da Saúde Joaquim Venâncio, EPSJV-FIOCRUZ, Rio de Janeiro

[2]Coordenação de Cooperação Social - CCI, Escola Politécnica da Saúde Joaquim Venâncio, EPSJV-FIOCRUZ, Rio de Janeiro

[3]Laboratório de Inovações em Terapias, Ensino e Bioprodutos - LITEB, Instituto Oswaldo Cruz- IOC, FIOCRUZ, Rio de Janeiro

apessoa@fiocruz.br

Palavras-chave: tecnologia social, saneamento, doenças de veiculação hídrica, Programa Água para Todos.

Nas comunidades vulneráveis, sob estresse hídrico, as tecnologias sociais se apresentam enquanto propostas de ação que contribuem no combate à pobreza extrema. A avaliação de desempenho socioambiental dessas tecnologias no campo da saúde justifica-se na melhor caracterização de seu alcance na promoção da saúde. Nosso objetivo é avaliar o estado da arte das tecnologias sociais em saneamento e saúde, com ênfase no manejo das águas, visando o enfrentamento da transmissão de doenças de veiculação hídrica e a redução das iniquidades socioambientais nos assentamentos da reforma agrária, contemplados pelo Curso Técnico em Meio Ambiente do Lavsa/EPSJV. A pesquisa é centrada na pesquisa-ação. Para análise de risco do manejo das águas, os aspectos qualiquantitativos em sua interação com a habitação, saneamento e produção alimentar serão diagnosticados através do mapeamento do ciclo antropogênico das águas, do monitoramento ambiental e da construção de protótipos de tecnologias sociais. Em atendimento ao Ministério da Saúde foi elaborado, com a Funasa, em versão preliminar, o Caderno Pedagógico

do Agente Comunitário de Saúde – *Cuidando da água de cisterna e da saúde ambiental no Semiárido*. Está sendo instalado o protótipo de fossa-verde em Jacarepaguá visando o monitoramento desse tratamento utilizado em Madalena. Os estudos dessas tecnologias darão subsídios para futuras expedições nos territórios contemplados.

Auxílio: IOC, EPSJV/FIOCRUZ

Participante: **André Luiz da Silva Lima**

Título: **3. O controle social no SUS: uma leitura em perspectiva histórica nas localidades de MANGUINHOS (1988/2010)**

Autores: André Luiz da Silva Lima

Filiação: Programa de Pós Graduação em História das Ciências e da Saúde- COC/ FIOCRUZ, Rio de Janeiro

Palavras-chave: História, Participação Social, SUS

O presente trabalho estrutura-se em apresentar considerações sobre um projeto de pesquisa – no nível de Doutorado - que estrutura-se para cumprir o objetivo de investigar, sob uma abordagem histórica, os espaços de participação social, institucionalizados pelo Sistema Único de Saúde entre os anos de 1988 e 2010, em particular nas localidades de Manguinhos. Para o desenvolvimento desta análise é importante considera: a) O SUS enquanto fruto de lutas políticas emergentes no contexto do movimento pela reforma sanitária e submetido a contextos históricos, sociais e econômicos distintos; b) A possibilidade de Controle Social (pela sociedade) ao SUS, de maneira plena, ainda é um processo em andamento; c) A historicidade da população de Manguinhos e a presença do narcotráfico e de práticas políticas clientelistas; d) A atuação da Fiocruz junto às localidades de Manguinhos; e) A possibilidade de rearranjos e reinterpretações do processo de participação social;

Auxílio: CAPES

Participante: **Ana Claudia Machado Duarte**

Título: 4. **Avaliação da Efetividade e Custo-Efetividade das Intervenções para Controle da Dengue em Municípios Da Região Metropolitana do Estado do Rio de Janeiro**

Autores: Ana Claudia Duarte, Marcio Neves Bóia[1].

Filiação: Laboratório de Doenças Parasitárias. Instituto Oswaldo Cruz-FIOCRUZ, Rio de Janeiro.

ana.claudia@bio.fiocruz.br

Palavras-chave: PNCD, dengue, custo-efetividade.

Introdução: A dengue constitui um grave problema de saúde pública no Brasil e no mundo, com grande incidência em áreas tropicais e uma clara associação com as condições precárias de infraestrutura habitacional, educativa e social, principalmente em áreas urbanas onde as condições de saneamento, o acesso à rede de distribuição de água e a coleta adequada de lixo não estão disponíveis de modo satisfatório. Para que um plano de controle da dengue se faça efetivo, deve considerar em suas intervenções, além das variáveis envolvidas na transmissão, que é necessário atingirem as populações em situação de extrema pobreza, levando a elas melhoria das condições de vida. **Métodos e resultados:** avaliaremos a efetividade e custo-efetividade das ações de intervenção propostas pelo Plano Brasil Sem Miséria e pelo Programa Nacional de Controle da Dengue em municípios da região metropolitana do estado do Rio de Janeiro por meio da comparação entre os resultados alcançados pelos municípios no período de 2011-2014 e uma situação epidemiológica hipotética de não-intervenção. **Conclusões:** pretende-se apresentar análise da situação da proposta nacional de controle da dengue e avaliação desta política pública, gerando dados que orientem o direcionamento adequado dos recursos financeiros, a fim de que estes investimentos resultem em ações efetivas no controle da doença.

Auxílio: FIOCRUZ/CAPES

Participante: **Andreia Silva de Souto**

Título: **5. As doenças infecciosas da pobreza e sua relação com ações do Plano Brasil sem Miséria no contexto das expedições da Fiocruz e da economia da inovação em Saúde.**

Autores: Andreia Silva de Souto[1], Tania Cremonini de Araujo-Jorge[1], Filipe Anibal Carvalho Costa[2],

Filiação: [1] LITEB - Laboratório de Inovações em Terapias, Ensino e Bioprodutos. Instituto Oswaldo Cruz - FIOCRUZ, Rio de Janeiro. [2] Laboratório de Sistemática Bioquímica. Instituto Oswaldo Cruz - FIOCRUZ.

asouto@ioc.fiocruz.br

Palavras-chave: Doenças Infecciosas da Pobreza, Plano Brasil sem Miséria, Expedições Fiocruz, Economia da Inovação em Saúde, e, Desenvolvimento Socioeconomico.

Introdução: As doenças ditas "negligenciadas" geram pobreza e intensificam os quadros de miséria no Brasil e em diversos países do mundo. O cidadão por elas acometido fica impedido de participar ativamente do desenvolvimento social e econômico, e fica prejudicada a mobilidade econômica e educacional de diversos grupos/comunidades/etnias ou regiões. Neste projeto pretendemos analisar a situação epidemiológica, socioambiental e econômica das comunidades alcançadas pelo Projeto IOC/FIOCRUZ "Expedições de Educação e Ciência para o Brasil sem Miséria", identificar os programas governamentais presentes, e as interlocuções estabelecidas pela Fiocruz nas expedições. Será feito o registro das intervenções realizadas pelos diversos atores sociais participantes antes, durante e depois das expedições e as necessidades de novas intervenções apontadas pelas próprias comunidades. Será realizado um mapeamento socioambiental destas comunidades para auxiliar o processo de futuras ações, e contribuir com a melhoria do panorama da saúde pública nas regiões atendidas pelas expedições Fiocruz pelo Brasil sem Miséria.

Auxílio: CAPES- Plano Brasil Sem Miséria

Participante: **Andressa A. Fuzari Rodrigues**

Título: **6. Brasil sem Miséria: Avaliação das Leishmnaioses em Comunidades Inseridas em Área de Preservação Ambiental, Parque Estadual da Serra da Tiririca, Niterói e Maricá, RJ, Brasil.**

Autores: Andressa A. Fuzari Rodrigues[1], Aline F. Santos Delmondes [1], Reginaldo P. Brazil [2]

Filiação: [1]Lab. Bioquímica e Fisiologia de Insetos – IOC – Fiocruz – Rio de Janeiro [2]. Lab. de Doenças Parasitárias – IOC – Fiocruz

andressafuzari@gmail.com; afuzari@ioc.fiocruz.br

Palavras-chaves: Flebomíneos, Leishmanioses, Rio de Janeiro

O Parque Estadual da Serra da Tiririca, localizado entre os municípios de Niterói e Maricá representa uma das poucas áreas com resíduos de mata atlântica próxima de área urbana, com ocorrência de casos humanos e caninos de leishmaniose tegumentar. As leishmanioses são doenças causadas por protozoários flagelados do gênero *Leishmania*, transmitidas pelos flebotomíneos, insetos pertencentes à ordem Diptera, família Psychodidae subfamília Phlebotominae. No Brasil, as leishmanioses tegumentar e visceral são doenças periurbanas onde a falta de saneamento e coleta de lixo esporádica ou inexistente favorece o aumento dos criadouros dos flebotomíneos aumentando o risco da doença. Este trabalho tem como objetivo avaliar aspectos epidemiológicos das leishmanioses e a vulnerabilidade das comunidades carentes a essa doença dentro da área do parque, através da pesquisa da fonte alimentar, infecção natural e avaliação dos animais sinantrópicos. Serão realizadas capturas mensais de flebotomíneos para identificação da fauna, detecção de infecção natural e avaliação da fonte alimentar; e capturas de pequenos mamíferos sinantrópicos para avaliação de infecção por Leishmania. As primeiras capturas foram iniciadas. Foi constatado a presença de flebotomíneos em todas as armadilhas utilizadas. Este material está sendo processado para posterior identificação.

Participante: **Beatriz Coronato Nunes**

Título: **7. Giardíase em populações rurais e ribeirinhas: Prevalência, impacto sobre o status nutricional e epidemiologia molecular.**

Autores: Beatriz Coronato Nunes[1], Aline C. Volotão[2], Filipe Anibal Carvalho-Costa[1].

Filiação: [1]Laboratório de Epidemiologia e Sistemática Molecular - IOC/ FIOCRUZ, [2]Universidade Federal Fluminense.

beatriz.nunes@ioc.fiocruz.br

Palavras-chave: *Giardia duodenalis*, status nutricional, epidemiologia molecular.

Introdução: A infecção por *Giardia duodenalis* é importante causa de doenças diarreicas em humanos, determinando síndrome disabsortiva e déficits nutricionais. A giardíase é, portanto, uma doença relacionada à pobreza e à urbanização sem infraestrutura. Este estudo visa identificar o impacto da infecção por *Giardia* no status nutricional de crianças, fatores epidemiológicos envolvidos na sua transmissão, os genótipos circulantes em humanos e animais e o potencial zoonótico de transmissão. **Métodos e resultados:** O desenho do estudo é transversal, com obtenção de dados coproparasitológicos (métodos de flutuação e sedimentação), socioeconômicos e antropométricos (altura, peso e perímetro braquial) de habitantes de Santa Isabel do Rio Negro-AM e de Cachoeiras de Macacu-RJ. Serão obtidas também amostras fecais de animais domésticos. Os escores de desvio-padrão dos parâmetros peso-idade, peso-altura e altura-idade serão calculados para as crianças (Módulo Nutrition EpiInfo/2000). As amostras de fezes positivas para *G. duodenalis* serão submetidas a métodos moleculares para genotipagem por sequenciamento. Estudo piloto revelou prevalência de 18,9% (62/328) em Santa Isabel do Rio Negro; 47 amostras foram submetidas à PCR para amplificação do gene [illegible]-giardina e, destas, 36 foram submetidas a sequenciamento genômico. Outros genes (tpi, SsrRNA, and gdh) estão sendo incluídos na análise filogenética. O estudo foi aprovado no CEP/FIOCRUZ/IOC (CAAE:121 25713.5.0000.5248/2013) e CEUA/FIOCRUZ(LW-21/13).

Auxílio: CAPES/FIOCRUZ/BSM.

Participante: **Biatriz Araújo Cardoso**

Título: **8. Proposição ao plano *"Brasil Sem Miséria"* de metodologia participativa para estudo de campo sobre a frequência de parasitoses intestinais e tuberculose infecção em área hiperendêmica com habitações precárias no Distrito do Murinin, Benevides, Pará**

Autores: Biatriz Araújo Cardoso[1,2,3], Antonio Henrique Almeida de Moraes Neto[2], Maria Helena Ferés Saad[3]

Filiação: [1]Universidade da Amazônia – UNAMA, Belém, Pará - PA
[2]Laboratório de Inovações em Terapias, Ensino e Bioprodutos – LITEB, Instituto Oswaldo Cruz – IOC, FIOCRUZ, RJ;
[3]Laboratório de Microbiologia Celular – LAMICEL, IOC, FIOCRUZ, RJ

biatriz.cardoso@ioc.fiocruz.br; saad@ioc.fiocruz.br

Palavras-chave: Habitações Precárias, Infecção latente por *M. tuberculosis* (LTBI), Parasitoses Intestinais, Tuberculose, Plano *Brasil Sem Miséria* (PBSM).

Os problemas ambientais e a transmissão das parasitoses intestinais (PI) e a tuberculose (TB) produzem um ciclo doença-pobreza-doença. Benevides apresenta 3980 famílias inscritas no Programa Bolsa Família e 52,27% da população vive abaixo da linha da pobreza. Ocorre prevalência elevada de TB infecção (77,88 casos/100.000 habitantes) e para a Secretaria Municipal de Saúde 100% apresenta infecção concomitante por parasitas intestinais. Tem-se por objetivo propor metodologia participativa para estudo de campo sobre a frequência de PI e TB infecção em área hiperendêmica com habitações precárias no Distrito do Murinin, Benevides, Pará, a fim de contribuir para a redução da prevalência em famílias alvo do Plano *Brasil Sem Miséria* (BSM). Dez moradias, com portadores de TB e outras dez controles serão avaliadas antes e após as melhorias sanitárias e arquitetônicas. Está em curso o cadastramento dos moradores. Serão aplicados questionários sobre o tema para subsidiar ações educativas, conjugadas com diagnóstico laboratorial e tratamento. Dos 40 moradores cadastrados, 10 são portadores de TB e 17 dos contatos estão latentemente infectados por *M. tuberculosis.* 82,5% estão parasitados. Será avaliado o impacto desta metodologia sobre o estado de saúde destes indivíduos, a

fim de apoiar o BSM, o SUS e fornecer subsídios para reforçar políticas públicas de saúde.

Auxílio: IOC, UNAMA, Prefeitura Municipal de Benevides – PA

Participante: **Camila Maria Oliveira de Azeredo**

Título: **9. Efeito *in vitro* de constituintes de óleos essenciais e derivados químicos sobre *Trypanosoma cruzi***

Autora: Camila Maria Oliveira de Azeredo e Maurilio José Soares

Filiação: ICC/Fiocruz-PR, Curitiba, PR

A doença de Chagas, causada pelo protozoário *Trypanosoma cruzi,* afeta milhões de pessoas no mundo. O tratamento baseia-se no uso de benzonidazol ou nifurtimox, ambos apresentando elevado custo, baixa eficácia na fase crônica e efeitos colaterais. Assim, a OMS incentiva estudos que apontem novas alternativas terapêuticas. Óleos essenciais são misturas complexas de metabólitos secundários de plantas, com poder bactericida, virucida e fungicida. Nossos resultados preliminares mostraram que os óleos essenciais de canela (*Cinnamomum zeylanicum*) e cabreúva (*Myrocarpus fastigiatus*) possuem atividade inibitória sobre *T. cruzi*, possivelmente devido à alta concentração de cinamaldeído e (E)-nerolidol, constituintes majoritários destes óleos, respectivamente. Neste projeto pretendemos analisar em mais detalhes o efeito de cinamaldeído e (E)-nerolidol, e derivados químicos deles, sobre o *T. cruzi*. Triagem em formas epimastigotas e avaliação da citotoxicidade sobre células Vero estão sendo feitas pelo método colorimétrico do MTT. Até o momento encontramos dois compostos candidatos a avaliações posteriores. Compostos com melhor atividade tripanocida serão avaliados posteriormente sobre formas tripomastigotas metacíclicas e amastigotas intracelulares do *T. cruzi*. Epimastigotas tratados com cinamaldeído, (E)-nerolidol e compostos selecionados após a triagem serão avaliadas por MET e MEV quanto à morfologia, por microscopia de fluorescência quanto ao ciclo celular, e por citometria de fluxo quanto ao potencial de

membrana mitocondrial, viabilidade celular, ciclo celular e indução de apoptose, na busca de possíveis mecanismos de ação.

Financiamento: CAPES, FIOCRUZ

Participante: **Camila Oliveira Antes**

Título: **10. Estudo do papel da proteína TcNRBD1 na tradução em *Trypanosoma cruzi***

Autora: Camila Oliveira Antes[1], Lysangela Ronalte Alves[1], Samuel Goldenberg[1]

Filiação: [1]Laboratório de Regulação e Expressão Gênica. Instituto Carlos Chagas- FIOCRUZ, Curitiba.

coliveira@tecpar.br

Palavras-chave: Proteínas de ligação ao RNA, *Trypanosoma cruzi*, regulação gênica.

A regulação da expressão gênica em tripanosomatídeos ocorre, principalmente, em nível pós-transcricional, mas poucas proteínas de ligação ao RNA foram caracterizadas até agora. O domínio RRM (*RNA Recognition Motif*) é um dos mais abundantes domínios encontrados em proteínas de ligação a RNA em eucariotos superiores. Este trabalho trata da caracterização funcional da proteína TcNRBD1, em *Trypanosoma cruzi*. Resultados prévios mostraram que TcNRBD1 é expressa durante todas as formas do ciclo de vida do parasita. A proteína apresenta um padrão granular mais concentrado na região perinuclear em todas as formas analisadas. O perfil de polissomos em gradiente de sacarose mostrou que TcNRBD1 está associada a polissomos. Além disso, o sequenciamento dos RNAs alvos de TcNRBD1 identificou vários RNAs não codificadores, especialmente snoRNAs e rRNAs. Foram sequenciados também mRNAs, dentre os quais os que codificam proteínas ribossomais foram os mais abundantes. A espectrometria de massas demonstrou que a proteína está associada a proteínas ribossomais, entre outras. Esses resultados indicam que TcNRBD1 tem papel na regulação da expressão

gênica estando associada a alguma etapa do processo de tradução em *T. cruzi*. Este projeto tem por objetivo então o estudo do papel da proteína TcNRBD1 em *T. cruzi* e em qual etapa da tradução ela está envolvida neste parasita.

Auxílio: CAPES, CNPq, FIOCRUZ.

Participante: **Carlos Eduardo Sampaio Guedes**

Título: **11. Avaliação de MTB2 como droga contra a leishmaniose cutânea e visceral**

Autores: Carlos Eduardo Sampaio Guedes[1], Beatriz Dias[1], Antonio Petersen[1], José Geraldo Lima[1], Gilberto Bomfim[2], Patrícia Veras[1]

Filiação: [1]Laboratório de Patologia e Biointervenção. CPQGM-FIOCRUZ, Bahia; [2] Instituto de Biologia - UFBA, Bahia

cesguedes@yahoo.com.br

Palavras-chave: *Leishmania*, Leishmaniose, Quimioterapia,

A leishmaniose é uma doença negligenciada que acomete, principalmente, a população pobre de países subdesenvolvidos e em desenvolvimento. Nos últimos anos houve aumento de casos no Brasil, associados à migração de populações para as zonas periféricas das cidades, onde condições econômicas precárias favorecem a proliferação do vetor e dos reservatórios da doença. O tratamento envolve o uso de drogas muito tóxicas, administradas por períodos longos e com efeitos colaterais severos, causando a desistência do tratamento e facilitando a emergência de cepas resistentes. Em trabalho realizado no LPBI, foi demonstrado que a molécula MTB2 inibe a proliferação de promastigotas de quatro espécies de *Leishmania* e o tratamento de macrófagos infectados por *L. amazonensis* causou redução da taxa de infecção, da carga parasitária e da viabilidade dos parasitas intracelulares em até 98%. Este trabalho tem por objetivo avaliar o potencial de MTB2 como droga para o controle da leishmaniose cutânea e visceral. Para este fim, será desenvolvida uma formulação

lipossomal de MTB2 que será utilizada para o tratamento, por trinta dias ininterruptos, de camundongos infectados por *L. amazonensis* e hamsters infectados por *L. infantum*. Ao fim do tratamento será avaliada a carga parasitária por PCR em tempo real e a histologia dos tecidos afetados.

Auxílio: CAPES, CPqGM-FIOCRUZ

Participante: **Caroline Ferraz Ignácio**

Título: **12. Análise do Processo da Gestão do Autocuidado em Relação às Parasitoses Intestinais: Uma Proposta de Dinamização e Humanização do Serviço no SUS**

Autores: Caroline Ferraz Ignacio[1], Antonio Henrique Almeida de Moraes Neto[1], Martha Macedo de Lima Barata[2]

Filiação: [1]Laboratório de Inovações em Terapias, Ensino e Bioprodutos. Instituto Oswaldo Cruz IOC, FIOCRUZ, Rio de Janeiro, RJ
[2]Assessoria de Planejamento Estratégico do IOC, Fiocruz, Rio de Janeiro

cfignacio@yahoo.com; caroline.ignacio@ioc.fiocruz.br

Palavras-chave: parasitoses intestinais, saúde da familia, gestão em saúde

As parasitoses intestinais (PI) são graves problemas de saúde pública e atuam como fatores de risco para outras doenças negligenciadas. Estas se retroalimentam conduzindo a um ciclo *doença-pobreza-doença*. Apesar de existirem instrumentos de política pública que visam combater a pobreza e lidar com os determinantes sociais da saúde, como o plano "*Brasil Sem Miséria*" (BSM), há lacunas na efetividade de sua implantação. Torna-se essencial examinar a interface dos instrumentos com os profissionais que o executam e com a população beneficiária. Esse projeto tem por objetivo analisar o processo de gestão do autocuidado relacionado às doenças negligenciadas da pobreza com foco nas PI, no Complexo de Manguinhos, RJ, a fim de contribuir para a humanização dos serviços no SUS, no âmbito do BSM. Dados qualiquantitativos serão obtidos através

de questionários aplicados aos profissionais da ESF da Clínica da Família Victor Valla e do Centro de Saúde Escola Germano Sinval Faria/ENSP/FIOCRUZ, e questionários e amostras de fezes obtidos por amostragem de conglomerados. Associações entre o conhecimento dos profissionais e a prevalência de parasitoses intestinais serão obtidas através de análise multivariada. Espera-se construir indicadores de processo em saúde relacionados ao enfrentamento das PI, seus fatores de risco e as co-morbidades para a promoção de saúde no SUS e a prevenção destes agravos em famílias alvo do BSM.

Auxílio: CAPES, IOC, CNPq

Participante: **Claudia Maria do Nascimento Moreira**

Título**: 13. Caracterização do Complexo Adaptador 1 em** *Trypanosoma cruzi*

Autores: Claudia Maria do Nascimento Moreira[1], Stenio Perdigão Fragoso[2], Maurílio José Soares[3], Rosana Elisa Gonçalves Gonçalves[4]

Filiação:[1]Laboratório de Biologia Molecular de Tripanossomatídeos. Instituto Carlos Chagas-FIOCRUZ, Curitiba-PR
[2]Laboratório de Biologia Celular. Instituto Carlos Chagas-FIOCRUZ, Curitiba-PR

cmoreira@tecpar.br

Palavras-chave: complexo adaptador 1, *Trypanosoma cruzi*.

O adaptador 1 (AP-1) é um complexo protéico heterotetramérico que auxilia na montagem de vesículas revestidas por clatrina na rede trans-Golgi (RTG), transportando enzimas lisossomais na via anterógrada e proteínas residentes da RTG na via retrógrada em eucariotos. Estudos sobre esse adaptador são escassos em tripanossomatídeos. Porém, demonstrou-se que a deleção das subunidades µ1 e [illegible]1 pode afetar a infectividade de *Leishmania mexicana* em macrófagos e camundongos. Além disso, o silenciamento do gene que codifica a subunidade µ1 em *Trypanosoma brucei* mostrou que essa proteína é essencial ao parasita,

atuando na seleção de proteínas lisossomais na RTG. Recentemente, demonstrou-se a localização subcelular da subunidade AP1-▯ no complexo de Golgi em *T. cruzi*. Estudos sobre o comportamento desse adaptador em *T. cruzi* podem auxiliar na identificação de possíveis alvos quimioterápicos para o tratamento da doença de Chagas, que afeta milhões de brasileiros. Assim, esse projeto tem como objetivos: estudar a função do AP-1 através de nocaute gênico e dominante negativo; avaliar a importância desse complexo em processos como divisão celular, diferenciação, endocitose, infectividade e transporte de proteínas para os reservossomos em *T. cruzi* além de identificar os complexos protéicos associados à subunidade AP1- através de ensaios de interação.

Auxílio: CAPES

Participante: **Cleiton Silva Santos**

Título: **14. O papel do óxido nítrico na patogênese da leptospirose experimental em hamsters e camundongos**

Autores: Cleiton Silva Santos[1], Everton Cruz de Azevedo[1,2], Luciane Marieta Soares[1,2], Magda Oliveira Seixas Carvalho[1,2], Andréia Carvalho dos Santos[1,2], Adenizar Delgado das Chagas Júnior[1], Caroline Luane Rabelo da Silva[1], Ursula Maira Russo Chagas[1], Mitermayer Galvão dos Reis[1], Daniel Abensur Athanazio[1,2]

Filiação: [1] Laboratório de Patologia e Biologia Molecular (LPBM), Centro de Pesquisas Gonçalo Moniz (CPQGM), Fiocruz, Salvador, Bahia, Brasil. [2] Universidade Federal da Bahia, Salvador, Bahia, Brasil.

csantos@conveniado.bahia.fiocruz.br

Palavras-chave: Leptospirose, azul de metileno, modelo, animal

Introdução: A leptospirose em humanos usualmente envolve hipocalemia e hipomagnesemia. Estes desequilíbrios iônicos podem estar relacionados com a produção renal do óxido Nítrico (NO). Nós demonstramos previamente a correlação entre os níveis séricos de NO

e a gravidade da doença renal em pacientes com leptospirose grave. Azul de metileno (AM) inibe a guanilato ciclase solúvel (ação à jusante da NO sintase) e recentemente foi relatado seu efeito benéfico sobre a sepse clínica e experimental. **Métodos.** Investigamos a ocorrência de alterações iônicas séricas e o desfecho clínico da leptospirose experimental em vários intervalos de tempo (4, 8, 16 e 28 dias) em modelo hamster com grupos não tratados e tratados com ampicilina, AM ou ambos. **Resultados.** Hipocalemia e hipomagnesemia não foram reproduzidas neste modelo. O tratamento com AM não mostrou efeito sobre o desfecho clínico. **Conclusões:** O tratamento adjuvante com AM não demonstrou efeito benéfico quando combinado à antibioticoterapia no modelo de leptospirose experimental em hamsters.

Auxílio: CAPES.

Participante: **Cristina Xavier de Almeida Borges**

Título: **15. Resíduos sólidos e a frequência das helmintoses de caráter zoonótico no complexo de Manguinhos, RJ: contribuições em saúde para o "Plano Brasil Sem Miséria"**

Autores: Cristina Xavier de Almeida Borges[1], Antonio Henrique Almeida de Moraes Neto[1], Filipe Anibal Carvalho Costa[2]

Filiação: [1]Laboratório de Inovações, Terapias, Ensino e Bioprodutos - LITEB Instituto Oswaldo Cruz - IOC, Fiocruz, Rio de Janeiro;
[2]Laboratório de Sistemática Bioquímica, IOC, FIOCRUZ, Rio de Janeiro

crisborges607@gmail.com; cristina.borges@ioc.fiocruz.br

Palavras-chave: *Larva Migrans*; Resíduos Sólidos

Problemas em favelas remontam à sua formação e associam-se à degradação ambiental. A dispersão de resíduos sólidos atrai cães e gatos que são reservatórios de infecções, principalmente as causadas por *Ancylostoma caninum e Toxocara canis*, que produzem acidentalmente no homem síndromes zoonóticas erráticas por *larva migrans*. Objetiva-se investigar a possível relação entre o manejo de resíduos sólidos e a

frequência de *larva migrans* causada por *A. caninum e T. canis*, em famílias alvo do "*Plano Brasil Sem Miséria*" (BSM), no Complexo de Manguinhos, RJ. Para a pesquisa utilizam-se questionários sobre posse responsável de animais, manejo de lixo e conhecimentos sobre zoonoses em 63 domicílios. Uma busca ativa de dermatoses caraterizadas como *larva migrans* será realizada com as agentes comunitárias de saúde, em desenho transversal. A prevalência destas infecções será estimada e apresentada estratificada por faixa etária, localidade e sexo. Fatores associados serão identificados. Estes dados estão sendo analisados nos Softwares Epi-Info 3.5.1 e SPSS 15.0. Dos entrevistados (75%) observaram animais errantes, (3%) relacionaram as zoonoses à transmissão por animais, 50% têm animais domésticos, 16% são vermifugados, 87% desconhecem *larva migrans,* Estabeleceu-se parcerias com as Unidades de Saúde e Superintendência de Vigilância e Fiscalização Sanitária em Zoonoses, RJ, para estratégias de promoção à saúde. Espera-se propor recomendações para o enfrentamento desses agravos.

Auxílio: CAPES, IOC/FIOCRUZ

Participante: **Daniela de Pita Pereira**

Título**: 16. Aplicação de Ensaios Moleculares para o Estudo das Leishmanioses em Áreas Endêmicas no Brasil.**

Autores: Daniela de Pita Pereira[1], Andressa Fuzzari[2], Thaís de Araújo Pereira[1] Taiana Amancio[1] Constança Britto[1] Reginaldo Brazil[2]

Filiação: [1] Laboratório de Biologia Molecular e Doenças Endêmicas. Instituto Oswaldo Cruz - FIOCRUZ, Rio de Janeiro
[2] Laboratório de Doenças Parasitárias. Instituto Oswaldo Cruz - FIOCRUZ, Rio de Janeiro

danypita@ioc.fiocruz.br; danypyta@gmail.com

Palavras-chave: Leishmaniose, flebótomo, diagnóstico molecular

Dentro do Projeto Brasil sem Miséria, proposto pelo Governo Federal,

estaremos avaliando 3 áreas: os municípios de Brasiléia e Rio Branco, no Estado do Acre, que fazem parte da Amazônia brasileira, onde foram notificados em 2010, 40% dos casos de LT do Brasil e no Estado do Rio de Janeiro no Parque Estadual da Serra da Tiririca, região oceânica que vem sofrendo um processo de impacto ambiental intenso devido a ocupação humana próximo às encostas da Serra, com a ocorrência esporádica de casos humanos e caninos de Leishmaniose Tegumentar. Serão utilizados diferentes ensaios moleculares para a pesquisa da fonte alimentar e infecção natural dos flebotomíneos coletados nas três áreas de estudo, que permitirão avaliar os aspectos epidemiológicos das leishmanioses nos municípios estudados. Sabendo que a pobreza é um dos fatores de riscos para a leishmaniose e que as regiões que serão avaliadas apresentam concentração de populações de baixo poder aquisitivo, uma análise real desse quadro é necessário na avaliação dessas áreas para uma proposta política em saúde na prevenção e controle dessa doença.

Auxílio: CAPES, IOC, CNPq , FAPERJ

Participante: **Daniele Blanco Cavalcanti**

Título**: 17. A cultura da sustentabilidade no contexto do Programa Mais Educação**

Autores: Daniele Blanco Cavalcanti[1], Marco Antônio Costa

Filiação: [1] Instituto Oswaldo Cruz- FIOCRUZ, Rio de Janeiro
[2] Instituto Oswaldo Cruz- FIOCRUZ, Rio de Janeiro

daniele.cavalcanti@ioc.br; costa@fiocruz.br

Palavras-chave: Ensino de Ciências, Educação Ambiental e Brasil Sem miséria.

O Programa Mais Educação integra o Plano Brasil sem Miséria desde 2011, visando propagar a Educação Integral através de oportunidades variadas de ensino, dentre elas, atividades abordando Educação Ambiental (EA). Enfocamos nesta pesquisa principalmente a Comissão de Meio Ambiente e Qualidade de Vida (Com-vida) na Escola e a Horta escolar.

A Com-Vida surge para estabelecer um espaço de reflexão sobre nossas ações diante do meio ambiente, reunindo diferentes atores sociais para discutir e buscar possíveis soluções para as questões socioambientais. Em relação à Horta Escolar, pretendemos analisar a existência ou não de atividades interdisciplinares dentro deste contexto. Intentamos traçar um perfil das atividades ambientais realizadas em duas escolas, tendo como norte a cultura da sustentabilidade, visando à diagnose da corrente de EA propagada nestas, e a determinação da relação entre as atividades desenvolvidas no âmbito do Programa Mais Educação e as descritas no Projeto Político Pedagógico. A sustentabilidade passa a considerar um conjunto de atores sociais por meio de práticas educativas, que visam reforçar o sentimento de responsabilidade, a capacidade de refletir e tomar decisões relativas aos problemas socioambientais. Para o atingimento dos objetivos propostos, realizaremos uma pesquisa documental, caracterizada como um estudo de casos múltiplos, com abordagem qualitativa, e como instrumentos de coleta de dados, utilizaremos, entrevistas semiestruturadas e grupo focal.

Auxílio: CAPES, IOC, CNPq

Participante: **Danielle Barros Silva Fortuna**

Título: **18. Oficina de história em quadrinhos (HQ) no ensino de Ciências: Produção de HQ em uma escola no Acre**

Autores: Danielle Barros S. Fortuna[1], Paulo Roberto Vasconcellos[2], Tânia Araújo-Jorge[3]

Filiação: [1,2,3] LITEB/IOC/Fiocruz, Rio de Janeiro

danbiologa@gmail.com

Palavras-chave: Ensino de Biociências e Saúde, Oficina de História em Quadrinhos, Expedições Científicas.

Resumo: O Plano Brasil Sem Miséria (PBSM) foi implantado objetivando elevar renda e minorar desigualdade socioeconômica no Brasil. Estratégias tem sido utilizadas como acesso a serviços públicos nas áreas

de educação, saúde, assistência social, etc. Para atender os desafios do PBSM, o Instituto Oswaldo Cruz vem realizando expedições de 2012 a 2014 por localidades de extrema pobreza. Ensino, saúde e cultura são elementos essenciais para superação da pobreza, por isso as expedições são ancoradas em ações de formação continuada de professores da educação básica. Em setembro de 2012 houve expedição em Rio Branco e dentre as atividades foram desenvolvidas oficinas de HQ com alunos e professores de escola pública como parte de uma pesquisa de doutorado. Objetivo: Compartilhar tópicos sobre linguagem, processo criativo e possibilidades de utilização pedagógica com a criação de HQ pelos alunos. Considerações: Notou-se grande interesse por parte das turmas, contudo detectamos dificuldades na produção dos roteiros (escrita; noção de início, meio e fim; e interpretação). Ao final da produção, os alunos apresentaram a HQ construída pelo grupo, momento interessante de integração da turma. Professores tiveram interesse em perpetuar a proposta. Espera-se que a pesquisa contribua para o fortalecimento da utilização das HQ no ensino pelo Brasil.

Auxílio: CAPES/PBSM

Participante: **Danielle Misael de Sousa**

Título: **19. Eco-epidemiologia de triatomíneos e tripanosomas e o ciclo de transmissão da Doença de Chagas associados a ações de educação em saúde no estado do Ceará em consonância com o Plano Brasil Sem Miséria (bsm).**

Autores: Danielle Misael, Jacenir Mallet

Filiação: Laboratório de Transmissores de Leishmanioses – Setor de Entomologia Médica e Forense. Instituto Oswaldo Cruz - FIOCRUZ, Rio de Janeiro.

misael@ioc.fiocruz.br

Palavras-chave: *Trypanosoma cruzi*;

Introdução: A doença de Chagas, segundo a OMS e OPAS, pertence ao grupo das "doenças infecciosas da pobreza", e para a mitigação de problemas relacionados à pobreza, foi incluída como um dos eixos de Ações do Plano Brasil sem Miséria (BSM) do Governo Federal. Este projeto será realizado na região do Cariri, Ceará, área de antiga colonização e ocupação onde o risco de transmissão da doença de Chagas persiste por fatores, como baixas condições de vida da população e tem como objetivo geral realizar um estudo eco-epidemiológico de populações de triatomíneos e sua relação com o ciclo de transmissão da doença de Chagas, além de realizar ações de educação em saúde como ação concreta de apoio ao BSM numa interação com a comunidade, professores e profissionais de saúde. **Métodos:** Levantamento da fauna triatomínica e identificação da fonte alimentar; Pesquisa de *Trypanosoma cruzi* nos animais domésticos e triatomíneos; Realização de inquérito sorológico na população dos municípios; Geração de produtos educacionais com relação à biologia dos triatomíneos (profissionais de saúde, educação e comunidade). **Resultados**: Primeira viagem à região com definição de parceria com a 20ª Célula Regional de Saúde do Ceará.

Participante: **Davide Rasella**

Título: **20. Impacto do Plano Brasil sem Miséria e do Programa Bolsa Família sobre a mortalidade materna e neonatal no Brasil**

Autores: Davide Rasella[1,2,3], Gerson Oliveira Penna[1,4], Mauricio Lima Barreto[2,3]

Filiação: [1] Escola de Governo da FIOCRUZ, Brasília, [2] Instituto de Saúde Coletiva/UFBA, [3]INCT-CITECS-Ciência, Tecnologia e Inovação em Saúde, [4]Núcleo de Medicina Tropical/UNB

davide.rasella@gmail.com

Palavras-chave: Plano Brasil Sem Miséria, Programa Bolsa Família, Mortalidade neonatal, Mortalidade materna.

Introdução: Nos últimos anos a mortalidade materna e neonatal no Brasil

tiveram um decréscimo, mas ainda permanecem elevadas sobretudo nas faixas mais pobres da população. Esta redução foi devidas - entre outros - à implementação de novas políticas sociais e de saúde no país. Entre as políticas de redução da pobreza que tiveram a maior expansão na ultima década há o Programa Bolsa Família (PBF) e recentemente o Plano Brasil Sem Miséria (PBSM) que, alem de aumentar os benefícios pelas famílias extremamente pobres já assistidas pelo PBF, procura integrar estas ajudas com programas de acesso aos serviços públicos em diferentes áreas. O objetivo deste projeto será avaliar o efeito do PBF e do PBSM, juntamente com o Programa Saúde da Família, na redução da mortalidade materna e neonatal nos municípios brasileiros. **Métodos e resultados:** Será utilizado um desenho de estudo ecológico misto, sendo a unidade de analise os municípios brasileiros no período de 2004 até 2011. Serão estimadas as medidas de efeito por meio da regressão negativa binomial multivariada por dados em painel. Dos 5565 municípios brasileiros, serão selecionados os com qualidade de informações vitais considerada adequada segundo critérios estabelecidos. **Conclusões:** Espera-se obter estimativas do impacto do PBF e do PBSM que possam ajudar na implementação ou reformulação das mesmas políticas publicas ou de políticas publicas relacionadas.

Auxílio: Iniciativa Fundação Oswaldo Cruz e Capes Brasil Sem Miséria, INCT-CITECS-Ciência, Tecnologia e Inovação em Saúde

---

Participante: **Denise Andréa Silva de Souza**

Título**: 21. Aprimoramento do sistema de nocautes múltiplos em *Trypanosoma cruzi* e sua aplicação na caracterização de genes candidatos com relevância funcional**

Autores: Denise Andréa Silva de Souza[1,2], Christian Macagnan Probst[1,2],

Filiação: [1]Laboratório de Genômica Funcional, Instituto Carlos Chagas-FIOCRUZ, Paraná

[2]Laboratório de Bioinformática e Biologia Computacional, Instituto Carlos Chagas-FIOCRUZ, Paraná

denise_andreass@yahoo.com.br; cprobst@fiocruz.br

Palavras-chave: doença de Chagas, *Trypanosoma cruzi*, caracterização funcional, nocaute gênico, automatização.

Introdução: a doença de Chagas é uma doença endêmica de populações rurais e possui uma história de negligência à sua prevenção, diagnóstico e tratamento. A atual atenção governamental de assistência à população carente traz novas expectativas quanto à prevenção, tratamento e acompanhamento de doenças como a doença de Chagas - associadas à pobreza-, tornando fundamental o estudo das bases biológicas do agente etiológico, o *Trypanosoma cruzi*, uma vez que a maioria dos cerca de 9 mil genes codificadores de proteínas é classificada como de função desconhecida/putativa. Neste sentido, nosso grupo desenvolve estudos de caracterização funcional de proteínas de *T. cruzi*, dentre os quais se destaca este projeto de desenvolvimento de um sistema automatizado de nocautes gênicos em *T. cruzi*. **Métodos e resultados:** implementação de um sistema automatizado de construção de cassetes de nocaute e sua utilização para nocaute gênico de *T. cruzi*. **Conclusões:** atualmente, o sistema consiste em:

- *Software* para predição de *primers* para construção de cassetes de nocaute;
- Conjunto de vetores para remoção da região codificadora alvo (contendo genes de resistência à antibióticos ou genes de proteínas fluorescentes);
- Protocolo inicial de alta eficiência baseado em PCR de fusão para a criação dos cassetes de nocaute.

Com a estrutura do sistema montada, a necessidade atual é o aprimoramento das rotinas, especialmente visando à automatização.

Auxílio: CAPES, ICC, CNPq.

Participante: **Erick Araujo de Assumpção**

Título: **22. Bioética e Habitação: reflexões sobre o papel do Estado nos conflitos urbanos**

Autor: Erick Araujo de Assumpção

Filiação: Programa de Pós-graduação em Bioética, Ética Aplicada e Saúde Coletiva (PPGBIOS), Escola Nacional de Saúde Pública (ESNP)/ Universidade do Rio de Janeiro (UERJ)/ Universidade Federal Fluminense (UFF)/ Universidade Federal do Rio de Janeiro (UFRJ), Rio de Janeiro, Brasil

ericklaraujo@gmail.com

Palavras-chave: Bioética; Habitação; Estado.

Introdução: A presente pesquisa tem com objetivo geral analisar o tratamento no campo da bioética dos conflitos urbanos. Para tanto, apresenta-se como necessária a análise de políticas estatais que visam extinguir a pobreza e suas consequências no Brasil, nominalmente o programa Brasil sem Miséria e respectivos programas atuantes na questão habitacional, assim como nos serviços prestados à população em situação de rua. Em uma perspectiva não dicotômica de análise, demonstra-se pungente verificar os papéis que o Estado pode assumir, desde agente de vulneração a um possível agente de bem estar, necessariamente em dialogo com as necessidades dos indivíduos. **Métodos:** Coleta de dados qualitativos por meio de etnografia de grupos atingidos por problemas relativos ao déficit habitacional e atendidos por políticas públicas, a saber: população em situação de rua e habitantes de ocupações urbanas. **Conclusões:** Infere-se que o Estado ainda apresenta propensão à defesa da propriedade privada – representada pela existência de imóveis abandonados, quando o número destes é superior ao déficit habitacional em detrimento a políticas de reforma urbana.

Auxílio: CAPES, IOC, CNPq

Participante: **Érika Silva do Nascimento Carvalho**

Título: **23. Desenvolvimento de estratégias de ação aplicáveis aos programas de monitoramento e controle de simulídeos (insecta: diptera)**

Autores: Érika Silva do Nascimento Carvalho[1], Daniel Forsin Buss[2], Marilza Maia Herzog[1]

Filiação: [1]Laboratório de Simulídeo e Oncocercose. Instituto Oswaldo Cruz – FIOCRUZ, Rio de Janeiro. [2]Laboratório de Avaliação e Promoção da Saúde Ambiental. Instituto Oswaldo Cruz – FIOCRUZ, Rio de Janeiro.

erikar@ioc.fiocruz.br

Palavras-chave: Simulídeos, Planejamento, Controle

Introdução: Os simulídeos são insetos que estão diretamente relacionados às populações ribeirinhas e rurais. As fêmeas apresentam hábito alimentar antropofílico e/ou zoofílico, podendo causar não só reações imunológicas severas, mas também a transmissão de agentes patogênicos. Apesar de existirem vários programas de controle de simulídeos no Brasil, majoritariamente eles se baseiam unicamente no uso de bioinseticida, o que apresenta alto custo, sendo um dos fatores limitantes a continuidade dos programas. Visto isso, o controle de simulídeos continua sendo um desafio para o País, pois sua efetivação ocorre sem considerar a participação social e aspectos socioambientais, salvo exceções. Então, faz-se necessário e apropriado o desenvolvimento de medidas que norteiam o planejamento das ações de controle, proporcionando autonomia aos municípios. Principalmente que favoreça a população rural ribeirinha no que tange a melhorias das condições de saúde, do modo de vida e do sistema de produção. Dentro deste contexto, este projeto objetiva desenvolver um instrumento normativo com instruções para planejamento estratégico aplicável às atividades de controle de simulídeos na instância pública, com participação social, bem como organizar um modelo de capacitação técnica para viabilizar a operacionalização dos trabalhos de campo no controle de simulídeos.

Auxílio: LSO/IOC

Participante: **Fabio de Oliveira Fonseca**

Título: **24. Perfil de contaminação sanitária em habitações precárias no Distrito de Murinin em Benevides – Pará: proposta de melhorias em saneamento no âmbito do plano “*Brasil Sem Miséria*”**

Autores: Fabio de Oliveira Fonseca[1,3], Filipe Anibal Carvalho Costa[2], Antonio Henrique Almeida de Moraes Neto[3]

Filiação: [1] Universidade da Amazônia – UNAMA, Belém, PA
[2] Laboratório de Sistemática Bioquímica, Instituto Oswaldo Cruz, IOC, FIOCRUZ, RJ
[3] Laboratório de Inovações em Terapias, Ensino e Bioprodutos – LITEB, IOC, FIOCRUZ, RJ

fabioofonseca@gmail.com; ahmn@ioc.fiocruz.br

Palavras-chave: Parasitoses intestinais, Tecnologias Sociais, Saneamento, Plano *Brasil Sem Miséria*

O plano *"Brasil Sem Miséria"* (BSM) destaca que 53% dos domicílios em extrema pobreza não possuem ligação com redes de esgotamento séptico e pluvial. A transmissão de parasitoses intestinais nestas áreas está principalmente associada a dificuldades de acesso a saneamento básico, atendimento médico e informações sobre medidas profiláticas. De acordo com o levantamento cadastral efetuado no Distrito de Murinin, em famílias alvo do BSM, a totalidade das habitações possui instalações empíricas e inadequadas de esgotamento sanitário. Este estudo tem por objetivo determinar o perfil de contaminação sanitária em habitações precárias no distrito de Murinin, Benevides, Pará e apresentar propostas de melhoria em saneamento, no âmbito do BSM. Nossos resultados preliminares, por exames coproparasitológicos pelo método de Lutz 1919 (n=40) e com apoio da Secretaria Municipal de Saúde, demonstraram que 82,5% dos moradores (n=33) estão parasitados. Os parasitas mais frequentes foram *Endolimax nana* (40%), *Entamoeba coli* (40%), *Giardia lamblia* (17,5%), *Entamoeba histolytica* (10%) e *Iodameba butschlii* (10%). Estes indivíduos estão sendo tratados sob supervisão médica e incluídos em oficinas que estimulam a construção de tecnologias sociais (TS) em saneamento. Pretende-se apoiar o BSM e o Sistema Único de Saúde, através da construção de TS em saneamento como contraponto à contaminação ambiental para o controle da transmissão de parasitoses intestinais neste território.

Auxílio: IOC, UNAMA, Prefeitura de Benevides – PA

Participante: **Fernanda Loureiro de Moura**

Título: **25. Ocorrência de toxoplasmose congênita, avaliação do conhecimento sobre toxoplasmose e do acompanhamento sorológico das gestantes e implantação de medidas de prevenção primária nos programas de pré-natal da Rede Pública de Saúde do município de Niterói-RJ.**

Autores: Fernanda Loureiro de Moura[1], Patricia Riddell Millar Goulart[1,2], Maria Regina Reis Amendoeira[1].

Filiação:[1] Laboratório de Toxoplasmose. Instituto Oswaldo Cruz. FIOCRUZ, Rio de Janeiro.
[2] Departamento de Microbiologia e Parasitologia. Universidade Federal Fluminense.

fernanda.loureiro@ioc.fiocruz.br

Palavras-Chaves: Conhecimento; *Toxoplasma gondii;* Pré-natal.

Introdução: Este estudo objetivou avaliar o conhecimento sobre as formas de transmissão e prevenção da toxoplasmose. **Métodos e Resultados parciais:** Estão participando 46 profissionais de saúde e 38 gestantes dos módulos do Programa Médico de Família de Niterói-RJ, desde abril de 2013. Todos assinaram o TCLE, responderam um questionário, receberam material informativo e orientações sobre a protozoose. Das 38 gestantes, 14 (36,8%) conheciam a toxoplasmose. Destas, 42,8% obtiveram informações pela televisão e 28,6% pelo médico. 57,1% desconheciam as formas de prevenção. Dos 46 profissionais, 10 (21,7%) agentes comunitários de saúde não conheciam a toxoplasmose. Foram relatados formas corretas de transmissão: 94,4% fezes de gatos, 52,7% consumo de carnes cruas, 25% verduras, frutas e legumes crus mal lavados, 61,1% forma transplacentária, 19,4% consumo de leite cru, como também formas erradas: 19,4% peixe cru, 22,2% fezes de pombos e 27,8% fezes de cães. O encaminhamento da gestante infectada para o serviço de referência foi a medida preventiva mais citada (83,3%), seguida da lavagem adequada das mãos (77,7%), uso de luvas ao manusear terra (66,6%) e não consumir carne crua (47,2%). **Conclusão**: a falta

de conhecimento sobre a toxoplasmose é elevada, sendo importante a implantação das medidas de prevenção primária no pré-natal.

Auxílio: CAPES, IOC, CNPq.

Participante: **Flávia Stella Rego Furtado Cutrim**

Título**: 26. Estudo dos casos agudos de Doença de Chagas no Maranhão, Brasil, e sua relação com a pobreza.**

Autores: Flávia Stella Rego Furtado Cutrim[1], Angela Cristina Veríssimo Junqueira[1], Eloisa da Graça do Rosário Gonçalves[2]

Filiação: [1] Laboratório de Doenças Parasitárias, Instituto Oswaldo Cruz – FIOCRUZ, Rio de Janeiro. [2]Centro de Referência em Doenças Infecciosas e Parasitárias, Universidade Federal do Maranhão.

flavia.furtado@ioc.fiocruz.br.

Palavras-chave: Doença de Chagas Aguda; Maranhão; Pobreza.

Introdução: A Doença de Chagas, no Maranhão, Brasil é abordada enfocando casos agudos notificados de 1975 a 2015. Será delineado o perfil clínico-epidemiológico e indicadas medidas de atenção ao paciente, bem como, proposta ações de prevenção, vigilância e controle nos municípios de autoctonia. Serão correlacionadas as condições socioeconômicas com a ocorrência de casos, verificando se a morbidade retroalimenta o ciclo pobreza-doença-pobreza, sendo o estado o penúltimo no Índice de Desenvolvimento Humano. **Métodos e resultados:** o estudo é descritivo e exploratório, retrospectivo e prospectivo, com obtenção de informações a partir das fichas de investigação do SINAN e de prontuários, coleta nos municípios acometidos com questionários focais. Resultados preliminares mostram 63 casos agudos, de 1975 a 2012, procedentes de 28 municípios. O treinamento dos microscopistas revisores iniciou em junho de 2013 e como multiplicador, na Regional de Açailândia com mais 9 municípios. Paralelamente, ações educativas com as populações das áreas acometidas e elaboração de informes técnicos, cartilhas e manuais.

A reavaliação clínica dos casos retrospectivos iniciará em agosto de 2013. Conclusões: A necessidade de capacitação técnica em mais 190 municípios, após realizar com outros 27.

Auxílio: CAPES, IOC, FAPEMA.

Participante: **Geane Lopes Flores**

Título: **27. Desenvolvimento de metodologia para diagnóstico e avaliação imunológica da co-infecção HIV-hepatites B e c aplicáveis a grupos vulneráveis, incluindo as populações alvo do Plano Brasil Sem Miséria.**

Autores: Geane Lopes Flores1, Brunna Lemos Crespo Marques1, Marjorie Parra de Lima1, Juliana Custodio Miguel1, Elisangela Ferreira da Silva1, Jaqueline Correia de Oliveira1, Denise Ferreira Vigo Potsch2, Lia Laura Lewis-Ximenez1, Elisabeth Lampe1, Livia Melo Villar1.

Filiação: 1- Laboratório de Hepatites Virais, Instituto Oswaldo Cruz, Fiocruz – Rio de Janeiro
2- Hospital Clementino Fraga Filho – UFRJ- Rio de Janeiro

geaneflores@yahoo.com.br

Palavras-chave: HIV, HBV, HCV, SSPF

Introdução: Os vírus HIV, HBV e HCV possuem as mesmas vias de transmissão, influenciando no aumento da prevalência e coinfecções, principalmente em indivíduos e usuários de droga, promiscuidade sexual e vulnerabilidade social. **Métodos:** O uso de amostras biológicas alternativas para o diagnóstico é muito importante, tais como a saliva e o sangue seco em papel de filtro (SSPF) facilitariam a detecção e o acompanhamento destes pacientes, em casos de pouco acesso à rede de saúde, vulnerabilidade social e em locais de difícil acesso. A determinação do grau de fibrose hepática e das concentrações de citocinas também se faz importante no tratamento e monitoramento destes pacientes. **Conclusões:** Poucos dados estão disponíveis sobre os métodos não

invasivos para avaliação da fibrose hepática e sobre o papel de citocinas e nestes pacientes. Então, é muito importante avaliar novas metodologias laboratoriais em fluidos corporais alternativos para diagnóstico e avaliação imunológica principalmente nos casos de coinfecção.

Apoio Financeiro: CAPES e IOC.

Participante: **Iukary Oliveira Takenami**

Título: **28. Avaliação de mediadores inflamatórios e lipídicos como potenciais biomarcadores na distinção da tuberculose ativa e latente**

Autores: Takenami, I.[1]; Santos, EB[2], Oliveira, CC.[1], Arruda, S.[1]

Filiação: [1]Laboratório Avançado de Saúde Pública (LASP), Centro de Pesquisa Gonçalo Moniz – CPqGM/FIOCRUZ, Salvador. [2]6° Centro de Saúde Rodrigo Argolo, Salvador, Bahia, Brasil.

iukary@yahoo.com.br

Palavras-chave: tuberculose ativa, tuberculose latente, biomarcador e citocinas.

Introdução: A maioria dos casos de Tuberculose (TB) origina-se nos indivíduos com a forma latente da doença (TBL). As lacunas no conhecimento sobre os parâmetros biológicos associados à progressão da latência para a doença ativa tem impedido maiores avanços na proposição de estratégias vacinais e terapêuticas que auxiliem de forma mais eficaz o controle da doença. Nesse contexto o objetivo do estudo é identificar um potencial biomarcador com base na produção de citocinas e de eicosanóides que possa predizer i risco de reativação da TBL para a TB ativa. **Métodos e resultados:** Uma coorte prospectiva de pacientes com TB e de seus respectivos comunicantes domiciliares está sendo acompanhada no 6° Centro de Saúde Rodrigo Argolo. Entre Janeiro de 2012 e Março de 2013 foram identificados 46 pacientes com TB pulmonar e 74 comunicantes domiciliares. Dos 46 pacientes identificados, 69% (32/46) eram homens, 34,8% tinham entre 26-35 anos, 45,6 e 41,3% se

autorreferiram de raça parda e negra, respectivamente; somente um dos 46 casos era HIV positivo. Dos 74 comunicantes domiciliares, 68,5% eram do sexo feminino e 51,8 tinham entre 0-25 anos. A prevalência de infecção foi de 56,8%. **Conclusão**: O perfil de pacientes e comunicantes corresponde à tendência no Brasil, reforçando a associação da doença com o sexo masculino em idade produtiva da vida. Enquanto que o perfil da população de comunicantes é de mulheres entre 0-25 anos. Os dados apresentados são preliminares, as dosagens dos mediadores ainda não foram realizadas. Dessa forma, novos dados são esperados para análise posterior.

Auxílio: Capes, CPqGM.

Participante: **Izabelle Vianna de Vasconcelos**

Título: **29. Fábricas sociais como metodologia inclusiva para o enfrentamento de parasitoses intestinais em área hiperendêmica de habitações precárias no Distrito do Murinim, Benevides, Pará**

Autores: Izabelle Vianna De Vasconcelos[1], Ligia de Salazar[2], Antonio Henrique Almeida de Moraes Neto[3]

Filiação: [1]Universidade da Amazônia-UNAMA, Belém, Pará (PA)
[2]Fundación para el Desarrollo de la Salud Pública, Cali, Colômbia
[3]Laboratório de Inovações em Terapias, Ensino e Bioprodutos – LITEB, Instituto Oswaldo Cruz – IOC, FIOCRUZ, RJ

izabellevianna@gmail.com; ahmn@ioc.fiocruz.br

Palavras-chave: Parasitoses Intestinais, Fábricas Sociais, Habitações Precárias, Plano *Brasil Sem Miséria*

Em Benevides, PA, a situação de morbi-mortalidade está relacionada à falta de acesso ao saneamento básico. Os dados da matriz de agravos à saúde elaborada de forma participativa entre os Agentes Comunitários de Saúde identificaram como principal agravo as parasitoses intestinais e como soluções as melhorias habitacionais e geração de renda. Nosso

objetivo é desenvolver modelo para enfrentamento das parasitoses intestinais e doenças diarreicas, usando como ferramenta a produção de fábricas sociais de artefatos de pré-moldados para a construção civil, como metodologia inclusiva, em área hiperendêmica de habitações precárias. O impacto das melhorias será avaliado tendo como parâmetros a pesquisa qualitativa e o status parasitológico dos moradores, antes e após as melhorias. A primeira fase da construção da Fábrica Social de pré-moldados ecológicos foi concluída em parceria com a Prefeitura e comunidade. As habitações foram selecionadas usando-se como critério os resultados do status parasitológico dos moradores. Este projeto acaba de estabelecer-se no Município como Programa de Governo, existindo já o espaço físico para o funcionamento da fábrica, o desenvolvimento do produto e formação de cooperativa. Pretende-se através desta metodologia apoiar o Plano *Brasil Sem Miséria* e o Sistema Único de Saúde, visando o enfrentamento da transmissão das parasitoses intestinais, assim como a qualificação de mão de obra e fomento a economia popular e solidária.

Auxílio: LITEB-IOC/FIOCRUZ, UNAMA, Prefeitura Municipal de Benevides – PA

Participante: **José Rivaldo Melo de França**

Título: **30. Plano Brasil sem Miséria: a frágil receita disponível dos municípios de Minas Gerais para a proposta de custeio da atenção básica**

Autor: José Rivaldo Melo de França[1]

Orientador: Nilson do Rosário Costa[2]

Filiação: 1 – Ministério da Saúde, Brasília; 2 – ENSP/Fiocruz, Rio de Janeiro.

jrmf1@uol.com.br

Palavras-chave: financiamento; gestão administrativa; tendências.

Resumo: Este trabalho analisa a sustentabilidade econômico-financeira requerida para a cobertura das metas de AB nos municípios de MG selecionados pelo Governo Federal para atuação em saúde no âmbito do Plano Brasil sem Miséria (BSM). Para desenvolver o estudo foram pesquisadas as bases de dados do Siops/MS, Sarge/MS e IBGE. Para caracterizar o problema foram adotados portes populacionais. Os indicadores utilizados foram criados por Pereira et al. (2006). Utilizaram-se também indicadores testados Mendes (2010), além de outros pertinentes introduzidos pelos autores. A pesquisa foi estruturada em três dimensões: Recursos originários das Receitas Disponíveis (RD) e das Transferências do SUS; Despesas municipais com saúde e Alocação municipal e transferências federais para AB e cobertura das linhas de atuação estruturantes. Os resultados obtidos permite concluir que para os municípios com até 50 mil habitantes, 95,2% do total com atuação em saúde no BSM, ampliarem suas metas ou linhas de atuação nas propostas do BSM necessitarão de aprimorar sua capacidade tributária ou recorrerem a recursos suplementares das demais esferas, dada a exígua margem de RD em que operam e os elevados níveis de cobertura em AB básica que já atingiram utilizando, principalmente, suas transferências constitucionais e legais.

Participante: **Julia Modesto Pinheiro Dias Pereira**

Título**: 31. Pactuação Federativa e o Programa Bolsa Família**

Autores: Julia Modesto Pinheiro Dias Pereira

Filiação: Programa de Pós-Graduação em Políticas Públicas, Estratégia e Desenvolvimento (em associação com a FIOCRUZ/MS) - UFRJ - Rio de Janeiro

julia_modestopdpereira@yahoo.com.br

Palavras-chave: Programa Bolsa Família; federalismo, gestão estadual

Introdução: Após a Constituição de 1988 o Brasil ao confirmar a escolha de ser um país federativo, também optou pela descentralização de

suas políticas – entre elas as políticas de assistência social. Como um exemplo de política descentralizada temos o programa de transferência condicionada de renda (Programa Bolsa Família-PBF). De forma que o objetivo deste trabalho será estudar as relações federativas presentes na gestão do PBF, com foco na participação dos estados, visando compreender se os estados inseriram-se no processo ou se eles formam inseridos e quais os fatores que fazem com que os estados sejam mais ou menos cooperativos nesse processo? **Metodologia:** Construção de um indicador de panorama das gestões por intermédio de indicadores que tragam um panorama da Assistência Social nos estados, tais como a Pesquisa de Informações Estaduais. Após a construção desse indicador, escolher dois estados para a realização de entrevistas em profundida com pessoas chaves responsáveis pela gestão do Programa Bolsa Família no âmbito estadual e do governo federal. **Conclusões:** Essa pesquisa encontra-se em fase de planejamento, no entanto, o seu potencial será o de perceber estratégias que facilitem a coordenação das diferentes áreas envolvidas na gestão do Programa Bolsa Família, e com isso possam promover de fato as condicionalidades como um meio de acesso aos serviços e consequentemente a cidadania.

Auxílio: CAPES

Participante: **Juliana Carolina Amorim**

Título**: 32. Fosfoproteoma quantitativo de *Trypanosoma cruzi* em diferentes fases de aderência na metaciclogenese**

Autores: Juliana Carolina Amorim[1], Aline Castro Rodrigues Lucena[1], Carla Vanessa de Paula Lima[1], Michel Batista[1], Najua Zahra[1], Fabricio Klerynton Marchini[1],

Filiação: [1] Laboratório de Gênomica Funcional. Instituto Carlos Chagas - FIOCRUZ, Panará

marchinifk@fiocruz.br

Palavras-chave: metaciclogênese, fosfoproteínas, fosfoproteômica.

Introdução: A metaciclogênese é o processo no qual a forma epimastigota de *Trypanosoma cruzi* diferencia-se em tripomastigota metacíclico e com isso adquire potencial de infecção. A progressão do ciclo de vida para o estado infectivo ocorre a partir de regulação da expressão a nível pós-transcricional, como em outras fases de diferenciação do parasita. Entre os mecanismos de regulação pós transcricional encontra-se a fosforilação de proteínas, que é uma modificação pós-traducional mediada pela ação de proteino-quinases e revertida por fosfatases. O presente trabalho pretende identificar, quantificar sítios de fosforilação durante a fase de aderência do parasita e caracterizar os mecanismos moleculares que estes regulam. Estes resultados contribuirão para o esclarecimento de quais proteínas ou vias metabólicas controladas por fosforilação ou desfosforilação, estão relacionados com o disparo e manutenção dos processos celulares relacionados à aquisição da infectividade em *T. cruzi*.
Metodologia: Dentro de um panorama atual de técnicas de proteômica, o desenvolvimento desse projeto tem como objetivo caracterizar o fosfoproteoma quantitativo de *T. cruzi* em fases de aderência da metaciclogênese. Fazendo uso da metodologia SILAC de quantificação de peptídeos, ensaios de enriquecimento dos fosfopeptídeos e análise por espectrômetro de massas com a plataforma LTQ Orbitrap XL ETD.

Auxílio: CAPES, ICC, CNPq

Participante: **Juliana Macedo Lacerda Nascimento**

Título: **33. Filmes de ficção científica: potencialidades e possibilidades de uso como recurso no ensino do genoma em áreas carentes do Estado do Rio de Janeiro**

Autores: Juliana Macedo Lacerda Nascimento e Rosane Moreira Silva de Meirelles

Filiação: Laboratório de Inovações em Terapias, Ensino e Bioprodutos. Instituto Oswaldo Cruz, FIOCRUZ, Rio de Janeiro.

juliana.macedo@ioc.fiocruz.br; rosanemeirelles@yahoo.com.br

Palavras-chave: Genoma; ficção científica; Aprendizagem Significativa.

Pesquisas têm mostrado que estudantes apresentam concepções sobre o genoma e sua funcionalidade influenciadas, sobretudo, em ideias midiáticas e que, por esse fato, há um grande fosso entre os saberes escolar, popular e científico em torno desse tema. Pelas interfaces que o conceito de genoma apresenta com tópicos pertinentes ao ensino de Biologia, este projeto objetiva-se a investigar como filmes de ficção científica podem ser utilizados no ensino público, visando mitigar tais distâncias que configuram a miséria educacional sobre esse tema. Para essa investigação, serão coletados dados de alunos do 1º e 3º ano do Ensino Médio e de professores de Biologia de seis escolas públicas localizadas em áreas carentes do Estado do Rio de Janeiro através de questionários e entrevistas. Através dos instrumentos de coleta, buscaremos investigar as concepções prévias dos alunos sobre genoma; com que frequência e de que formas os professores têm abordado tal conceito e qual o acesso a mídias nas escolas. Através deste projeto, pretendemos estabelecer uma sequência de oficinas didáticas utilizando filmes de ficção científica que retratem o tema genoma promovendo no ambiente escolar, discussões sobre os avanços científicos e hábitos para a promoção da saúde que estão diretamente relacionados ao tema. As concepções pré e pós-oficinas didáticas serão analisadas à luz da Teoria da Aprendizagem Significativa.

Auxílio: CAPES, IOC

Participante: **Karina Cabello**

Título: **34. Aplicação e avaliação do impacto de ações educativas sobre os indicadores da infecção por esquistossomose em escolares de Malacacheta, município com alto percentual de pobreza situado na área endêmica de Minas Gerais**

Autores: Karina Cabello[1], Cristiano Massara[2], Lilian Beck[1], Virginia Schall[3], Otavio Pieri[1], Tereza Favre[1*]

[1] Laboratório de Ecoepidemiologia e Controle da Esquistossomose e Geohelmintoses (LECEG). Instituto Oswaldo Cruz – FIOCRUZ, Rio de Janeiro

[2] Laboratório de Helmintologia e Malacologia Médica. [3] Laboratório de Educação em Saúde. Centro de Pesquisa René Rachou – FIOCRUZ, Minas Gerais
[*] Orientador

karina.cabello@ioc.fiocruz.br

Palavras-chave: esquistossomose, epidemiologia, educação em saúde.

A esquistossomose é endêmica em vários estados brasileiros. As áreas mais afetadas caracterizam-se por condições precárias ou inexistentes de saneamento, pobreza e baixos níveis de escolaridade. Há um consenso entre os profissionais que atuam na interface saúde-educação de que a realização de ações educativas, conjugadas ao tratamento e saneamento, constituem as medidas mais eficazes para o controle da endemia. Este estudo pretende avaliar se o emprego de ações educativas voltadas para prevenção e controle, conjugadas ao tratamento e dirigidas aos escolares do município de Malacacheta, MG, contribui para ampliar o conhecimento, estimular a adoção de práticas preventivas e reduzir prevalência e/ou intensidade da esquistossomose. O conhecimento dos escolares será avaliado por um questionário aplicado antes, um mês, seis e doze meses depois do desenvolvimento das atividades educativas. O status de infecção será acompanhado através de exames de fezes (Kato-Katz) realizados antes e 45 dias, seis, 12 e 24 meses após o tratamento dos infectados. As seguintes atividades foram realizadas: (a) reuniões com os responsáveis e assinatura do TCLE; (b) aplicação dos questionários para avaliar o conhecimento prévio dos escolares; (c) exames parasitológicos e (d) tratamento dos infectados. Dos 3.088 matriculados, 2.525 (81,8%) escolares realizaram o exame. A prevalência foi 21,4%. Dos 540 infectados, 515 (95,4%) foram tratados com praziquantel. Na próxima etapa, será realizado o I Curso Básico de Esquistossomose para os professores das escolas experimentais (onde as ações educativas serão realizadas), através do qual serão capacitados para desenvolver atividades educativas com seus alunos no ambiente de sala de aula.

Auxilio: IOC, FIOCRUZ/CAPES BSM, PAPES/FIOCRUZ

Participante: **Lia Gomes**

Título: **35. Sequenciamento de nova geração em *Mycobacterium tuberculosis* Beijing e análise comparativa in sílico de mutações em H37Rv e H37Ra.**

Autores: Lia Gomes[1]; Elena Lassounskaia[3]; Marcelo Araujo[1]; **Sidra Vasconcellos[1]**, Atiná Ribeiro [1]; Harrison Gomes[1]; Antonio de Miranda [2]; Philip Suffys[1].

Filiação: [1] Laboratório de Biologia Molecular Aplicada a Micobactérias. Instituto IOC- FIOCRUZ, Rio de Janeiro, [2] Laboratório de Biologia Computacional . Instituto IOC- FIOCRUZ, Rio de Janeiro, [3] Laboratório de Biologia do Reconhecer. UENF, Rio de Janeiro.

lialimagomes@hotmail.com

Palavras-chave: Mycobacterium tuberculosis Beijing, sequenciamento de nova geração.

O sucesso endêmico da família *M. tuberculosis* Beijing (MtbB) em diferentes regiões geográficas têm sido atribuído a alta transmissão, hipervirulência e resistência aos fármacos, que geralmente estão relacionados com mutações em genes específicos. Estudos em macrófagos THP1 demonstraram maior virulência entre cepas MtbB circulante na Rússia em comparação com cepas circulantes no Brasil. Desta forma, o objetivo do presente estudo foi comparar a frequência de mutações entre genes de MtbB e cepas H37Rv e H37Ra. Para isto, analisou-se por sequenciamento genômico duas cepas MtbB (Rússia e Brasil). A análise comparativa da cepa MtbB da Rússia demonstrou maiores frequências de mutações em genes relacionados com metabolismo de lipídio, parede celular e processos celulares, metabolismo intermediário, em comparação com a H37Rv e H37Ra que a cepa ZT272. Contudo, as duas cepas analisadas apresentaram maiores frequências de mutação para a H37Rv em comparação com a cepa atenuada H37Ra, este resultado pode estar associado com o fato da H37Rv possuir sua própria complexidade genética.

Auxílio: CAPES, IOC.

Participante: **Ligia Cristina Kalb Souza**

Título: **36. Componentes celulares essenciais como alvos para o tratamento da Doença de Chagas: o papel da clatrina na endocitose e nutrição do** *Trypanosoma cruzi* **(CHAGAS, 1909).**

Autores: Ligia Cristina Kalb Souza, Maurilio José Soares

Filiação: Laboratório de Biologia Celular, Instituto Carlos Chagas/ Fiocruz, Curitiba, PR

ligia_kalb@hotmail.com

Palavras-chave: clatrina, endocitose, *Trypanosoma cruzi*.

Introdução: A via endocítica de *T. cruzi* é um modelo interessante, pois difere daquelas presentes em mamíferos e *T. brucei*. Há um peculiar sistema de endocitose envolvendo pelo menos duas vias de entrada: a bolsa flagelar e o citóstoma. O presente projeto tem como objetivo verificar o papel da clatrina na endocitose de *T. cruzi*, tanto na bolsa flagelar quanto no citóstoma, por meio da depleção de clatrina e identificação de suas proteínas associadas. Métodos e Resultados: Anticorpos monoclonais para clatrina foram produzidos. Para verificar alterações celulares decorrentes da depleção da clatrina estamos desenvolvendo um parasita deficiente em clatrina por meio de nocaute gênico. Até o momento obtivemos uma população de parasitas resistentes à neomicina e à higromicina, onde não foi possível detectar a proteína clatrina. Estes parasitas foram submetidos a sorting asséptico para obtenção de uma população clonal e serão submetidos a ensaio de southern blot para confirmação do nocaute. Para identificar proteínas associadas à cadeia pesada de clatrina será feito ensaio de *cryogrinding* seguido de imunoprecipitação em colaboração com o Laboratório do Dr. Mark Field na Universidade de Cambridge.

Auxílio: CAPES, FIOCRUZ

Participante: **Luana Leandro Gois**

Título: **37. Estudo das alterações imunológicas na síndrome de reconstituição imune associada à coinfecção hiv e tuberculose**

Autores: Luana Leandro Gois[1], Robero Badaró[2], Maria Fernanda Rios Grassi[1]

Filiação: 1) Laboratório Avançado de Saúde Pública. Centro de pesquisa Gonçalo Moniz – FIOCRUZ, Salvador, Bahia, Brasil. 2) Universidade Federal da Bahia, Salvador, Bahia, Brasil.

lualeandro1@hotmail.com

Palavras-chave: Síndrome inflamatória de Reconstituição Imune, AIDS, Tuberculose, Resposta Imunológica

Resumo: A terapia antirretroviral (ART) tem sido fundamental no controle da AIDS. Contudo, alguns pacientes, apesar da redução da carga viral e do aumento de linfócitos T CD4+, apresentam Síndrome Inflamatória de Reconstituição Imune (IRIS). A tuberculose (TB) é a doença mais associada a IRIS (TB-IRIS). Sugere-se que a IRIS ocorre devido ao desequilíbrio entre a restauração da resposta inflamatória e regulatória. Entretanto, a imunopatogenese da IRIS não está definida. Este estudo avaliará a restauração da resposta imune em pacientes com TB-IRIS. Será realizado um estudo piloto, longitudinal, avaliando 80 pacientes divididos nos subgrupos: 1) indivíduos infectados pelo HIV com TB-IRIS; 2) indivíduos infectados com HIV e com infecção latente por Mtb virgens de ART; 3) indivíduos infectados com HIV e com infecção latente por Mtb sob ART a menos de seis meses; 4) indivíduos não infectados por HIV com TB. Os indivíduos serão avaliados em cinco momentos. A avaliação clínica constará de: contagem de linfócitos T $CD4^{+}$, carga viral do HIV, raio-X de tórax, cultura de escarro e teste intradérmico a tuberculina; e a imunológica: quantificação de citocinas e quimiocinas, avaliação da proliferação dos linfócitos T em resposta aos antígenos do Mtb, quantificação de linfócitos T regulatórios e avaliação do estágio de ativação dos linfócitos T. Estes resultados contribuirão para compreender

os mecanismos patofisiológicos da IRIS e identificar marcadores preditores do desenvolvimento de IRIS.

Auxílio: CAPES, FAPESB

Participante: **Luciane Amorim Santos**

Título: **38. Identificação de SNPS no genoma da *leptospira interrogans* sorovar Copenhageni e possível associação ao desfecho clínico e Síndrome Hemorrágica Pulmonar**

Autores: Luciane Amorim Santos[1,2], Xiting Yan[2], Elsio Wunder Jr[2], Haritha Adhikarla[2], Albert Ko[2].

Filiação: [1]Laboratório de Hematologia, Genética e Biologia Computacional, Centro de Pesquisa Gonçalo Moniz - FIOCRUZ, Salvador
[2]Yale School of Epidemiology and Public Health, Yale University, New Haven, USA

luciane.santos@yale.edu

Palavras-chave: Leptospirose.

Introdução: Leptospirose é uma zoonose causada por bactérias do gênero *Leptospira.* A rápida urbanização e o crescimento do número de favelas estimulou o aumento de casos da doença. Endêmica na cidade de Salvador, BA, a doença está associada com a estação chuvosa, com mais de 12.000 casos anuais. As manifestações clínicas variam de assintomática à formas graves com letalidade de 10%. Em 2003, começaram a ser diagnosticados casos de SHPS, fatal em 55% dos casos. Este estudo tem como objetivo identificar diferenças genéticas entre isolados de *Leptospira interrogans* serovar Copenhageni, e suas associações com diferentes desfechos clínicos. Métodos e Resultados: Neste estudo foram sequenciados o genoma de 96 isolados clínicos. Foram detectados 419 SNPs, sendo 275 localizados em regiões intragênicas e distribuídos em 235 genes. Os resultados indicam que os isolados são conservados. Será realizado a associação destas mutações com diferentes desfechos clínicos. Conclusões: A leptospirose é um problema que atinge áreas

urbanas e populações de baixa renda. Elucidar os mecanismos genéticos associados com os diferentes desfechos clínicos da doença irá contribuir na identificação de possíveis candidatos para diagnóstico e vacina.

Auxílio: CAPES, CPqGM, CNPq e NIH

Participante: **Ludimila Santos Amaral**

Título**: 39. Monitoramento por métodos de biologia molecular e imunológicos de enteroparasitas em areia de praias**

Autores: Ludimila Santos Amaral[1], Antonio Nascimento Duarte[2], Elvira Carvajal[3], Luiz Carlos Leite Pinna[2]; Valmir Laurentino Silva[2], Adriana Sotero-Martins[4]

Filiação: [1]Pós-Graduação em Saúde Pública e Meio Ambiente-ENSP/FIOCRUZ, RJ; [2]Departamento de Ciências Biológicas -ENSP/FIOCRUZ, RJ; [3]Departamento de Biologia e Celular - IBRAG-UERJ, RJ; [4]Departamento de Saneamento e Saúde Ambiental- ENSP/FIOCRUZ, RJ

ludimilasa@ensp.fiocruz.br

Palavras-chave: parasito; PCR; ELISA

Introdução: As avaliações das condições de vida, como a situação de moradia e do saneamento básico oferecido à população são, em grande parte, determinantes da transmissão de parasitos. Alguns parasitos, como *Entamoeba histolytica, Giardia intestinalis, Ascaris lumbricoides, Trichuris trichiura* são transmitidos pela água ou alimentos contaminados, enquanto outros, como *Ancylostoma duodenale e Strongyloides stercoralis*, são transmitidos por larvas presentes no solo. O objetivo é monitorar as condições sanitárias das areias da Baía de Guanabara – RJ e da Bacia do Amazonas por meio de imunodiagnósticos e moleculares de parasitoses intestinais com especificidade elevada, visando comparar com métodos tradicionais. **Métodos e resultados**: Amostras de areia serão selecionadas e após a realização de exame parasitológico, serão analisadas por técnicas de PCR e de ELISA. Estão sendo desenvolvidos oligonucleotídeos baseados

nas sequencias depositadas no GeneBank para *Ancylostoma sp., Ascaris sp, Trichuris sp., Toxocara sp. Entamoeba sp., Giardia sp., Cryptosporidium sp.* para identificação específica por PCR **Conclusões:** Contribuir para o entendimento epidemiológico das parasitoses nos ambientes estudados, ressaltando questões de saúde e risco ambiental em áreas de impactadas por esgoto sanitário.

Auxílio: CAPES

Participante: **Marcus Vinicius Campos Matraca**

Título: **40. A Dialogia do Riso e as Expedições Científicas da Fiocruz no Plano Brasil Sem Miséria.**

Autores: Marcus Vinicius Campos Matraca[1], Tania Cremonini de Araújo - Jorge[2].

Filiação: [1, 2] Laboratório de Inovações em Terapias, Ensino e Bioprodutos

matraca@ioc.fiocruz.br; taniaaj@ioc.fiocruz.br

Introdução: Esta pesquisa investiga a ressignificação e retomada das Expedições Científicas da Fundação Oswaldo Cruz para promoção da saúde e enfrentamento das doenças negligenciadas no Plano Brasil sem Miséria. **Referencial teórico:** embasamos o trabalho em Paulo Freire, Mikhail Bakhtin, Carlos Brandão, Harold Becker, Nize Da Silveira, Victor Valla dentre outros. **Objetivo:** produzir compartilhadamente com os expedicionários e os moradores locais uma leitura sócio cultural acerca da experiência propiciada pelas expedições do IOC pelo Brasil sem Miséria criando estratégias dialógicas para compartilhar saberes entre todos que estão/estarão atuando nos territórios visitados. **Metodologia:** Adotamos a pesquisa participante e a pedagogia libertária como referenciais metodológicos. Trata-se de uma investigação que busca na educação dialógica, garantir a participação coletiva na construção dos saberes. **Resultados:** Produção de artigos, oficina de trabalho e Podcast. **Discussão:** A Dialogia do Riso baseada na ciência e arte exerce papel

fundamental não só no processo de aprendizagem em saúde, como também na relação do cidadão com o seu meio ambiente. Esta proposta de investigação se justifica pois vem para agregar as expedições cientificas articuladas ao Plano Brasil Sem Miséria. **Conclusão:** Nas expedições que realizamos (Paudalho – PE e Rio Branco – AC), concluímos a necessidade de misturar os saberes popular com o científico. As soluções não são simples, exigindo novas experiências para lidar com adversidades sociais emergentes, que não pertencem apenas aos países em desenvolvimento.

Auxílio: CAPES

Participante: **Maria Clara Coelho Camara**

Título**: 41. Dinâmica da escolha alimentar familiar de mulheres beneficiárias do Programa Bolsa Família no Distrito Federal e no Rio de Janeiro**

Autores: Maria Clara Coelho Camara[1], Denise Oliveira e Silva[2], Denise Cavalcante[3], Maria Cristina R. Guilan[3]

Filiação: [1]Pos doutoranda do PBM. FIOCRUZ, Rio de Janeiro
[2] Pesquisadora da Diretoria Regional de Brasília FIOCRUZ, Brasília
[3] Pesquisadora da Escola Nacional de Saúde Pública. FIOCRUZ, Rio de Janeiro.

mclaracc@gmail.com; deniluz@fiocruz.br

Palavras-chave: segurança alimentar; bolsa família; etnografia

O Plano Brasil sem Miséria surge no contexto de acabar com a fome no Brasil. Entre as ações que envolvem o PBSM está a transferência de renda, acesso a serviços públicos e a inclusão produtiva. O Bolsa Família é um dos principais programas de transferência de renda no país e uma das estratégias do PBSM. É fundamental conhecer e auxiliar as famílias, principalmente as mulheres, sobre "o que comprar" com os recursos, com escolhas alimentares mais adequadas e saudáveis. Este estudo tem como objetivo analisar a dinâmica do processo de escolha

alimentar de beneficiárias de Programa Bolsa Família e sua influência na percepção do conceito de segurança alimentar e nutricional, no bairro de Manguinhos e no conjunto habitacional de Vila Kennedy na cidade do RJ e de Sobradinho no Distrito Federal. Trata-se de estudo qualitativo pela utilização de abordagem etnográfica e análise hermenêutica. Será realizado pela observação direta da dinâmica de escolha de alimentos relacionados aos equipamentos públicos e privados para a aquisição de alimentos e sua preparação culinária no domicílio.

Participante: **Marilda Maria da Silva Moreira**

Título: **42. Tecnologia sociais para controle das helmintoses: proposições ao Plano Brasil Sem Miséria para enfrentamento das doenças infecciosas relacionadas à pobreza**

Autores: Marilda Maria da Silva Moreira[1], Márcia de Oliveira Teixeira[2], Antonio Henrique Almeida de Moraes Neto[3]

Filiação: [1]Laboratório de Educação Profissional em Vigilância em Saúde - LAVSA/Escola Politécnica da Saúde Joaquim Venâncio - EPSJV/FIOCRUZ, Rio de Janeiro;
[2]Centro de Estudos - EPSJV/FIOCRUZ, Rio de Janeiro;
[3]Laboratório de Inovações, Terapias, Ensino e Bioprodutos – LITEB/Instituto Oswaldo Cruz - IOC, Fiocruz, Rio de Janeiro

marildamoreira@fiocruz.br

Palavras-chave: Políticas Públicas, Tecnologias Sociais (TS), Biodiversidade, Doenças Negligenciadas, Determinantes Sociais da Saúde.

Desde o nascimento da República, diferentes pensadores empenharam esforços para explicitar as iniquidades sociais existentes em nosso país. Mais de um século depois, ainda convivemos com quadros agudos de sofrimento da população brasileira, aduzidos à emergência de novas doenças e à negligência daquelas indiscutivelmente relacionadas à pobreza, como as helmintoses, cuja estimativa de casos vem sendo subestimada devido à ausência de registro compulsório destes agravos.

Nosso objetivo é analisar o processo de implementação de Tecnologias Sociais (TS) para o enfrentamento das helmintoses em áreas de vulnerabilidade socioambiental, no entorno da Fiocruz, no âmbito do Plano *Brasil Sem Miséria*. A pesquisa é de natureza qualitativa, centrada na pesquisa-ação, buscando o encaminhamento de ações concretas e esclarecimento dos problemas decorrentes da situação observada, no referencial da Promoção da Saúde, visando à mitigação da transmissão. Este estudo tem priorizado a construção de quadros analítico-conceituais sobre: políticas públicas, tecnologias sociais e a dinâmica saúde/ambiente. Espera-se que esta pesquisa possa identificar TS propícias aos cuidados e prevenção das infecções por helmintos e outras doenças negligenciadas, assim como possa contribuir para ampliação do debate sobre a complexidade do ciclo doença-pobreza, os direitos sociais e as políticas públicas.

Auxílio: IOC e EPSJV/FIOCRUZ

Participante: **Mauricio Lisboa Nobre**

Título: **43. Identificação das fontes de infecção como estratégia para bloquear a transmissão da Hanseníase em município hiperendêmico – Mossoró/RN**

Autores: Mauricio Lisboa Nobre [1,2], Mariana de Andrea Vilas Boas Hacker [1], Selma Maria Bezerra Jerônimo [2], Euzenir Nunes Sarno [1]

Filiação: [1] Laboratório de Hanseníase. Instituto Oswaldo Cruz. Fundação Oswaldo Cruz. Rio de Janeiro. [2] Departamento de Bioquímica e Instituto de Medicina Tropical da Universidade Federal do Rio Grande do Norte.

nobreml@gmail.com

Palavras-chave: hanseníase, epidemiologia, transmissão, prevenção e controle.

Introdução: A hanseníase é um importante problema de saúde pública no Brasil, especialmente em municípios como Mossoró/RN, onde o coeficiente de detecção atingiu 41,2 casos por 100.000 habitantes

em 2012. **Métodos e Resultados:** Após georreferenciamento de 808 casos de hanseníase residentes em Mossoró/RN, identificou-se área hiperendêmica onde será realizada busca ativa de casos em 3.500 estudantes de 10 escolas públicas. Previamente será ministrado curso de extensão universitária em Hansenologia para 50 estudantes de medicina da UERN; o exame dos escolares será realizado por estes estudantes sob supervisão de hansenólogo. Até o momento houve três reuniões nas Secretarias Municipais de Saúde e Educação para discussão do projeto, além de um encontro com 180 professores, onde definiram-se as estratégias para educação em saúde que estão sendo desenvolvidas nas escolas no mês de julho como preparação para a busca ativa que ocorrerá em agosto de 2013. Após a busca ativa serão realizadas visitas domiciliares para exame de comunicantes dos casos diagnosticados, com o objetivo de identificar as fontes de infecção, buscando-se tratá-las interrompendo a cadeia de transmissão da doença no município. O projeto foi aprovado pelo CEP-UFRN (CAAE: 06189612.9.0000.5537). **Conclusões:** Constatou-se grande interesse das autoridade locais e demais parceiros envolvidos, com importante adesão ao projeto e aos seu objetivo de reduzir a endemia em Mossoró/RN.

Participante: **Maykol Adrian Noll**

Título: **44. Desenvolvimento de um sistema para produção em larga escala de anticorpos contra proteínas de *Trypanosoma cruzi***

Autores: Maykol Adrian Noll, Marco Aurélio Krieger, Daniela Parada Pavoni, Christian Macagnan Probst

Filiação: Instituto Carlos Chagas, FIOCRUZ

Muitas pesquisas sobre o *Trypanosoma cruzi*, causador da Doença de Chagas, empregam anticorpos para caracterizar proteínas e desenvolver estratégias de diagnóstico, mas podem ser prejudicados pela dificuldade de se obter tais reagentes em quantidade necessária e com reatividade apropriada. Neste sentido, o presente trabalho se propõe a estabelecer uma metodologia de produção em larga escala de anticorpos com elevada afinidade e especificidade por proteínas de *T. cruzi*. Inicialmente,

porções imunogênicas de sequências polipeptídicas serão identificadas, expressas e inoculadas em camundongos, a partir dos quais linfócitos produtores de imunoglobulinas serão isolados através de citometria de fluxo. Os genes que codificam as regiões variáveis de anticorpos serão amplificados destas células individualizadas, fusionados e expressos como fragmentos de cadeia única, que depois de caracterizados poderão ser destinados a diversas aplicações. Aqui são descritas as etapas iniciais do desenvolvimento deste projeto, em particular a construção da ferramenta de análise bioinformática para predição da antigenicidade de proteínas com base em propriedades físico-químicas e estruturais e em alinhamentos comparativos. Esta última abordagem evitará a geração de anticorpos com reatividade cruzada e contribuirá para o estudo dos supergenes de *T. cruzi* – grupos de sequências muito semelhantes resultantes de redundância genômica ou problemas durante o sequenciamento –, possibilitando a obtenção de imunoglobulinas dirigidas a uma família inteira de supergenes, ou específicas para alguns de seus elementos.

Participante: **Nadia Bomfim do Nascimento**

Título**: 45. Mapeamento, Monitoramento e Avaliação Participativa dos Projetos de Pesquisa na Fiocruz relacionados ao Plano Brasil Sem Miséria.**

Autores: Nadia Bomfim do Nascimento[1], Maria Cristina Rodrigues Guilam[2],

Filiação: [1,2]Coordenação Geral de Pós-graduação.

nadia.b.nascimento@gmail.com

Palavras-chave: politicas públicas; programas sociais; miséria; avaliação participativa

O Plano Brasil sem Miséria, através de ações nacionais e regionais, objetiva elevar a renda e as condições de bem estar da população. Organiza-se em três eixos: transferência de renda, acesso a serviços e inclusão produtiva.

A Fiocruz celebrou acordo de cooperação técnica com MDS e de fomento e indução de teses de pós-graduação com a Capes para incentivar pesquisas voltadas para essa temática. O presente projeto objetiva articular e integrar tais pesquisas. O mapeamento e monitoramento dos projetos inseridos no BSM, através da utilização de grupos focais, fóruns e seminários, possibilitarão a inserção dos pesquisadores no processo de avaliação participativa, que implicará na definição de caminhos e responsabilidade para cada ação particular. A avaliação participativa possibilita uma apropriação reflexiva e socializada de todo o processo por parte dos atores institucionais envolvidos. A etapa de mapeamento dos projetos inseridos no BSM já foi concluída, e possibilitou a emergência de algumas categorias de analise, tais como: Unidades técnico-científicas da Fiocruz com projetos inseridos no BSM; estados e regiões do país objeto de estudo nas pesquisas inseridas no programa; inserção dos projetos por eixo temático; dentre outros. O primeiro artigo referente à descrição e análise preliminar do mapeamento dos projetos encontra-se em curso.

Auxílio: CAPES

Participante: **Naiara Carvalho Teixeira**

Título: **46. Contribuição para o desenvolvimento de uma vacina contra leishmaniose visceral canina**

Autores: Naiara Carvalho Teixeira[1], Jéssica Mariane Ferreira Mendes[1], Matheus Moreno Passos Barbosa [1], Cristiane Garboggini Melo de Pinheiro[1], Geraldo Gileno de Sá Oliveira[1].

Filiação: [1]Laboratório de Patologia e Biointervenção. Centro de Pesquisas Gonçalo Moniz – CPqGM. Instituto Oswaldo Cruz – FIOCRUZ/Bahia

naiara@aluno.bahia.fiocruz.br

Palavras-chave: leishmaniose visceral,vacina e proteína recombinante.

A leishmaniose visceral (LV) é uma doença parasitaria que se encontra em expansão no Brasil. O agente causal é o protozoário *Leishmania chagasi.* O

cão é considerado o reservatório do parasita. Visando contribuir para o desenvolvimento de uma vacina contra leishmaniose visceral canina, este trabalho tem como objetivos: selecionar antígenos através da avaliação da reatividade à células de cães resistentes a LV, estudar a distribuição do parasitismo no baço de cães com LV e determinar a atividade biológica e tóxica de rcaIL-12 em cães. Para isso, 2 proteínas de *L. chagasi,* sem domínios repetitivos de aminoácidos, foram subclonadas em pRSET e produzidas em *E. coli.* A obtenção de outras duas proteínas está em andamento. Posteriormente será avaliada a reatividade dessas proteínas a PBMC de cães resistente para LV. Para o estudo da distribuição do parasitismo no baço de cães com LV, punções em 9 pontos foram feitas em 6 animais para determinação da carga parasitária por PCR em tempo real. Visando utilizar no futuro como agente imunomodulador em uma vacina contra LV canina, a IL-12 foi produzida e doses diferentes serão administradas em animais sadios para avaliação de atividade funcional e toxicidade.

Financiamento: FIOCRUZ/CAPES, INCT-DT.

Participante: **Nathanielly Rocha Casado de Lima**

Título: **47. Vigilância Entomológica, morfometria clássica e ontogenética de triatomíneos em quatro municípios do Estado do Rio Grande do Sul associado a ações educativas na prevenção da Doença de Chagas**

Autores: Nathanielly Rocha[1], Jacenir Mallet[1]

Filiação: [1] Laboratório de Transmissores de Leishmanioses – Setor de Entomologia Médica e Forense. Instituto Oswaldo Cruz- FIOCRUZ, Rio de Janeiro.

natrocha@ioc.fiocruz.br

Palavras-chave: vigilância entomológica, morfometria, ações educativas.
Introdução: A doença de Chagas, denominada atualmente pela

Organização Mundial de Saúde e pela Organização Panamericana de Saúde como fazendo parte das "doenças infecciosas relacionadas à pobreza", através de proposta do Instituto Oswaldo Cruz, foi incluída dentre outras doenças, no Plano Brasil sem Miséria. Dentre as regiões abrangidas pelo plano a região sul apresenta importância nesse contexto com altos índices de infestação triatomínica e preocupação com espécies secundárias na transmissão da doença de Chagas, dado que tais espécies, em algumas regiões, já iniciaram um ciclo peridomiciliar e domiciliar. **Métodos:** Correlacionar a dinâmica destes vetores com o *Trypanosoma cruzi*, ampliando o conhecimento sobre as cepas que circulam na natureza, em um estudo multidisciplinar das populações de triatomíneos vetores e do parasito *Trypanosoma. cruzi*, em 5 municípios indicados pela Secretaria de Saúde do Estado do Rio Grande do Sul. Além disso, agregar ações educativas para sensibilizar e informar a população e profissionais de saúde, como forma de contribuir para a prevenção da doença de Chagas no Estado. **Resultados:** Foram coletados 200 espécimes de triatomíneos de 5 municípios do Rio Grande do Sul, dando início aos experimentos.

Auxílio: CAPES, IOC.

Participante: **Patrícia Lago Zauza**

Título: **48. Impacto da repetição do tratamento com benznidazol na resposta imune humoral, parasitemia e cardiopatia de pacientes portadores da Doença de Chagas crônica de Virgem da Lapa, Vale do Jequitinhonha, Minas Gerais.**

Autores: Patrícia Lago Zauza, Júlio César Miguel, José de Souza Nogueira, Sérgio Salles Xavier, José Borges-Pereira. Laboratório de Doenças Parasitárias do IOC/FIOCRUZ.

plz@ioc.fiocruz.br

O tratamento etiológico convencional da doença de Chagas crônica em adultos com o uso de benznidazol por 60 dias consecutivos na dose de 5mg/kg/dia tem apresentado resultados insatisfatórios, indicando que novas estratégias terapêuticas devem ser testadas. Nessa direção será

realizado um estudo de 4 anos, no qual pacientes chagásicos crônicos (PCC) naturais de Virgem da Lapa, MG serão submetidos ao tratamento com benznidazol por 60 dias/ano, um, dois e três anos consecutivos. Para isso, foram sorteados 150 PCC de ambos os gêneros, de 40 a 60 anos, sem ou com cardiopatia leve (conforme OPAS 1974 e Consenso Brasileiro em Doença de Chagas 2005). Desses 150 PCC, por sorteio simples, foram compostos os grupos PCC1T (n=50, tratamento em 2013), PCC2T (n=50, tratamentos em 2013 e 2014) e PCC3T (n=50, tratamentos em 2013, 2014 e 2015). No seguimento será feito o monitoramento anual da resposta imune humoral através de testes sorológicos de imunofluorescência indireta e ensaio imunoenzimático-ELISA, da parasitemia através de hemocultura e reação em cadeia da polimerase (PCR) e da cardiopatia através de exame clínico, eletrocardiograma de repouso e ecocardiograma bidimensional.

Auxílio: CAPES/IOC

CEP/FIOCRUZ: PARECER 304.607

Participante: **Raquel Martins Lana**

Título: **49. Malária e Dengue: Análise da dinâmica espacial e temporal em relação às alterações ambientais, climáticas e do comportamento humano no estado do Acre, região Amazônica.**

Autores: Lana RM[1], Honório NA[2], Monteiro AMV[3], Silva-Nunes M[4], Souza TH[5], Codeço CT[6]

Filiação: [1]Doutoranda em Epidemiologia em Saúde Pública, ENSP/FIOCRUZ/RJ
[2]Laboratório de Transmissores de Hematozoários, IOC/FIOCRUZ/RJ
[3]Departamento de Ciências da Saúde e Educação Física, UFAC
[4]Coordenadoria de Observação da Terra, INPE
[5]Secretaria de Saúde do Estado do Acre
[6]Programa de Computação Científica, PROCC/FIOCRUZ/RJ

Email: raquelmlana@gmail.com

Palavras-chave: malária, dengue, comportamento, ambiente

O Acre encontra-se numa transição epidemiológica caracterizada pela retração da malária para o Alto Juruá, e expansão do dengue nas áreas urbanas. Alvo de intenso investimento de programas de governo que focam principalmente do desenvolvimento econômico e social, o Acre sofre forte alteração de sua paisagem e no modo de viver de sua população. Dessa forma, torna-se indispensável uma caracterização e avaliação do impacto das mudanças ambientais e comportamentais na saúde humana, em particular, a malária e o dengue. Esse trabalho tem dois componentes. O primeiro é um estudo ecológico que visa caracterizar a dinâmica espaço-temporal dessas duas doenças em diferentes municípios do estado do Acre na última década e sua relação com a paisagem; o segundo, inicialmente restrito à malária, visa investigar o impacto dos principais programas de governo na vulnerabilidade e exposição da população a essa doença, por meio de questionário a ser desenvolvido e aplicado num estudo transversal em áreas com graus variados de exposição a esses programas.

Auxílio: CAPES, CNPq, PROCC

Participante: **Renata Pires Pesce**

Título: **50. Adesão à proposta da Estratégia Saúde da Família: olhar compreensivo sobre as famílias em elevada situação de vulnerabilidade.**

Autores: Renata Pires Pesce[1], Simone Gonçalves de Assis[1]

Filiação: [1]Centro Latino Americano de Estudos de Violência e Saúde Jorge Careli. Escola Nacional de Saúde Pública - FIOCRUZ, Rio de Janeiro.

renata.pesce@gmail.com

Palavras-chave: Estratégia Saúde da Família, vulnerabilidade social, adesão aos serviços de saúde.

A Estratégia Saúde da Família é considerada como estruturante dos

sistemas municipais de saúde, com potencial para provocar reordenação do modelo vigente de atenção através da assistência a famílias mais vulneráveis socialmente. O objetivo principal do projeto é acompanhar famílias atendidas pela Estratégia de Saúde da Família buscando compreender fatores que facilitam e/ou dificultam a adesão junto à essa equipe. Busca-se oferecer subsídios para melhor compreender e lidar com as famílias assistidas, reduzindo barreiras de acesso e adesão ao sistema de saúde. Será feito um estudo qualitativo de famílias cadastradas pela Estratégia Saúde da Família que atua em uma unidade no município de São Gonçalo-RJ. O acompanhamento acontecerá por pelo menos seis meses, através de visitas domiciliares e entrevistas com os membros das famílias selecionadas. Pretende-se ainda fazer um genograma destas famílias para compreender a estrutura e relações de cada núcleo familiar. Resultados oferecerão subsídios ao Programa "Brasil sem Miséria", através de sugestão de ações que contribuam para mitigar os problemas de saúde nas famílias de baixa renda e para facilitar a adesão aos serviços de saúde.

Auxílio: CAPES, CLAVES.

Participante: **Rosana Therezinha Queiroz de Oliveira**

Título: **51. Tecnologia de avaliação para programas de prevenção e controle de doenças promotoras da pobreza, com ênfase nas parasitoses intestinais**

Autores: Rosana Therezinha Queiroz de Oliveira[1], Martha Macedo de Lima Barata[2], Antonio Henrique Almeida de Moraes Neto[1]

Filiação: [1]Laboratório de Inovações em Terapias, Ensino e Bioprodutos (LITEB), IOC, FIOCRUZ, Rio de Janeiro
[2]Assessoria de Planejamento Estratégico do IOC, Fiocruz, Rio de Janeiro

rosana.therezinha@ioc.fiocruz.br; rosanatherezinha@gmail.com
Palavras-chave: Tecnologia de Avaliação; Programa de Promoção da Saúde; Parasitoses Intestinais.

Segundo a OMS faltam indicadores de avaliação de Programas de Promoção da Saúde (PS). A Matriz PROADESS do Sistema Único de Saúde (SUS) não se aplica a estes. Nosso objetivo é desenvolver matriz de avaliação da efetividade para programas de prevenção e controle das doenças promotoras da pobreza, com ênfase em parasitoses intestinais, aplicável ao Plano *"Brasil Sem Miséria"*(BSM), ao SUS e órgãos de fomento à pesquisa. Esta tecnologia vem sendo desenvolvida em Laje do Muriaé, RJ, através de metodologia participativa, no âmbito do Programa Saúde da Família (n= 71 domicílios). Foram aplicados questionários sobre conhecimentos, atitudes e práticas (CAP) e percepções sobre condições habitacionais e socioambientais do entorno (QHS), em visitas domiciliares, a fim de subsidiar a execução e avaliação de ações em PS para construção de indicadores qualiquantitativos. A maioria dos respondentes do CAP declarou *"repito remédio que o médico passou para evitar vermes"*; 54% julgam que *"os vermes vão pro esgoto depois que saem das pessoas"* e 18% que *"se pega verme comendo doce"*. Todos desconhecem o ciclo de vida dos parasitas. O QHS mostrou que a maioria das famílias apesar do provimento de saneamento básico, não o utiliza. Estes resultados indicam desconhecimento da população e apontam para a necessidade de aplicar PS e estabelecer tecnologia para avaliar sua eficácia e eficiência.

Participante: **Sandro Javier Bedoya Pacheco**

Título: **52. Integração entre epidemiologia e antropologia médica na investigação coletiva das doenças infecciosas relacionadas à pobreza: uma contribuição para o ensino não formal em biociências e saúde.**

Autores: Sandro Javier Bedoya Pacheco[1]; Claudia Teresa Vieira de Souza[2]

Filiação: [1,2] Laboratório de Epidemiologia Clínica do Instituto de Pesquisa Clínica Evandro Chagas/IPEC – Fiocruz, RJ.

sandro.bedoya@ipec.fiocruz.br

Palavras-chave: Epidemiologia social, Antropologia Médica, Ensino não-formal, Promoção da saúde.

A partir do olhar das dimensões sociais envolvidas nos processos de saúde-doença, este trabalho visa discutir de que forma a extrema pobreza compromete a percepção de saúde, o conhecimento da doença e o controle no âmbito de importantes doenças endêmicas infecciosas, além de tentar construir novas propostas de intervenção de ensino não formal em Biociências e Saúde para seu controle. Metodologia: abordagem qualitativa a partir de entrevistas e grupos focais composto de pacientes com Leishmaniose Tegumentar Americana (LTA) e Esporotricose atendidos no IPEC/FIOCRUZ. A metodologia a ser aplicada permitirá organizar e reconhecer, de modo sistemático, o saber local, construindo a partir das experiências de vida dos participantes os principais fatores que influem no processo saúde-doença. Objetivos: Contribuir com o desenvolvimento de instrumentos de investigação coletiva, mais sensíveis, que possam ser utilizados nas práticas de produção de conhecimentos, além de oferecer reflexões teóricas e metodológicas na área de educação em saúde e no estreitamento das ciências sociais e humanas com a epidemiologia, como um apoio ao esforço coletivo de enfrentar as iniquidades em saúde e fundamentar programas de saúde eficazes, coordenados e contínuos. Resultados: Fase de entrevistas (inicio em 01/05/2013)

Auxílio: CAPES, IOC, CNPq, IPEC

Participante: **Sheila Soares de Assis**

Título: **53. Apontamentos e integração de cenários a partir do Programa Saúde na Escola (PSE): contribuições para estratégias envolvendo as doenças negligenciadas e o Plano Brasil Sem Miséria**

Autores: Sheila Soares de Assis[1], Tania Araújo-Jorge[1],

Filiação: [1]Laboratório de Inovações em Terapia, Ensino e Bioprodutos. Instituto Oswaldo Cruz - FIOCRUZ, Rio de Janeiro.

sheila.assis@ioc.fiocruz.br

Palavras-chave: Programa Saúde na escola; Plano Brasil sem Miséria; Doenças Negligenciadas.

Introdução: O Programa Saúde na Escola (PSE) possui o potencial de colaborar na mitigação das doenças negligenciadas (DN) e foi integrado ao Plano Brasil sem Miséria (BSM). A pesquisa se propõe em: (1) Analisar os parâmetros que norteiam as ações educativas do PSE, do BSM e para a abordagem das DN; (2) Investigar a visão dos profissionais do PSE em localidades priorizadas no BSM sobre as ações educativas do programa; (3) Elaborar de forma compartilhada, entre profissionais de saúde e professores materiais educativos relacionados às DN com potencial de emprego nas ações educativas do PSE. **Metodologia:** Para a consecução dos objetivos propostos serão empregadas as metodologias de: (1) análise de documentos, (2) grupo focal e; (3) oficinas em grupos operativos. **Resultados preliminares:** Recuperou-se 209 documentos, sendo 36 relacionados ao PSE, 32 ao BSM e 141 sobre sete DN de alta prevalência nas cinco regiões brasileiras. A análise descritiva destes tem demonstrado um antagonismo na discussão de alguns agravos no PSE. Espera-se que as etapas subsequentes contribuam para a mitigação da pobreza através da prospecção de conhecimentos sobre o PSE e das ações sobre as DN no BSM.

Auxílio: CAPES, IOC.

Participante: **Sidelcina Rugieri Pacheco**

Título: **54. Avaliação da resistência do vírus da hepatite B (VHB) às drogas antivirais utilizadas no tratamento de pacientes com hepatite crônica**

Autora: Sidelcina Rugieri Pacheco

Filiação: Laboratório de Patologia e Biologia Molecular – Centro de Pesquisa Gonçalo Moniz-Fiocruz-Bahia

No Brasil, o tratamento da Hepatite B crônica vem sendo realizado há mais de 10 anos com o uso de diferentes drogas antivirais. O objetivo do estudo foi avaliar marcadores de resistência ao tratamento com análogos

de nucleos(t)ídeos utilizados no tratamento da hepatite B crônica. Os pacientes com hepatite B crônica, atendidos no Ambulatório Magalhães Neto do HUPES-UFBA foram convidados a participar do estudo, mediante assinatura do TCLE e resposta a um questionário clínico-epidemiológico. O HBV-DNA foi amplificado por PCR e sequenciado. Essas sequências foram submetidas ao banco de dados de resistência a drogas HBV para recuperar cada mutação. Durante o período de junho de 2012 a abril de 2013, foram incluídos 111 pacientes. Em relação ao histórico de tratamento, 61 (55%) são virgens, 4 (3,6%) tinham histórico prévio, destes 2 negativaram o VHB, e 46 (41,4%) estão em tratamento. A análise de mutações de resistência foi realizada em um grupo de 51 pacientes. O genótipo com maior prevalência foi A1 (82,4%). Três pacientes (5,9%) apresentaram mutações de resistência à LAM e ENT, com dois padrões L180M e M204V e outro com padrões L80I, L180M e M204I. Uma vez o tratamento iniciado é extremamente importante monitorar a carga viral e identificar as mutações, a fim de apoiar a tomada de decisões clínicas.

Auxilio: Capes;CNPq;Fiocruz

Participante: **Taís Ferreira Gomes**

Título: **55. Investigação dos determinantes da doença de chagas e ações de vigilância e controle em populações-alvo do plano brasil sem miséria na região do vale do jaguaribe, ceará**

Autores: Taís Ferreira Gomes[1], Filipe Aníbal Carvalho-Costa[2] e Marli Maria Lima[1].

Filiação: [1]Laboratório de Ecoepidemiologia da Doença de Chagas/ LEDOC-IOC- FIOCRUZ, Rio de Janeiro.
[2]Laboratório de Sistemática Bioquímica–IOC/FIOCRUZ

tais@ioc.fiocruz.br

Palavras-chave: Doença de Chagas, Triatomíneos, Educação.

Introdução: A doença de Chagas é uma endemia de populações pobres

que vivem em casebres onde os triatomíneos se domiciliam com facilidade. Em localidades rurais e periurbanas do Vale do Jaguaribe Ceará, domicílios, peridomicílios e ambientes naturais registram presença de triatomíneos, frequentemente infectados por *Trypanosoma cruzi*. **Objetivo**: O Projeto propõe investigar os determinantes da doença de Chagas em localidades rurais do município de Russas/CE, buscando ações de controle da endemia chagásica na região. **Métodos**: Serão realizados estudos transversais/observacionais de campo para obtenção de dados demográficos, entomológicos, parasitológicos e epidemiológicos, além de pesquisas qualitativas (estudos de caso) e ações educativas. Proporemos também projetos de melhoria habitacional e do peridomicílio. **Resultados e conclusões**: Até o momento foram visitados 37 domicílios nas comunidades Timbaúba do Pitingão e Riacho do Barro. Metade das casas foi caracterizada como vulnerável. Abordagem qualitativa inicial identificou que a escolha dos métodos de construção dos domicílios é fortemente influenciada por fatores culturais e econômicos, relacionados à propriedade da terra. Casas de pau-a-pique são ainda construídas nas localidades, sendo preferidas por serem consideradas mais seguras por parte da população.

Auxílio: CAPES/BSM/IOC/CNPq

Participante: **Tatiana Figueiredo de Oliveira**

Título: **56. Avaliação da participação pública em um programa de monitoramento biológico para a gestão de águas em um assentamento rural**

Autores: Tatiana Figueiredo de Oliveira[1], Daniel Forsin Buss[1]

Filiação: Laboratório de Avaliação e Promoção da Saúde Ambiental } IOC – FIOCRUZ, Rio de Janeiro

tatibio@ioc.fiocruz.br

Palavras-chave: monitoramento participativo; capital social; percepção de risco; voluntários.

O projeto em questão se aplica ao conceito de tecnologia social, que busca traduzir em benefícios sociais o conhecimento produzido nos setores acadêmicos em locais e territórios específicos, onde possa ser maximizada a interação entre cidadãos, governo e sociedade civil. A partir de programa de monitoramento biológico participativo como tecnologia na gestão de recursos hídricos, e como estratégia de empoderamento de comunidades para que atuem de forma mais efetiva no controle e ação dos impactos sobre os recursos hídricos, o objetivo desta pesquisa é avaliar como a participação de grupos de voluntários neste programa amplia a capacidade de resolução de problemas socioambientais de grupos vulneráveis. Além disso, será criado e testado um instrumento de avaliação da percepção da comunidade sobre os riscos à saúde sobre uso de recursos hídricos disponíveis, além de legitimar uma proposta de gestão para o enfrentamento da resolução de problemas locais. Serão realizados questionários juntamente com entrevistas com os membros do assentamento trabalhado, para avaliar os elementos que compõem o capital social e também para a criação de protocolo de monitoramento de percepção de risco. Até o momento, foi realizado um teste piloto em localidades do Rio de Janeiro, para elaboração de questionário para avaliação da percepção de risco.

Auxílio: CAPES

Participante: **Thatiana Regina Fávaro**

Título**: 57. Evolução do acesso ao saneamento básico no Brasil 2000 – 2010: constrantes em relação a variável raça/cor**.

Autores: Thatiana Fávaro[1], Ludimila Raupp[1], Ricardo Ventura Santos[1,2].

Filiação: [1]Escola Nacional de Saúde Pública- FIOCRUZ, Rio de Janeiro. [2]Departamento de Antropologia do Museu Nacional/UFRJ.

thatifavaro@gmail.com

Palavras-chave: Saneamento básico; Índios sul-americanos; Raça/cor.

Introdução: Diversos segmentos da sociedade brasileira que ainda vivem em situação de miséria, compartilham situação similar de vida, como condições inadequadas de saneamento. O objetivo deste estudo foi analisar evolução da cobertura de serviços de saneamento básico entre 2000 e 2010 evidenciando contrastes e desigualdades entre domicílios com responsáveis indígenas e as demais categorias de raça/cor. Métodos: Com a utilização dos microdados do Censo Demográfico, foram analisadas as condições de saneamento de domicílios localizados na área rural de 120 municípios, selecionados por concentrarem o maior número absoluto de indígenas vivendo em área rural. **Resultados:** Foram observadas diferenças significativas quanto ao acesso a serviços de saneamento entre domicílios com responsáveis indígenas e não indígenas, com piores condições para os indígenas. Apesar disto, os domicílios indígenas, em especial, os localizados nas regiões Norte e Nordeste apresentaram as maiores razões de proporção (RP) para os indicadores analisados apontando maiores avanços na cobertura dos serviços de saneamento no período de 10 anos. Conclusões: Apesar dos indígenas apresentarem condicões menos adequadas de saneamento do que os não indígenas em 2000 e 2010, observa-se que na última década houve um incremento positivo em relação ao estabelecimento de infra-estrutura sanitária nos domicílios indígenas.

Auxílio: CAPES

Participante: **Vanessa Brandão Nardy**

Título: **58. Associação de marcadores genéticos a infecção e/ou desenvolvimento da leishmaniose tegumentar (LT).**

Autores: Vanessa B. Nardy[1], Giséllia S. Santana[1], Ana Angélica Leal Barbosa[2], Jackson Maurício Lopes Costa[1],

Filiação: [1] Laboratório de Imunoparasitologia. CPqGM- FIOCRUZ, Bahia
[2] Laboratório de Biologia Molecular. UESB- Bahia

vanessanardy@gmail.com

Palavras-chave: Leishmaniose Tegumentar, Epidemiologia, TNFs, AIMs.

Introdução: As leishmanioses é uma doença negligenciada que apesar de afetar milhões de indivíduos, seu tratamento é demorado e inadequado, pois as pessoas que sofrem dessas doenças são extremamente pobres não oferecendo um retorno lucrativo para que a indústria farmacêutica invista em pesquisa de novos medicamentos. O Plano Brasil sem miséria vem promover a justiça e a igualdade social na ciência, desmarginalizando tais doenças nos programas de pesquisas. Nosso objetivo é estabelecer o perfil epidemiológico, e caracterizar marcadores moleculares no gene TNF que poderá estar envolvidos na susceptibilidade ou resistência a leishmaniose, além de caracterizar etnicamente a população utilizando marcadores genéticos (AIMs), estas caracterizações são importantes para avaliar o risco populacional para a doenças a qual a amostra está submetida. **Métodos e resultados**: Foi desenvolvido um inquérito epidemiológico, e imunoalérgico com 370 indivíduos, onde foram realizado os IDRMs a coleta de sangue para o exame ELISA e extração do DNA para análise molecular. **Conclusão**: Esse projeto poderá vir contribuir com o plano Brasil sem Miséria visto que a possível associação destes marcadores do TNF e dos de população com esta doença terá grande importância clínica, pois poderá contribuir para prevenção e tratamento, promovendo novos alvos terapêuticos e diagnósticos mais efetivos.

Auxílio: CAPES, IOC, CNPq

Participante: **Wagner Alexandre Costa**

Título: **59. Controle integrado da Leishmaniose Tegumentar Americana em comunidades impactadas pelas obras do Programa de Aceleração do Crescimento (PAC), em Jacarepaguá, Rio de Janeiro**

Autores: Wagner A. Costa1, Tania Araújo-Jorge 4, Angela Ostritz 3, José Luís Cordeiro 3, Julia Lins1, Antonio Santana1, Elizabeth Rangel1

1Lab. Transmissores de Leishmanioses e 2 Lab. de Inovações em Terapias, Ensino e Bioprodutos, Instituto Oswaldo Cruz;3 Campus Fiocruz da Mata Atlântica.

wagnerac@ioc.fiocruz.br

A Leishmaniose Tegumentar Americana (LTA) prevalece em condições de pobreza sustentando um quadro de desigualdades. Relevante e complexa epidemiologicamente, tem se disseminado segundo condicionantes ambientais, históricos e sociopolíticos. No Rio de Janeiro, o registro de casos aumenta desde os anos 80, principalmente na zona oeste (Jacarepaguá), onde está o Campus Fiocruz Mata Atlântica (CFMA). A LTA atinge diversas idades sem distinção de sexo, com transmissão intra e peridomiciliar. OBJETIVOS: acompanhar a transmissão de LTA, avaliando impactos das obras do PAC na dinâmica de flebotomíneos; trabalhar indicadores ambientais relacionados à vigilância de vetores; mapear o perfil socioeconômico dos moradores e correlacionar com a ocorrência de LTA. O CFMA compreende uma área de resíduo de Mata Atlântica, 45% em zona residencial. Estações de Monitoramento Entomológico foram estabelecidas nas 3 comunidades, com histórico de maior concentração de casos humanos de LTA, e que serão as mais impactadas pelas obras do PAC. Nossos estudos iniciais revelaram que as comunidades do CFMA apresentam os mais baixos índices sociais e econômicos da região, somados às características ambientais, favorecendo a manutenção da transmissão da LTA.

Financiamento: Convênio FIOCRUZ-SVS e POM/ IOC; Programa Brasil Sem Miséria
