# Supplementary material for: For socially engaged science: The dynamics of knowledge production in the Fiocruz graduate program in the framework of the "Brazil Without Extreme Poverty Plan"
Source: PLoS One. 2018 Oct 19;13(10):e0204232. doi: 10.1371/journal.pone.0204232 (PMC6195260; doi:10.1371/journal.pone.0204232)
Supplement: S2 File — Reference document for the First BWEP meeting (Portuguese). (PDF) [file pone.0204232.s002.pdf]

ANÁLISE DAS PESQUISAS DE DOUTORADO E PÓS-DOUTORADO DA FIOCRUZ NO ÂMBITO DO PLANO BRASIL SEM MISÉRIA

Rebeca Buzzo Feltrin[1]

Apresentação

O presente documento analisa os avanços empreendidos no desenvolvimento das pesquisas de doutorado e pós-doutorado da Fundação Oswaldo Cruz (Fiocruz) no âmbito do plano Brasil Sem Miséria (BSM), visando atingir maior eficiência no cumprimento dos objetivos propostos no Acordo de Cooperação Técnica firmado entre a Fiocruz e o Ministério do Desenvolvimento Social e Combate à Fome (MDS).

1. O Plano Brasil Sem Miséria

O Plano Brasil Sem Miséria é um programa governamental voltado a erradicação da extrema pobreza no Brasil, buscando estratégias para a elevação da renda e bem-estar da população em situação de miséria, além da inclusão desse público em programas do governo a fim de atender suas necessidades (Brasil, 2011[2]; Fiocruz, 2014[3]). O programa se organiza em três eixos principais: 1) garantia de renda: voltado para o alívio imediato da situação de extrema pobreza dessas populações; 2) acesso a serviços públicos: destinado a melhoraria das condições de educação, saúde e cidadania das famílias em situação de extrema pobreza no Brasil e 3) inclusão produtiva: orientado a aumentar a capacidade, oportunidades de trabalho e geração de renda entre o público alvo, no campo e nas cidades (Brasil, 2014)[4].

A Fiocruz aderiu ao plano BSM no cumprimento dos seus objetivos, apoiando o desenvolvimento de projetos de pesquisa que contribuem para a superação de problemas voltados ao público-alvo do programa (Fiocruz, 2014). A Nota Técnica NT 01/2011-IOC (Fiocruz, 2011)[5], elaborada pelo Instituto Oswaldo Cruz (IOC), tornou-se um marco da

---

1 Pós-doutoranda da Fiocruz (VPEIC) - convênio CAPES/BSM.
[2] BRASIL, 2011. DECRETO Nº 7.492, DE 2 DE JUNHO DE 2011. Disponível em: <http://www.planalto.gov.br/ccivil_03/_Ato2011-2014/2011/Decreto/D7492.htm>
[3] FIOCRUZ, 2014. Disponível em: <http://www.fiocruz.br/ioc/cgi/cgilua.exe/sys/start.htm?sid=331>
[4] BRASIL, 2014. Disponível em: <http://www.brasilsemmiseria.gov.br/apresentacao>
[5] FIOCRUZ, 2011. Nota Técnica N.º 1/2011/IOC-FIOCRUZ/DIRETORIA. Disponível em: <http://www.fiocruz.br/ioc/media/NotaTecnica_1_2011_IOCatual.pdf>

participação da Fiocruz no Plano BSM, tendo como principal recomendação a inclusão da temática "doenças da pobreza" nos objetivos do programa. Após o lançamento da Norma Técnica, outras questões referentes à Saúde, Ciência e Educação também passaram a ser integradas ao Plano BSM.

Neste contexto, o Acordo de Cooperação Técnica firmado entre a CAPES, Fiocruz e o Ministério do Desenvolvimento Social e Combate à Fome, estabeleceu as ações prioritárias das pesquisas desenvolvidas pela Fiocruz no âmbito do BSM:

> a) Estimular a geração de conhecimentos voltados para a mitigação de problemas relacionados à extrema pobreza;
> b) Articular a geração de conhecimentos com a elaboração de propostas de aplicação de tecnologias biomédicas, sociais e educacionais capazes de atender o público do Plano Brasil Sem Miséria, com especial atenção a temas relacionados a: (i) doenças perpetuadoras da pobreza; (ii) ambiente, saúde e pobreza; (iii) educação, saúde e pobreza; (iv) cultura, saúde e pobreza; e (v) atenção materno-infantil;
> c) Promover processos formativos voltados para a qualificação de agentes públicos e sociais que atuam nas políticas, programas e ações no âmbito do Plano Brasil sem Miséria (Fiocruz, 2014, s/n).

O convênio 0123/2011 (de 18 de novembro de 2011) definiu a cooperação através de bolsas especiais de doutorado e pós-doutorado, destinadas ao desenvolvimento de projetos dentro das temáticas do Plano Brasil Sem Miséria, entre os anos de 2012 e 2017. O Acordo de Cooperação prevê ainda ações para a qualificação de agentes públicos e sociais que atuam nas políticas/programas e/ou ações voltadas ao público-alvo do BSM (Brasil, 2014).

2. Atuação da Fiocruz no Plano BSM

Em 2012, a Fiocruz foi contemplada com 100 bolsas especiais de doutorado e 25 de pós-doutorado para o desenvolvimento de pesquisas voltadas às temáticas do Plano Brasil Sem Miséria - BSM (Fiocruz, 2013)[6].

Atualmente, a Fiocruz conta com 113 participantes (bolsistas e não bolsistas) responsáveis pela produção de conhecimento dentro das temáticas do BSM, sendo 87 de doutorado e 26 de pós-doutorado, além de outros pesquisadores envolvidos na temática

[6] FIOCRUZ, 2013. Caderno de Resumos – Seminário: A Pós-graduação na Fiocruz e o Plano Brasil sem Miséria.

que não recebem auxílio financeiro do programa. A partir do levantamento desses trabalhos - reunidos no Caderno de Resumos do Seminário “A pós-graduação na Fiocruz e o Plano Brasil sem Miséria” (Fiocruz, 2013) - foi possível identificar o enfoque das pesquisas desenvolvidas no âmbito do convênio. O mapeamento das pesquisas em curso (bolsistas e não bolsistas) permitiu identificar seis “eixos temáticos” principais, conforme apresentados a seguir:

1 – Controle, monitoramento e tratamento de doenças relacionadas à pobreza: essa temática reúne estudos sobre o controle, monitoramento e tratamento de doenças nas populações alvo do BSM. Abrange pesquisas laboratoriais, desenvolvimento de fármacos e vacinas para o tratamento dessas doenças. Também inclui metodologias para o controle e monitoramento das doenças, como sequenciamento genético e métodos de biologia molecular.

2 – Aspectos epidemiológicos e estratégias de diagnóstico de doenças: a temática abrange trabalhos voltados aos aspectos epidemiológicos do processo saúde/doença, ou seja, a distribuição e relação de causalidade da saúde ou doença em populações específicas. As principais doenças estudadas nessa linha incluem doença de Chagas, dengue, malária, hepatite, hanseníase, tuberculose, leishmaniose, leptospirose, entre outras doenças associadas à situação de pobreza. Os estudos nessa abordagem exigem uma interação interdisciplinar, tendo em vista que os fatores determinantes para a ocorrência das doenças estão ligados tanto a fenômenos biológicos como sociais. As estratégias de diagnóstico das doenças também são consideradas dentro da temática, uma vez que se utilizam de métodos epidemiológicos para identificar a ocorrência de doenças em determinadas populações.

3 – Populações em situação de risco: os trabalhos dentro dessa temática estão direcionados ao estudo de populações expostas a riscos de diversas naturezas: ambientais, dificuldades de acesso aos serviços públicos básicos (saneamento básico, habitação, saúde, etc.), maior susceptibilidade para contrair certas doenças. As populações vulneráveis estudadas nessa linha incluem os moradores

de comunidades, indígenas, populações ribeirinhas, além de moradores de áreas endêmicas.

4 – Ações educativas em saúde: a temática reúne pesquisas sobre ações/práticas educativas em saúde das populações alvo do BSM, com o objetivo principal de informar a população sobre estratégias de combate às doenças. Dentre as principais ações destacamos o desenvolvimento de material didático/campanhas sobre transmissão e controle de doenças, inclusive, com utilização das Tecnologias de Informação e Comunicação (TICS), além de outras ações voltadas à divulgação científica.

5 – Avaliação de políticas públicas: os trabalhos nessa temática procuram avaliar os efeitos/resultados dos programas e políticas públicas direcionados às populações beneficiárias do BSM. Os principais programas/políticas analisados incluem o Brasil Sem Miséria, o Programa Bolsa Família, Programa de Aceleração do Crescimento, entre outros programas do Governo Federal.

6 – Relações entre Ciência, Tecnologia e Sociedade: os trabalhos dentro dessa temática procuram investigar as relações entre o desenvolvimento científico e tecnológico produzido e sua interação com a sociedade, especialmente, envolvendo as demandas das populações alvo do BSM. Procuram também analisar o desenvolvimento de tecnologias sociais, as relações entre conhecimento tradicional e científico, inclusão social no ensino e pesquisa, história da ciência, bem como, a participação pública na C&T.

É importante ressaltar que muitos trabalhos poderiam estar enquadrados em dois ou mais eixos temáticos simultaneamente, devido à sua natureza interdisciplinar. Entretanto, para facilitar o mapeamento dos temas, buscou-se categorizar cada trabalho de acordo com seu eixo temático principal, conforme apresentado nos Gráficos 1 e 2:

Gráfico 1 - Distribuição de pesquisas do BSM por Eixo Temático

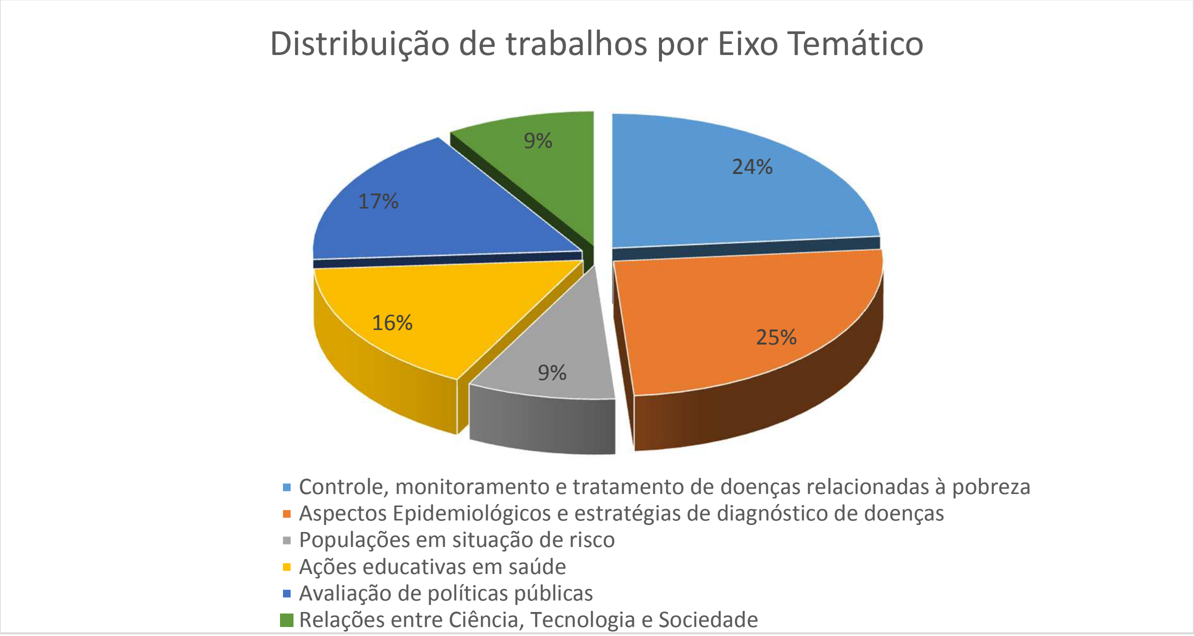

Gráfico 2 - Distribuição das pesquisas BSM por eixo temático e nível acadêmico

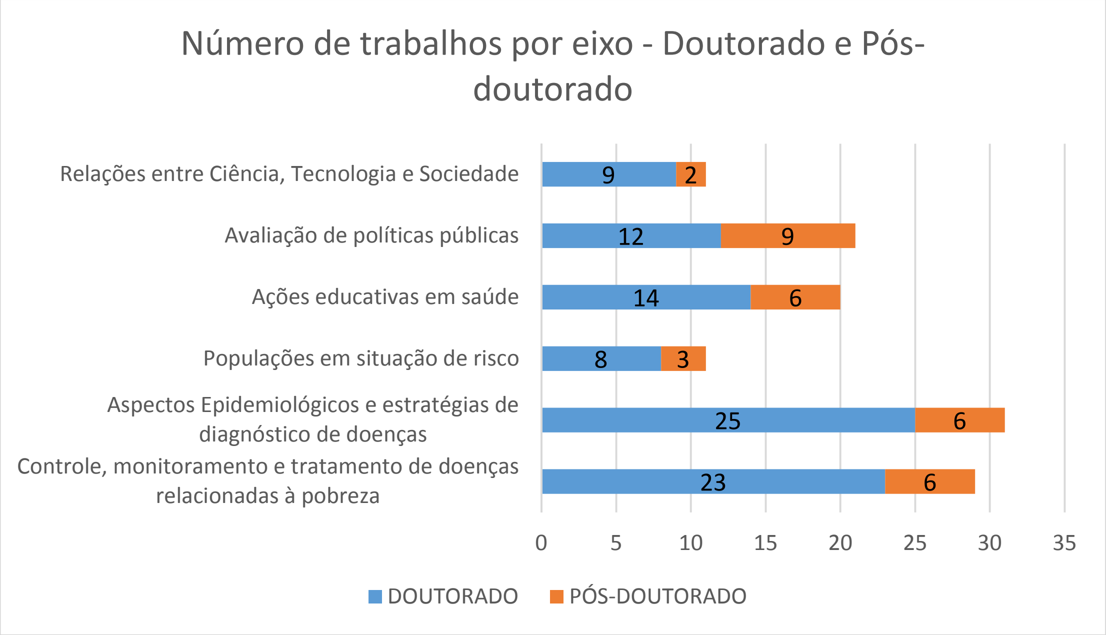

Os temas abordados cumprem o objetivo geral proposto no Acordo de Cooperação Técnica, que propõe gerar conhecimentos voltados à solução de problemas relacionados à extrema pobreza. Entretanto, a grande maioria das pesquisas se concentra em temas relacionados a "doenças da pobreza" (49%) - dividas nos eixos temáticos "Controle, monitoramento e tratamento de doenças relacionadas à pobreza" e "Aspectos

Epidemiológicos e estratégias de diagnóstico de doenças". Assim, outros temas considerados estratégicos previstos na Chamada para estágio pós-doutoral do convênio CAPES/BSM (CAPES, 2011)[7] acabaram sub-representados. Como exemplo, as temáticas relacionadas à "cultura, saúde mental e pobreza"; "atenção materno-infantil"; "inclusão social através da ciência" ou "pesquisas voltadas ao apoio das populações em situação de rua" tiveram pouca ou nenhuma abordagem até o momento, demostrando a necessidade de estimular pesquisas que desenvolvam tais temas.

Além disso, nota-se uma repetição de temas dentro de cada eixo temático – no caso do eixo "Avaliação de Políticas Públicas", por exemplo, os trabalhos concentram-se na avaliação de programas como o Bolsa Família e Saúde na Escola, sendo que outros programas tidos como prioritários do BSM não são sequer mencionados entre as pesquisas, como por exemplo, os Programas Mulheres Mil e Pronatec (voltados à população entre 18 e 59 anos).

3. Distribuição das pesquisas por Unidade

De acordo com o levantamento realizado pela Coordenação Geral de Pós-Graduação (CGPG) entre outubro/2013 e maio/2014, os pesquisadores de doutorado e pós-doutorado vinculados ao BSM estão alocados entre dez unidades da Fiocruz, localizadas nas seguintes capitais brasileiras: Rio de Janeiro, Recife, Salvador, Belo Horizonte e Curitiba.

[7] CAPES, 2011. Chamada para Estágio Pós Doutoral - Projetos de pesquisa em apoio à eliminação da pobreza extrema no Brasil. Rio de Janeiro, Setembro-2011.

Tabela 1- Distribuição dos pesquisadores (plano BSM) por Unidade da Fiocruz

Acordo Capes/Fiocruz - Plano Brasil Sem Miséria								
	Doutorandos			Pós-doutorandos			Total de participantes (D + PD)	Total de participantes (D + PD) %
Unidades	Participantes	Bolsistas	Não Bolsistas	Participantes	Bolsistas	Não Bolsistas		
COC	2	2	0	0	0	0	2	2%
CPqAM (Recife)	8	8	0	2	2	0	10	9%
CPqGM (Salvador)	9	9	0	1	1	0	10	9%
CPqRR (Belo Horizonte)	0	0	0	1	0	1	1	11%
ENSP	6	6	0	9	7	2	15	13%
ICC (Curitiba)	7	7	0	0	0	0	7	6%
ICICT	6	4	2	0	0	0	6	7%
IOC	50	43	7	13	12	1	63	55%
CGPG/VPEIC	0	0	0	0	0	0	0	0%
DIREB	0	0	0	1	1	0	1	1%
**TOTAL**	**88**	**79**	**9**	**27**	**23**	**4**	**115**	**100%**

Fonte: CGPG/Fiocruz (2014)

A partir da Tabela 1, podemos observar uma expressiva concentração de participantes (55%) desenvolvendo seus trabalhos no IOC. Tendo em vista que a Nota Técnica do IOC em 2011 inaugurou a parceria da Fiocruz com o MDS no Plano Brasil Sem Miséria, justifica-se a concentração de pesquisas BSM nesse instituto, bem como, a temática prevalente entre os trabalhos relacionados às doenças da pobreza. Entretanto, vale destacar os esforços posteriores da instituição em colocar em pauta temáticas relacionadas à Saúde, Ciência e Educação, as quais podem ser consideradas estratégicas para o BSM, tendo em vista o caráter social do plano.

Nota-se ainda que algumas unidades que possuem Programas de Pós-Graduação (PPG) não possuem participantes (bolsistas ou não) vinculados ao BSM, como é o caso das unidades: IFF, INCQS e INI. A área controle de qualidade, de doenças infecciosas, e da Saúde da Mulher, da Criança e do Adolescente apresenta inúmeras oportunidades temas ainda não foram abordados pelos pesquisadores do BSM.

4. Estratégias para integração/ampliação das pesquisas e ações vinculadas ao BSM

Com o objetivo de integrar, fortalecer e ampliar as pesquisas e ações desenvolvidas na Fiocruz através do convênio com o plano Brasil sem Miséria, recomenda-se as seguintes estratégias:

1- **Edital Fiocruz/BSM:** recomenda-se a organização de um edital visando selecionar pesquisas de pós-doutorado com foco nas temáticas que carecem de maior aprofundamento e/ou que não foram abordadas pelas pesquisas vinculadas ao BSM até o momento, especialmente, a serem desenvolvidas em unidades da Fiocruz que ainda não contam com participantes no BSM.
2- **Migração ao convênio CAPES/BSM de pesquisas em desenvolvimento:** Indicação por parte dos docentes e/ou dos próprios pós-doutorandos de pesquisas em desenvolvimento na Fiocruz que possam ser migradas ao BSM.
3- **Encontros periódicos dentro de cada eixo temático:** a partir do levantamento das temáticas das pesquisas em desenvolvimento, propõe-se formar grupos de trabalho (GT) para cooperação, aproximando pesquisas de doutorado e pós-doutorado em temas afins e com uma abordagem interdisciplinar.
4- **Redes de especialistas:** mapear as redes de especialistas no Brasil e exterior com as quais a Fiocruz tem relações ou que pretende firmar parcerias em áreas relevantes ao BSM. A identificação dessas redes possibilitará ainda futuros intercâmbios entre pesquisadores e/ou docentes do BSM, além de a organização de conferências e palestras com representantes de grupos de pesquisa que possam contribuir com a geração de conhecimento para erradicação da pobreza.
5- **Cooperação Técnica Internacional:** recomenda-se estimular o intercâmbio de pesquisadores no BSM entre centros de pesquisa de referência mundial em sua área de atuação, buscando cooperação no desenvolvimento de pesquisas, possibilitar o contato com novas tecnologias e referenciais teóricos/metodológicos que possam ser aplicados à realidade brasileira, visando ainda auxiliar na erradicação da miséria e doenças prevalentes entre populações em situação de extrema pobreza. Além disso, o intercâmbio de pesquisadores poderá ainda auxiliar na ampla divulgação científica resultados alcançados no âmbito do BSM.

6- **Ação integrada aos observatórios da Fiocruz de determinantes sociais da saúde, pesquisa e desenvolvimento tecnológico:** Levantamento de indicadores (em momentos a serem definidos) para acompanhar o desenvolvimento de pesquisas BSM e o impacto de seus resultados na produção científica, impacto social e contribuições para a formulação, fortalecimento e/ou avaliação de políticas públicas.
7- **Inclusão social na Fiocruz:** propor ações que possibilitem o acesso e permanência do público-alvo do BSM nos cursos de formação profissional da Fiocruz, especialmente, na Rede de Escolas Técnicas do SUS – RET-SUS[8]. Tal proposta vem de encontro com a demanda do BSM em qualificar pessoas em situação de extrema pobreza com o objetivo de gerar emprego e renda.
8- **Pesquisas orientadas ao desenvolvimento de produtos/propostas ou processos para o BSM:** estimular as pesquisas de doutorado e pós-doutorado do BSM para o desenvolvimento de produtos, propostas ou processos que possam auxiliar no cumprimento dos objetivos de erradicação da pobreza e aumento do bem-estar das populações-alvo do programa, ou ainda subsidiar a avaliação de programas ou ações voltadas a esse fim (Fiocruz, 2011).

5. Interseccionalidade, Interdisciplinaridade e Integração dos saberes

Buscando aprimorar as pesquisas em desenvolvimento, são apresentadas nessa seção recomendações direcionadas aos doutorandos e pós-doutorandos vinculados ao BSM. As recomendações estão pautadas em três conceitos fundamentais: interseccionalidade, interdisciplinaridade e integração dos saberes.

Em primeiro lugar, o conceito de *Interseccionalidade* (McCall, 2005)[9] deve ser considerado, especialmente quando se estuda grupos em situação de desigualdade. Quando consideramos o público-alvo do BSM como um grupo homogêneo, como se todos fossem iguais por conta de sua condição socioeconômica, desconsideramos questões fundamentais que poderiam auxiliar os indivíduos na superação da miséria.

---

8 Disponível em: < http://www.retsus.fiocruz.br/>
9 McCALL, Leslie. 2005. The complexity of intersectionality. *Signs: Journal of Women in Culture and Society.* Vol. 30, n.3: 1771-1800.

Deve-se considerar as particularidades dentro desses grupos, como as questões de raça/cor/etnia, aspectos geracionais e de gênero, por exemplo. Esses marcadores sociais de diferença podem ou não se traduzir em desigualdades sociais. Além disso, quando combinados, essas diferenças podem gerar outros significados ou ainda outros tipos de desigualdade. Por exemplo, as estratégias para retirar da extrema pobreza ou aumentar o nível de bem-estar devem se adaptar às particularidades dos indivíduos: uma mulher, chefe de família, idosa, branca e alfabetizada tem necessidades diferentes de um homem, jovem, negro e analfabeto, embora ambos estejam em situação de extrema pobreza. Não se trata de "somar" desigualdades, mas de ser sensível a como esses marcadores sociais operam de forma diferente em cada situação. Essa ideia deve ser reforçada nas pesquisas voltadas ao público do BSM, uma vez que não se pode assumir que a necessidade de todos são as mesmas baseado apenas em seu status socioeconômico.

A *Interdisciplinaridade* é também um elemento crucial para o desenvolvimento das pesquisas vinculadas ao BSM. A oportunidade de abordar determinados problemas sob a ótica de disciplinas diferentes possibilita o encontro de novas soluções, transpondo barreiras e ampliando os limites do conhecimento. Os desafios colocados pelo BSM estão sendo analisados sob diferentes abordagens, em diferentes unidades e áreas do conhecimento. A ideia de aproximar os trabalhos por Eixo Temático e não separados por departamentos ou PPG, permitirá o estabelecimento de Grupos de Trabalho interdisciplinares orientados à resolução dos problemas sob pontos de vista diferenciados.

Outro ponto a ser considerado é a *Integração dos Saberes*, que se constitui como a aproximação e intercâmbio dos saberes científicos com os conhecimentos produzidos pela sociedade. A teoria do sociólogo Boaventura de Souza Santos (2004)[10] que propõe uma Ecologia dos Saberes é utilizada como base para a compreensão do conceito. A ciência, como forma de conhecimento legitimada em nossa sociedade, se sente capaz de legislar sobre as outras formas de conhecimento, rotulando-as como hierarquicamente inferior. Entretanto, a Ecologia dos Saberes propõe uma interação entre os diversos tipos de conhecimento, considerando que todos os saberes (incluindo o científico) podem se enriquecer através desse diálogo. O autor refere-se a esse movimento como uma revolução epistemológica no seio da universidade, permitindo a participação do público na definição e execução dos projetos de pesquisa.

---

[10] Santos, B.S. de. (2004). A *universidade no século XXI*: *para uma reforma democrática e emancipatória da Universidade*. São Paulo: Cortez.

Assim, deve-se considerar que o conhecimento produzido pelos grupos locais, embora não sigam um “método científico”, muitas vezes se traduzem em soluções eficazes para a melhoria dos problemas do cotidiano dessas pessoas. Da mesma forma, muitos conhecimentos científicos, por desconsiderarem aspectos locais, acabam sendo ineficazes quando aplicados em determinados contextos.

Os movimentos sociais organizados que tentam conscientizar os moradores de sua comunidade sobre assuntos como saúde e educação, que criam iniciativas culturais para melhoria de vida dessas pessoas, que buscam soluções para problemas da comunidade junto ao poder público, os indivíduos que criam soluções caseiras para evitar/combater pragas, entre outros exemplos, devem ser altamente valorizados pelos pesquisadores do BSM, especialmente quando tratamos desses grupos em situação de extrema pobreza que, por serem marginalizados e excluídos das políticas e serviços públicos, acabam encontrando sozinhos inúmeras maneiras de sobreviverem à miséria.

Considerações Finais

A partir da análise dos documentos referentes à parceria entre a Fiocruz e MDS no contexto de geração de conhecimento para o Brasil Sem Miséria, bem como, do levantamento das pesquisas de doutorado e pós-doutorado em desenvolvimento vinculadas ao Plano, percebeu-se a necessidade de criar estratégias para otimizar o cumprimento dos objetivos propostos no Acordo de Cooperação Técnica. Tais estratégias versam sobre uma maior integração das pesquisas BSM com temáticas afins, estímulo ao desenvolvimento de temas que ainda não foram contempladas por essas pesquisas, mapeamento de grupos de especialistas no Brasil e exterior que possam contribuir para a solução de problemas alvo do BSM, ações para inclusão social na Fiocruz, melhoria das ferramentas de acompanhamento e gestão do Plano na Fiocruz (integração ao Observatório), além do estímulo às pesquisas BSM para formularem soluções (produtos/processos/propostas) que atendam às necessidades do público alvo do BSM. Por fim, oferece recomendações aos doutorandos e pós-doutorandos do BSM para desenvolvimento das pesquisas pautados nos conceitos de interseccionalidade, interdisciplinaridade e integração dos saberes.
