# Supplementary material for: For socially engaged science: The dynamics of knowledge production in the Fiocruz graduate program in the framework of the "Brazil Without Extreme Poverty Plan"
Source: PLoS One. 2018 Oct 19;13(10):e0204232. doi: 10.1371/journal.pone.0204232 (PMC6195260; doi:10.1371/journal.pone.0204232)
Supplement: S3 File — Analysis of the survey applied to post-doctoral students and the Brazil without Extreme Poverty Plan (Portuguese). (PDF) [file pone.0204232.s003.pdf]

Ministério da Saúde

FIOCRUZ
Fundação Oswaldo Cruz

PLANO BRASIL SEM MISÉRIA

ANÁLISE DOS RESULTADOS DO FORMULÁRIO DE ACOMPANHAMENTO DAS PESQUISAS ALINHADAS AO BRASIL SEM MISÉRIA

Rebeca Buzzo Feltrin[1]

APRESENTAÇÃO 2
PARTE I - ORGANIZAÇÃO DO FORMULÁRIO 3
EIXOS TEMÁTICOS 3
COLABORAÇÃO 7
POPULAÇÕES BENEFICIÁRIAS DA PESQUISA 8
PRODUTOS, PROCESSOS OU PROPOSTAS DE CARÁTER SOCIAL 9
CRÍTICAS, SUGESTÕES, DÚVIDAS DOS PESQUISADORES ORGANIZADAS POR TEMA . 10
PARTE II - RESULTADOS PRELIMINARES DAS ESTRATÉGIAS PARA A INTEGRAÇÃO DAS PESQUISAS BSM 11
AÇÕES PARA O ATENDIMENTO DAS DEMANDAS DOS PESQUISADORES E CUMPRIMENTO DAS ESTRATÉGIAS 13

[1] Pós-doutoranda da Fiocruz (VPEIC) - convênio CAPES/BSM.

APRESENTAÇÃO

O documento denominado "Análise das Pesquisas de Doutorado e Pós-doutorado da Fiocruz no Âmbito do Plano Brasil Sem Miséria[2]", utilizado como referência para a reunião de gestores no dia 13 de novembro de 2014, apresentou o mapeamento das teses e pesquisas de pós-doutorado do BSM organizadas por eixos temáticos, a fim de gerar subsídios para a avaliação do convênio na Fiocruz. Nesse sentido, o documento apresentou algumas estratégias preliminares buscando maior eficiência no cumprimento dos objetivos propostos no Acordo de Cooperação Técnica firmado entre a Fiocruz e o Ministério do Desenvolvimento Social e Combate à Fome (MDS). Os resultados obtidos na análise preliminar e as estratégias propostas no documento foram discutidas na reunião dos gestores.

A partir dessa primeira análise, observou-se a necessidade de elaborar um formulário direcionado aos alunos integrados ao BSM para que tivessem a oportunidade de indicar o eixo temático que mais se aproximasse de sua pesquisa, além de exprimirem sua opinião sobre o desenvolvimento dos projetos ligados ao programa. A aplicação do formulário permitiu dar voz aos pesquisadores envolvidos, contribuindo para uma avaliação mais fiel dos resultados do convênio e validação das estratégias propostas.

O levantamento realizado em Fevereiro/2015 apontou que a Fiocruz conta atualmente com 114 participantes (bolsistas e não bolsistas) responsáveis pela produção de conhecimento dentro das temáticas do BSM em nível de doutorado e pós-doutorado. No total, 110 alunos (105 bolsistas e 5 não bolsistas) responderam ao formulário de acompanhamento do BSM. Na primeira parte do presente relatório serão apresentados os resultados do formulário quanto ao enquadramento das pesquisas em eixos temáticos, colaboradores atuais e potenciais, dúvidas/críticas e sugestões, populações beneficiárias, produtos/processos derivados das pesquisas, além das unidades da Fiocruz onde estão alocados os pesquisadores. Na segunda seção desse documento, serão apresentados os resultados preliminares da aplicação das estratégias para integração das pesquisas BSM, os resultados do Edital de seleção dos pós-doutorandos, além de uma lista de ações propostas para atendimento às demandas levantadas pelos pesquisadores através do formulário aplicado.

---

2 Análise das Pesquisas de Doutorado e Pós-doutorado da Fiocruz no Âmbito do Plano Brasil Sem Miséria. Elaborado por Rebeca Buzzo Feltrin. Novembro, 2014.

PARTE I - ORGANIZAÇÃO DO FORMULÁRIO

O formulário de acompanhamento do convênio foi aplicado aos pesquisadores participantes do BSM entre Dezembro/2014 e Fevereiro/2015. No total, foram organizadas 14 questões, incluindo questões abertas e fechadas sobre as pesquisas em desenvolvimento. Oito questões do formulário foram destinadas à identificação do aluno e da pesquisa em desenvolvimento - nome, título, unidade da Fiocruz, se tem bolsa BSM, nível (doutorado ou pós-doutorado), resumo, além da data de início e término da pesquisa. Essa seção de identificação permitiu atualizar as informações sobre as pesquisas BSM em andamento, além de contribuir para a organização do Caderno de Resumos do próximo evento. A segunda parte do formulário trouxe questões sobre o enquadramento da pesquisa em um eixo temático principal, a indicação dos produtos/processos ou propostas decorrentes da pesquisa, a população beneficiária, além de três questões abertas relacionadas à colaboração atual da pesquisa, colaborações potenciais e um espaço para críticas, dúvidas e sugestões sobre o convênio. Os resultados do formulário foram analisados e serão discutidos a seguir.

EIXOS TEMÁTICOS

A partir do levantamento dos resumos das pesquisas BSM reunidos no Caderno de Resumos do Seminário "A pós-graduação na Fiocruz e o Plano Brasil sem Miséria" (Fiocruz, 2013) foram identificados seis "eixos temáticos" principais, replicados a seguir:

1 – Controle, monitoramento e tratamento de doenças relacionadas à pobreza

2 – Aspectos epidemiológicos e estratégias de diagnóstico de doenças

3 – Populações em situação de risco

4 – Ações educativas em saúde

5 – Avaliação de políticas públicas

6 – Relações entre Ciência, Tecnologia e Sociedade

Conforme discutido no documento "Análise das Pesquisas de Doutorado e Pós-doutorado da Fiocruz no Âmbito do Plano Brasil Sem Miséria", muitos trabalhos poderiam estar enquadrados em dois ou mais eixos temáticos simultaneamente, devido à sua natureza interdisciplinar. Entretanto, para facilitar o mapeamento dos temas, buscou-

se categorizar cada trabalho de acordo com seu eixo temático principal. Nessa segunda fase de análise, os pesquisadores tiveram a oportunidade de indicarem o eixo temático que mais se aproximasse de sua própria pesquisa, através do formulário aplicado. Os gráficos a seguir apresentam a distribuição das pesquisas por eixo temático principal (Gráfico 1) e as pesquisas de doutorado e pós-doutorado BSM categorizadas pelo eixo temático declarado pelos próprios pesquisadores (Gráfico 2):

Gráfico 1- Distribuição dos trabalhos por eixos temáticos (conforme declarado pelos pesquisadores)

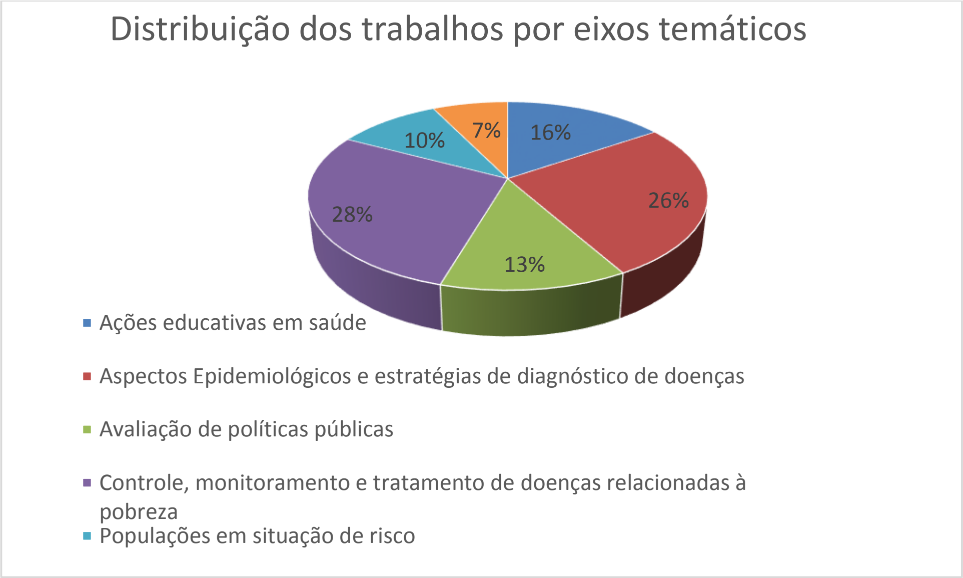

Gráfico 2- Pesquisas de doutorado e PD, por eixos temáticos declarados pelos pesquisadores

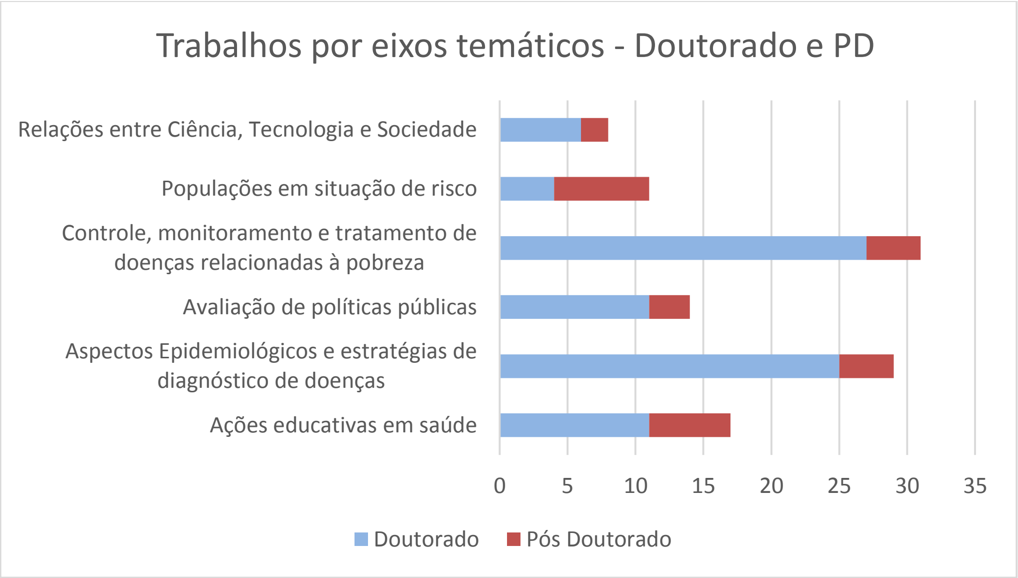

Assim como na análise realizada em 2014, houve uma concentração das pesquisas BSM em temas relacionados às “doenças da pobreza” - dividas nos eixos temáticos “Controle, monitoramento e tratamento de doenças relacionadas à pobreza” e “Aspectos Epidemiológicos e estratégias de diagnóstico de doenças”. Mais do que isso, houve um aumento no percentual de pesquisas nesses eixos combinados (de 49% para 54%), com a diminuição do percentual de pesquisas em eixos temáticos estratégicos como “Ações Educativas em Saúde”, “Avaliação de Políticas Públicas” e “Relações entre Ciência, Tecnologia e Sociedade”. Embora o número de pesquisas analisadas no período seja diferente (123 na primeira análise e 110 na segunda) - tendo em vista que muitas pesquisas classificadas no primeiro documento já foram finalizadas - a distribuição percentual dentro de algumas temáticas diminuiu em detrimento de outras temáticas predominantes.

A possibilidade de identificação do eixo temático principal pelos próprios pesquisadores também contribuiu para essa mudança, proporcionando a realocação de suas pesquisas. Isso pode ser atribuído tanto ao fato de algumas pesquisas terem passado por um processo natural de mudança de percurso entre o período de 2013 (primeira análise baseada no Caderno de Resumos - 2013) a fevereiro/2015 (segunda análise baseada na autodeclaração dos participantes), quanto pela preferência do próprio pesquisador/autor que identificou sua pesquisa dentro de um eixo temático principal diferente daquele estabelecido na primeira análise. Conforme discutido anteriormente, a própria interdisciplinaridade das pesquisas do BSM possibilita enquadramentos em diferentes temáticas. O Gráfico 3 apresenta a distribuição por eixos temáticos comparando esses dois momentos de análise:

Gráfico 3- Distribuição dos trabalhos por eixos temáticos - Comparativo (2013 e 2015)

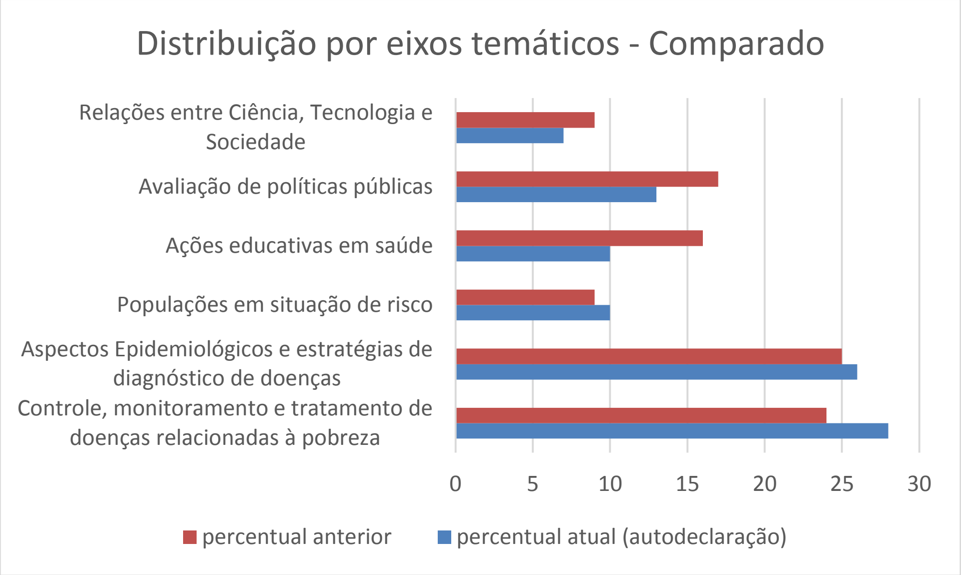

Como estratégia para estimular pesquisas em temáticas pouco exploradas no BSM foi identificada a necessidade de priorizar, através do Edital de Pós-doutorado, a seleção de bolsistas cujas pesquisas fossem alinhadas a tais eixos temáticos.

Da mesma forma, a primeira análise sobre a distribuição das pesquisas BSM por unidades da Fiocruz demonstrou que algumas unidades não tinham sequer uma pesquisa dentro do convênio, sendo necessário estimular através do Edital de Pós-Doutorado o desenvolvimento de pesquisas nesses locais.

Após a aplicação do formulário e atualização dos dados das pesquisas BSM em desenvolvimento, pode-se observar a nova distribuição das pesquisas por unidades da Fiocruz. Muitas pesquisas recentes atreladas ao BSM não constavam no Caderno de Resumos (Fiocruz, 2013) e puderam ser incluídas nessa fase, conforme Tabela 1:

Tabela 1- *Distribuição das Pesquisas BSM por Unidade*

Unidade	Modalidade		Total
	Doutorado	Pós Doutorado	
COC	2		2
CPqAM (Recife)	7	2	9
CPqGM (Salvador)	7	1	8
CPqRR (BH)		1	1
DIREB		1	1
ENSP	7	8	15
IOC	45	10	55
IPEC		1	1
VPEIC		1	1
Outro	3	1	4

ICC (Curitiba)	7		7
ICICT	6		6
**Total**	**84**	**26**	**110**

Na comparação entre os resultados do formulário e os dados da CGPG de Maio/2014, (envolvendo 115 pesquisas), a distribuição das pesquisas por unidades da Fiocruz sofreu uma mudança positiva, tendo em vista que algumas unidades que não contavam com pesquisas vinculadas ao BSM passaram a contar com pesquisadores associados ao convênio, conforme Gráfico 4:

Gráfico 4- ***Comparativo da distribuição das pesquisas por unidade (2013 e 2015)***

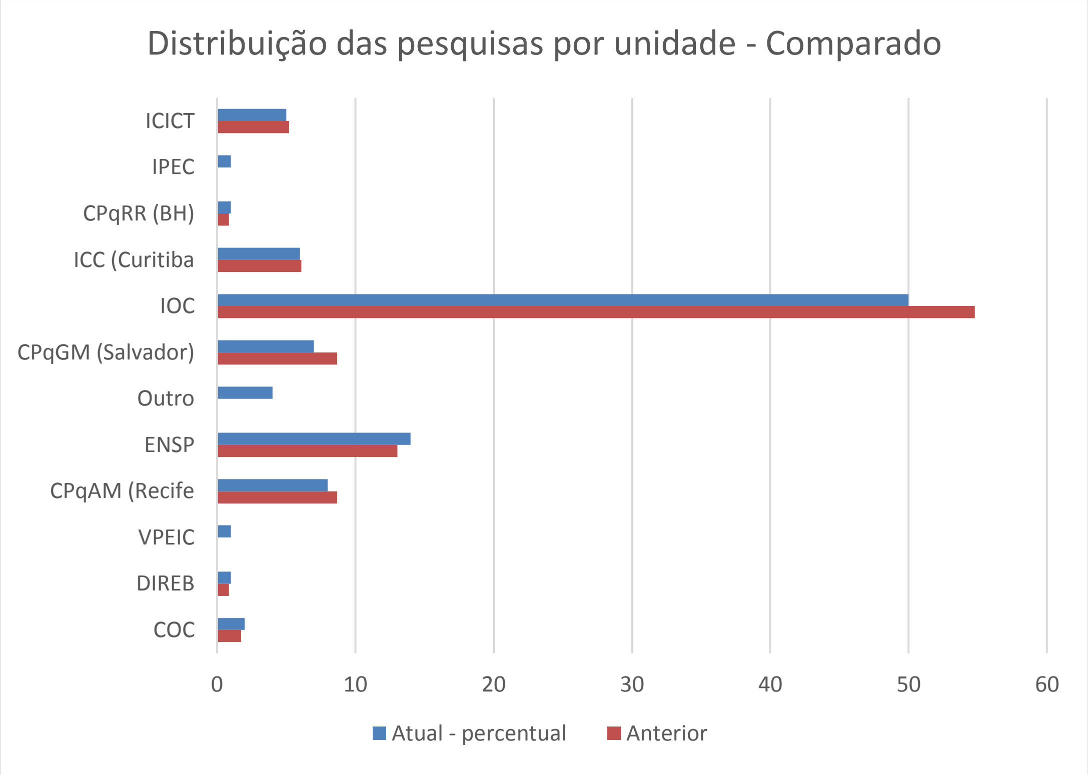

COLABORAÇÃO

O estímulo à colaboração entre pesquisadores do BSM também foi uma estratégia proposta no documento de análise elaborado em 2014. Com os resultados do formulário, verificou-se que apenas 22 pesquisas BSM atualmente contam com alguma colaboração. Nesse grupo, 19 pesquisadores (os quais dizem contar com uma colaboração atual) também indicaram outros pesquisadores/grupos para uma futura colaboração.

Embora o número de pesquisas que não contam com uma colaboração atual seja alto (88 participantes), 46 pesquisadores desse grupo indicaram um potencial colaborador

para sua pesquisa. Sendo assim, o exercício proposto aos pesquisadores de indicarem um potencial colaborador contribuiu para auxiliá-los na identificação de grupos/pesquisas afins, facilitando uma futura integração das pesquisas BSM.

POPULAÇÕES BENEFICIÁRIAS DA PESQUISA

Com o intuito de conhecer as populações beneficiárias das pesquisas em andamento foi solicitado aos pesquisadores BSM que indicassem quais eram esses grupos destinatários em suas pesquisas a partir de uma lista pré-estabelecida (múltipla escolha), contando também com o campo editável "outros" que permitia inserir outros grupos e/ou apresentar especificidades da população beneficiária, como aspectos geracionais, de gênero, raça/cor, necessidades especiais, etc. As opções pré-definidas incluíam: População-alvo do BSM em geral, Indígenas, Moradores de áreas endêmicas, Moradores de comunidades, Profissionais de saúde, Populações em situação de rua e Ribeirinhos. A seguir, apresenta-se a lista de todas as populações beneficiárias citadas pelos pesquisadores no campo "outros":

População de Região de Saúde selecionada do Semiárido brasileiro (Decreto 7508\2011 e outros)
Mulheres vivendo com HIV/Aids em situação de vulnerabilidade social e pobreza
Agricultores
Assentados da reforma agrária
Jovens e trabalhadores de baixa renda
Presidiários
Profissionais de saúde e instâncias de participação social
Estudantes da Educação Básica
Professores do ensino básico
Professores de escola pública
Profissionais de Ensino, Cultura e Serviço Social
Gestores das três esferas administrativas do SUS
Gestores de órgãos de fomento
Gestores das áreas analisadas
Administradores e formadores de políticas públicas nacionais e internacionais

Tendo em vista que todas as pesquisas vinculadas ao BSM deveriam ser orientadas às ações de melhoria das condições de vida das populações brasileiras em situação de miséria e que tais populações não são grupos essencialmente homogêneos, a tarefa de identificar e compreender as particularidades dos grupos beneficiários das pesquisas é peça fundamental no cumprimento dos objetivos do programa.

PRODUTOS, PROCESSOS OU PROPOSTAS DE CARÁTER SOCIAL

Os produtos/processos/propostas podem ser definidos como resultados concretos derivados de uma pesquisa. Os objetivos do BSM têm um caráter social marcante e, consequentemente, os produtos derivados das pesquisas dentro do convênio devem levar em conta essa característica. Assim, os produtos das pesquisas BSM devem ser orientados para a melhoria das condições de vida das populações em situação de miséria, podendo ser aplicados de maneira mais imediata. Ou seja, o diferencial das pesquisas do BSM reside em ir além dos produtos convencionais oriundos de pesquisas acadêmicas (tese, artigos científicos, livros, etc.), mas deve primar pela construção de produtos que possam ser diretamente aplicados no atendimento das demandas das populações beneficiárias, incluindo: medicamentos, material educativo/comunicativo, oficinas, notas técnicas institucionais, Tecnologia Social, entre outros.

A grande maioria dos pesquisadores indicou no formulário mais de um produto/processo derivado de sua pesquisa, sendo que apenas 17 indicaram apenas 1 produto:

Tabela 2- ***Tipo de Produtos/processos derivados das pesquisas BSM***

Produtos/processos BSM	Citados por	Exclusivo
Materiais informativos/comunicativos	32	4
Nota técnica institucional	21	1
Tecnologia social	7	2
Metodologia	19	4
Desenvolvimento de fármaco	1	1
Artigos científicos/ tese	9	1
Não definido	4	4

A combinação mais frequente de produtos foi “nota técnica institucional” e “materiais informativos/comunicativos”, totalizando 16 das indicações. Produtos de caráter estritamente acadêmico como artigos científicos, teses, outras publicações do gênero também foram indicadas por 9 pesquisadores. Apesar de tais produtos serem

essenciais para a divulgação das pesquisas científicas, o formulário pretendia focar apenas nos produtos de caráter social e aplicação mais imediata para as populações beneficiárias do BSM.

Outras opções indicadas pelos pesquisadores incluíram: Oficinas de capacitação de agentes de saúde, indicadores de processo, matriz de avaliação de programas de promoção de saúde e apostilas para consulta dos autores.

CRÍTICAS, SUGESTÕES, DÚVIDAS DOS PESQUISADORES ORGANIZADAS POR TEMA

Dentre os pesquisadores que responderam ao questionário, 52 enviaram sugestões, críticas ou dúvidas, sendo elas analisadas e organizadas por temáticas principais/recorrentes:

1- Questões financeiras - falta de recursos para trabalho de campo, encontros e custeio de materiais/ serviços de terceiros
2- Carência de informações sobre as especificidades das pesquisas BSM
3- Oferecimento de mais disciplinas específicas do BSM e agilidade na validação dos créditos
4- Planejamento das reuniões/encontros entre participantes do BSM com antecedência e um maior número de encontros gerais para trocar experiências
5- Problemas burocráticos - CEP/CONEP, trâmite de documentação, etc., que prejudicam o andamento das pesquisas.

Alguns pesquisadores relataram dificuldades em definir ou reconhecer quem são os grupos/sub-grupos/populações beneficiárias de sua pesquisa. A mesma dificuldade foi relatada por alguns pesquisadores na indicação dos produtos/processos derivados da pesquisa ou na organização das notas técnicas institucionais, questões que deverão ser solucionadas através das ações propostas ao final desse documento.

PARTE II - RESULTADOS PRELIMINARES DAS ESTRATÉGIAS PARA A INTEGRAÇÃO DAS PESQUISAS BSM

As estratégias para otimizar o cumprimento dos objetivos propostos no convênio foram descritas no documento discutido na reunião com os gestores em 2014. Tais estratégias tinham como objetivo uma maior integração das pesquisas BSM com temáticas afins, estímulo ao desenvolvimento de temas não contempladas por essas pesquisas até então, mapeamento de grupos de especialistas no Brasil e exterior que pudessem contribuir para a solução de problemas alvo do BSM, ações para inclusão social na Fiocruz, melhoria das ferramentas de acompanhamento e gestão do Plano na Fiocruz (integração ao Observatório), além do estímulo às pesquisas BSM para formularem soluções (produtos/processos/propostas) que atendessem às necessidades do público alvo do BSM. Também ofereceu recomendações para o desenvolvimento das pesquisas pautados nos conceitos de interseccionalidade, interdisciplinaridade e integração dos saberes.

1- *Lançamento do Edital Fiocruz/BSM, considerando os eixos temáticos e unidades da Fiocruz ainda não contemplados pelo BSM*

2- *Migração ao convênio CAPES/BSM de pesquisas em desenvolvimento*

3- *Encontros periódicos entre os pesquisadores dentro de cada eixo temático*

4- *Redes de especialistas – estímulo à criação de redes de especialistas nas áreas do BSM*

5- *Cooperação Técnica Internacional*

6- *Ação integrada aos Observatórios da Fiocruz de determinantes sociais da saúde, Pesquisa e Desenvolvimento Tecnológico*

7- *Inclusão social na Fiocruz*

8- *Pesquisas orientadas ao desenvolvimento de produtos/propostas ou processos para o BSM*

As estratégias sugeridas para a integração das pesquisas estão sendo colocadas em prática desde a última reunião com os gestores e têm como principais resultados até o momento:

Estratégia 1 e 2 - RESULTADO: Edital para seleção de pós-doutorandos orientados aos eixos temáticos e priorizando áreas da Fiocruz ainda não contempladas com pesquisadores do BSM. As 22 vagas oferecidas no edital

permitiam a migração de pesquisas já em andamento na instituição e alinhadas aos objetivos do BSM.

O Edital de pós doutorado lançado em dezembro/2014 indicou os eixos temáticos, seguindo a orientação do documento elaborado em 2014. No total, foram 70 inscritos, sendo 62 inscrições homologadas. Dentre os selecionados no edital do BSM incluem pesquisadores ligados às unidades que ainda não haviam sido contempladas com bolsas do convênio, como o IFF e INI; unidades que não contavam com participantes em nível do pós-doutorado do BSM, como o ICC e ICICT, além de unidades com poucos participantes no convênio até então, como o CPqRR e DIREB.

Tabela 3 - ***Distribuição das novas pesquisas selecionadas no Edital/2014***

Unidade	Inscritos	Selecionados	Reservas	Sem Bolsa
IOC	22	6	7	
ENSP	12	4		2
CPqRR	10	5	2	
CPqGM	5	1	1	
INI	4	2		
CPqAM	3	1	1	
DIREB	2	1		1
ICC	2	1		
ICICT	1			1
IFF	1	1		

Com isso, a estratégia de expansão do alcance do BSM para unidades da Fiocruz que não haviam sido contempladas no convênio se efetivou através do último Edital de seleção de pós-doutorandos.

Da mesma forma, o edital seguiu a estratégia de estimular a seleção de pesquisas dentro de eixos temáticos que haviam sido pouco explorados, diversificando a produção de conhecimentos dentro do BSM. Além disso, os pareceres emitidos pelos avaliadores levaram em conta a adequação das pesquisas aos eixos temáticos propostos.

Estratégia 3 – RESULTADO: a organização das pesquisas por eixos temáticos através da autodeclaração dos próprios pesquisadores foi possibilitada através do formulário de acompanhamento das pesquisas. A partir desse resultado, poderão ser organizados encontros dentro de cada eixo temático, bem como, oferecidas disciplinas específicas orientadas pelas temáticas.

Estratégia 4 e 8 – RESULTADO: o formulário de acompanhamento contribuiu para que os pesquisadores refletissem sobre seus potenciais colaboradores e sobre os produtos/processos derivados de suas pesquisas.

AÇÕES PARA O ATENDIMENTO DAS DEMANDAS DOS PESQUISADORES E CUMPRIMENTO DAS ESTRATÉGIAS

A partir dos comentários, dúvidas e sugestões emitidas pelos pesquisadores no formulário de acompanhamento do BSM e das estratégias propostas em 2014, foram elaboradas as seguintes ações:

AÇÃO 1: Reunião de boas-vindas aos novos alunos do BSM, buscando elucidar os objetivos e resultados esperados do programa. Uma aula inaugural sobre o caráter social da ciência também deve ser considerada.

AÇÃO 2: Plataforma (espaço no *Moodle*) para estreitar comunicação com outros alunos do BSM e também com a coordenação. Essa ação também facilitará a integração e cooperação entre os pesquisadores em temáticas afins.

AÇÃO 3: Cartilha explicativa sobre o convênio – produtos esperados, população-alvo, eixos temáticos, disciplinas obrigatórias, auxílios financeiros, etc.

AÇÃO 4: Realização de uma “oficina para a elaboração de produtos”, especialmente, sobre as normas de elaboração de notas técnicas.

AÇÃO 5: Organização de um calendário semestral de eventos do BSM (encontros e oficinas)

AÇÃO 6: Divulgação do relatório sobre os resultados gerados pelo formulário do BSM

AÇÃO 7: Oferecimento de disciplinas específicas aos alunos do BSM – disciplinas já estruturadas (apresentadas na reunião com gestores, em novembro/2014) e nova disciplina sobre relações entre Ciência, Tecnologia e Sociedade.

AÇÃO 8: Mapeamento e publicação de ações, produtos ou "boas práticas" do BSM já consolidadas para que possam ser replicadas por outros pesquisadores.

AÇÃO 9: Livro BSM sobre avaliação do programa e boas práticas (principais produtos derivados das pesquisas e seus resultados na comunidade).
